# Role of fronto-limbic circuit in neuropsychiatric symptoms of dementia: clinical evidence from an exploratory study

**DOI:** 10.3389/fpsyt.2024.1231361

**Published:** 2024-05-10

**Authors:** Matteo Cotta Ramusino, Camillo Imbimbo, Marco Capelli, Raffaella Fiamma Cabini, Sara Bernini, Francesca Paola Lombardo, Laura Mazzocchi, Lisa Maria Farina, Anna Pichiecchio, Giulia Perini, Alfredo Costa

**Affiliations:** ^1^ Unit of Behavioral Neurology and Center for Cognitive Disorders and Dementias (CDCD), IRCCS Mondino Foundation, Pavia, Italy; ^2^ Department of Brain and Behavioral Sciences, University of Pavia, Pavia, Italy; ^3^ Department of Mathematics, University of Pavia, Pavia, Italy; ^4^ Pavia Unit, National Institute for Nuclear Physics (INFN), Pavia, Italy; ^5^ Laboratory of Neuropsychology, IRCCS Mondino Foundation, Pavia, Italy; ^6^ Neuroradiology Department, Advanced Imaging and Radiomics Center, IRCCS Mondino Foundation, Pavia, Italy

**Keywords:** neuropsychiatric symptoms, fronto-limbic circuit, cortical thickness, brain volume, cognitive impairment

## Abstract

**Background:**

Neuropsychiatric symptoms (NPSs) are a distressful aspect of dementia and the knowledge of structural correlates of NPSs is limited. We aimed to identify associations of fronto-limbic circuit with specific NPSs in patients with various types of cognitive impairment.

**Methods:**

Of 84 participants, 27 were diagnosed with mild cognitive impairment (MCI), 41 with Alzheimer’s disease (AD) dementia and 16 with non-AD dementia. In all patients we assessed regional brain morphometry using a region of interest (ROI)-based analysis. The mean cortical thickness (CT) of 20 cortical regions and the volume (V) of 4 subcortical areas of the fronto-limbic system were extracted. NPSs were rated with the Neuropsychiatric Inventory (NPI). We used multiple linear regression models adjusted for age and disease duration to identify significant associations between scores of NPI sub-domains and MRI measures of brain morphometry.

**Results:**

All significant associations found were negative, except those between *irritability* and the fronto-opercular regions in MCI patients (corresponding to a 40-50% increase in CT) and between *delusions* and hippocampus and anterior cingulate gyrus (with a 40-60% increase). *Apathy* showed predominant involvement of the inferior frontal regions in AD group (a 30% decrease in CT) and of the cingulate cortex in non-AD group (a 50-60% decrease in CT). *Anxiety* correlated in MCI patients with the cingulate gyrus and caudate, with a CT and V decrease of about 40%, while *hallucinations* were associated with left enthorinal gyrus and right amygdala and temporal pole. *Agitation* showed associations in the AD group with the frontal regions and the temporal pole, corresponding to a 30-40% decrease in CT. *Euphoria, disinhibition* and *eating abnormalities* were associated in the MCI group with the entorhinal, para-hippocampal and fusiform gyri, the temporal pole and the amygdala (with a 40-70% decrease in CT and V). Finally, *aberrant motor behavior* reported a significant association with frontal and cingulate regions with a 50% decrease in CT.

**Conclusion:**

Our findings indicate that specific NPSs are associated with the structural involvement of the fronto-limbic circuit across different types of neurocognitive disorders. Factors, such as age and disease duration, can partly account for the variability of the associations observed.

## 1 Introduction

Dementia is a clinical syndrome characterized by a decline in cognition that interferes with activities of daily living. According to the World Health Organization, around 55 million of people are presently living with dementia worldwide, with a trend to triple by 2050 (1). In addition to cognitive impairment, neuropsychiatric symptoms (NPSs) are a core clinical feature of dementia. These symptoms, often referred to as behavioral and psychological symptoms of dementia (BPSD), broadly include depression, apathy, agitation, psychosis, sleep disturbances and eating abnormalities. NPSs affect almost all demented patients at least once during the disease course, even in early stages, and are associated with accelerated progression to severe dementia, increased risk of institutionalization and earlier death (2, 3). Noteworthy, NPSs appear within the diagnostic criteria of specific types of dementia, such as Dementia with Lewy bodies (DLB) (i.e. visual hallucinations and rapid eye movement (REM) sleep behavior disorders) (4) and the behavioral variant of Frontotemporal Dementia (FTD) (i.e. disinhibition, apathy, aberrant motor behavior, and eating disorders) (5). They are the most distressful aspect of dementia and often lead to lower quality of life for both patients and caregivers (6), although in clinical settings they represent potentially reversible conditions (7). Therefore, early recognition of NPSs in subjects with cognitive impairment is relevant for diagnostic implications, as well as for therapeutic management and disease outcome.

Neuroimaging techniques have been used in cognitive impaired patients to provide clues on the pathophysiology of the most disabling NPSs. Evidence from morphological, perfusion and metabolic studies suggests that alterations in specific cortical regions, predominantly in the anterior cingulate cortex (ACC) and orbitofrontal cortex (OFC), are associated with most of NPSs in patients with AD (8). Similarly, the anterior cingulate and subcortical regions are specifically related to apathy in AD, the anterior cingulate and frontal regions to depression, and the amygdala to anxiety (3). Studies in subjects with MCI are scarce, but reported a link between apathy and hypoperfusion of the temporal and frontal lobes (9), as well as atrophy of the inferior temporal gyrus and anterior cingulate (10). Interestingly, it has been shown that prefrontal subregions and amygdala play a key role in the emotion regulation (11) and are involved in psychiatric diseases such as major depressive disorder (12).

This early evidence focused on single neuropsychiatric symptoms or single diagnostic groups seems to suggest that the limbic lobe and its frontal interconnections are variously involved in the onset of neuropsychiatric symptoms in individuals with cognitive disorders. In this work, we studied the associations between the occurrence of neuropsychiatric symptoms and morphostructural parameters of cortical (cortical thickness) and subcortical (volume) regions in three diagnostic groups that differ in severity and etiology of cognitive decline. The aim is to exploratively investigate the involvement of the fronto-limbic circuit in the full range of neuropsychiatric disorders, taking into account the severity and the type of cognitive decline. The results of this work will provide the basis for future correlation studies focused on specific components of this circuit.

## 2 Materials and methods

This study was approved by the IRCCS Mondino Foundation Ethics Committee (n. 20210032261) and carried out in accordance with the ethical standards of the Helsinki Declaration. All subjects provided written informed consent for image acquisition and anonymized use of their data.

### 2.1 Participants

We enrolled 84 cognitively impaired patients from the Behavioral Neurology Unit of the IRCSS Mondino Foundation (Pavia, Italy), referred to our Institute consecutively between June 2018 and February 2021. We included subjects aged 50 to 90 years referring for first evaluation and subsequently diagnosed with MCI (amnesic or non-amnesic/single or multiple domain) (13) or dementia (behavioral variant of frontotemporal dementia (bvFTD) (5), dementia with Lewy bodies (DLB) (4) or vascular dementia (VD)). Subjects with psychiatric disease, epilepsy or any uncontrolled medical condition that could contribute to cognitive impairment (e.g., nephropathy, liver disease, brain tumor, alcohol or drug abuse, normal pressure hydrocephalus) were excluded. None of the patients were receiving cholinesterase inhibitors, antidepressants or antipsychotic drugs at the time of the assessment.

### 2.2 Study design

**Figure 1:**
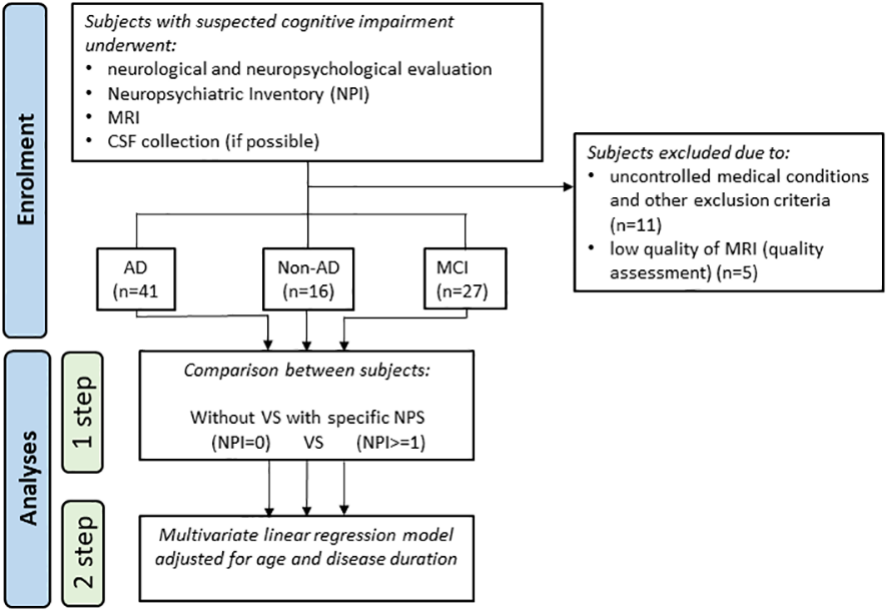
Flowchart of the study design and the performed analyses. CSF, cerebrospinal fluid; MRI, magnetic resonance imaging; NPS, neuropsychiatric symptom.

This study was designed as a single-site cross-sectional case-control study in which the controls were not healthy but demented subjects without NPSs (patients with NPSs versus patients without NPSs). This design was intended to allow for understanding whether the atrophy is associated with NPSs and not just related to the disease or to physiological ageing (the latter is expected to be equally represented in both groups). For the same reason, we investigated the NPSs in different etiological groups, in order to ascertain that atrophy is associated with NPSs in a reliable manner across different etiological groups. As part of the routinely diagnostic workup, all enrolled participants underwent neurological and neuropsychological evaluation, Neuropsychiatric Inventory (NPI) (14) assessment and morphological magnetic resonance imaging (MRI); cerebrospinal fluid (CSF) was collected in seventy-nine subjects (detailed analytic procedure has been previously described in 15). Subjects with dementia had a clinical dementia rating (CDR) score ≥ 1 (16) and received an etiological diagnosis of typical AD [n=41; (17)], bvFTD [n=5; (5)], DLB [n=1; (4)] or VD [n=4; (18)]. All patients with non-vascular dementia had a score < 4 on the Modified Hachinski Ischemic Scale (19). Six demented patients, with negative AD biomarkers, could not receive a diagnosis with sufficient confidence, and were therefore classified into not-otherwise specified dementias (Dem NOS). The flowchart in **Figure 1** summarizes the study design and analyses performed.

### 2.3 Neuropsychological and behavioral assessment

The neuropsychological evaluation included tests for global cognitive efficiency (Mini-Mental State Examination, MMSE), memory (Verbal Span, Digit Span, 15 Item Memory Test, Corsi Test, Story Recall Test, Rey Complex Figure delayed recall), logical and executive functioning (Raven’s Colored Matrices, Frontal Assessment Battery), attention (Trail Making Test A/B, Attentive Matrices, Stroop Test), language (Semantic and Phonemic fluency tests) and visual-spatial perception (Rey Complex Figure copy).

NPI was used to assess behavioral changes associated with dementia. The questionnaire begins with screening questions addressed to the caregiver to investigate whether the patients had experienced any neuropsychiatric symptoms over the past month. In case of positive screening, the caregivers are asked to rate with dedicated scale the frequency (range 1-4), the severity (range 1-3), and their level of distress for the corresponding NPS (range 0-5); the total score for each NPS is the product of the ratings for frequency and severity (14).

### 2.4 Neuroimaging

MRI scans were acquired at the Neuroradiology Unit of IRCCS Mondino Foundation, Pavia. We analyzed eighty-four 3D T1-weighted sequences acquired with Magnetom Skyra 3T (Siemens Healthcare). A 32-channel coil was used for this study. Imaging parameters were: magnetization-prepared rapid acquisition with gradient echo (MPRAGE) with time of repetition = 2300 ms, echo time = 2.98 ms; inversion time = 900 ms; flip angle = 9°; voxel size = 1.0 x 1.0 x 1.0 mm (n = 68) or 1.2 x 1.2 x 1.2 mm, (n = 16) with no interslice gap; matrix size = 256 x 256.

**Figure 2:**
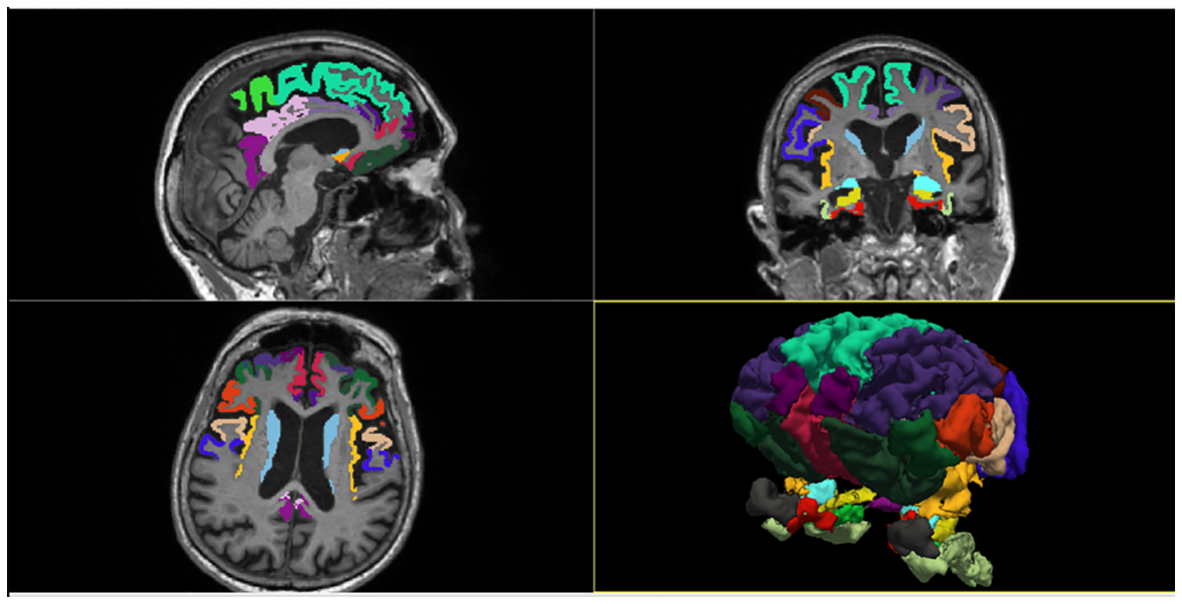
Brain segmentation and Regions of Interest included in the study.

We used the commercial software FreeSurfer v.6 (https://surfer.nmr.mgh.harvard.edu) to evaluate regional brain morphometry on MRI scans. Freesurfer is a set of software tools for the study of cortical and subcortical anatomy. It provides a full processing stream for structural MRI data, including skull stripping, B1 bias field correction and gray-white matter segmentation. It also allows the reconstruction of the cortical surface (by identifying the gray-white matter boundary and pial surface) and the labeling of cortical and subcortical regions. In the present study, we extracted the cortical thickness (CT) of 20 cortical regions and the volume (V) of 4 subcortical regions included in the fronto-limbic system, which were parceled and labeled according to Desikan-Killiany anatomical atlas (**Figure 2**). The earliest descriptions of the anatomical composition of the fronto-limbic circuit date back to the ‘90s (20) and include as major regions: the prefrontal cortex (dorsolateral, ventromedial, and orbitofrontal), amygdala, ventral striatum (nucleus accumbens, caudate), anterior cingulate, and insula. Later, evidence in psychiatric field expanded the areas involved in the fronto-limbic circuitry to include the temporo-lateral regions and the hippocampus (21). Therefore, we placed ROIs in the above regions including also adjacent cortical areas and the posterior portion of the cingulate gyrus, in order to be as inclusive as possible. The chosen 24 regions of interest (ROI) were: Entorhinal cortex, Parahippocampal gyrus, Temporal pole, Fusiform gyrus, Superior frontal gyrus, Middle frontal gyrus (Caudal and Rostral division), Inferior frontal gyrus (Pars opercularis, Pars triangularis, Pars orbitalis), Orbitofrontal cortex (Lateral and Medial division), Frontal pole, Precentral gyrus, Paracentral lobule, Cingulate cortex (Rostral anterior division, Caudal anterior division, Posterior division, Isthmus division), Insula, Accumbens, Amygdala, Caudate, Hippocampus. We adjusted volumes for total intracranial volume (TIV) to account for individual difference in brain size. Imaging results were inspected individually by a trained radiologist (LMF) for quality assessment. An in-house Matlab v.2020b code was then used to extract and store patient-specific anatomical measures of interest for subsequent statistical analysis. CT and subcortical volumes adjusted for TIV were statistically verified to be not significantly dependent from the different T1 MPRAGE spatial resolutions through a Mann-Whitney test (**Supplementary Table 1**).

### 2.5 Statistical analysis

Shapiro-Wilk test was used to investigate the distribution normality of the different variables. Demographic and clinical characteristics among diagnostic groups were compared using Kruskall-Wallis test for continuous variables, and Chi-square test (χ2) for categorical variables.

The following statistical analyses were performed separately in three different diagnostic groups: MCI, AD dementia and non-AD dementia. Differences in CT of cortical ROIs and V of subcortical ROIs between patients presenting with a specific NPS (total NPI sub-domain score ≥ 1) compared to those who did not (total NPI subdomain score = 0) were examined using Mann-Whitney test. Associations between NPI sub-domains scores and CT or V were investigated within each group using multivariate linear regression models adjusted for age, disease duration and MMSE. Since all the statistical tests were conducted for exploratory purposes, no multiple testing correction for the number of comparisons has been applied. Statistical computations were performed using R v. 4.1.2 (The R Foundation for Statistical Computing). A two-sided p-value < 0.05 was considered statistically significant.

## 3 Results

### 3.1 Patients’ characteristics

Demographic and clinical characteristics of the study population are shown in **Table 1**. We considered three different diagnostic groups: MCI (n=27, 13 with CSF biomarkers positive for Alzheimer pathology; age: 73.2 ± 5.7 years), AD dementia (n=41; age: 74.1 ± 7.1 years) and non-AD dementia (n=16; age: 72.0 ± 7.3 years). Non-AD dementia group included 5 patients with FTD, 1 DLB, 4 VD and 6 Dem NOS (**Supplementary Table 2**). Mean MMSE score was 26.8 ± 2.1 in subjects with MCI, 18.4 ± 5.0 in patients with AD dementia and 20.3 ± 4.6 in those with non-AD dementia. No significant difference was found among diagnostic groups with respect to age, gender and education. As expected, AD group showed lower Aβ_42_ levels, and higher p-tau and t-tau levels, compared to non-AD and MCI groups. The composite score for each item of the NPI, along with distribution of NPSs within the diagnostic groups are shown in **Table 2**. Depression and anxiety were the most prevalent NPSs in the MCI and AD-dementia groups (55.6% and 63.4%, respectively), while in non-AD patients apathy was the most common (68.8%). When considering all groups, more than 80% of the subjects had two or more NPSs at one time. The prevalence of apathy, night-time behavior disturbances and hallucinations significantly differed between the 3 groups of patients (p = 0.014, p = 0.035, p = 0.011 respectively). **Supplementary Table 3** includes mean and standard deviation (SD) of CT (mm) and V (mm3) of the ROIs in AD, non-AD and MCI patients.

**Table 1:** Sociodemographic characteristics and biomarker measures.

	AD (n=41)	non-AD (n=16)	MCI (n=27)	p-value^a^
Age, mean (SD), y	74.1 (7.1)	72.0 (7.3)	73.2 (5.7)	0.50
Female, N. (%)	27 (66)	6 (35.3)	12 (44.4)	0.06
Education, mean (SD), y	7.6 (3.7)	8.2 (3.5)	8.8 (3.4)	0.30
Disease duration, mean (SD), y	2.7 (2.9)	3.2 (3.2)	1.7 (1.4)	0.46
MMSE, mean (SD)^b^	18.4 (5.0)	20.3 (4.6)	26.8 (2.1)	<0.001
CDR 0.5 1 2 3	0 (0%) 27 (65.9%) 8 (19.5%) 6 (14.6%)	0 (0%) 12 (75%) 3 (18.8%) 1 (6.3%)	27 (100%) 0 0 0	<0.001
CSF biomarkers, mean (SD), pg/mL Aβ42^b^ t-tau^b^ p-tau^b^	526.9 (229.6) 859.4 (466.8) 120.7 (71.2)	801.3 (352.7) 325.9 (278.3) 48.6 (40.7)	658.3 (267.6) 436.4 (220.0) 63.0 (30.0)	<0.001 <0.001 <0.001

AD, Alzheimer’s disease; CDR, Clinical dementia rating scale; CSF, cerebrospinal fluid; MCI, mild cognitive impairment; MMSE, Mini-Mental State Examination. a) Significance tests used were Chi-square test for categorical variables and Kruskal–Wallis test for continuous variables. b) Post hoc pair-wise comparisons with Bonferroni correction of diagnostic groups: MMSE: MCI > AD and non-AD (p < 0.001); CDR: AD > MCI (p < 0.001), MCI < non-AD (p < 0.001), AD vs non-AD (p = 0.414); Aβ_1–42_: AD < non-AD (p < 0.01); p-tau: AD > non-AD and MCI (p < 0.001); t-tau: AD > non-AD and MCI (p < 0.001).

**Table 2:** Percentage of patients presenting with specific NPSs and mean and standard deviation of the sub-domain NPI total score in AD, non-AD and MCI groups.

NPSs	AD (n=41)			non-AD (n=16)			MCI (n=27)			
	+NPS vs -NPS	%	mean (SD) score	+NPS vs -NPS	%	mean (SD) score	+NPS vs -NPS	%	mean (SD) score	p-value^a^
Delusions	15 vs 26	36.6	3.1 (7.5)	3 vs 13	18.8	1.7 (5.2)	4 vs 23	14.8	0.9 (5.6)	0.110
Depression	26 vs 15	63.4	4 (7.1)	9 vs 7	56.3	2.4 (4.7)	15 vs 12	55.6	2.5 (5.7)	0.780
Eating abnormalities	20 vs 21	48.8	3.6 (7.3)	7 vs 9	43.8	2.9 (5)	6 vs 21	22	1.2 (5.6)	0.083
Apathy^b^	22 vs 19	53.7	3.4 (7.2)	11 vs 5	68.8	3.9 (5.1)	7 vs 20	25.9	1.2 (5.6)	0.014
Euphoria	6 vs 35	14.6	0.7 (6.6)	3 vs 13	18.8	0.4 (3.9)	5 vs 22	18.5	0.6 (5.5)	0.890
Night-time behaviour disturbances^b^	19 vs 22	46.3	2.7 (7.0)	2 vs 14	12.5	0.9 (4.4)	9 vs 18	33.3	1.6 (5.6)	0.035
Agitation	23 vs 18	56.1	2.9 (7.1)	8 vs 8	50	1.6 (4.2)	11 vs 16	40.7	2 (5.6)	0.460
Aberrant motor behaviour	13 vs 28	31.7	2.3 (7.3)	3 vs 13	18.8	0.6 (4.0)	5 vs 22	18.5	1.1 (5.7)	0.380
Anxiety	26 vs 15	63.4	3.4 (7.2)	5 vs 11	31	1.7 (4.8)	15 vs 12	55.6	2.2 (5.6)	0.090
Hallucinations^b^	11 vs 30	26.8	1.9 (7.1)	2 vs 14	12.5	0.8 (4.7)	0 vs 27	0	0 (0)	0.011
Disinhibition	12 vs 29	29.3	1 (6.5)	7 vs 9	43.8	1.3 (3.9)	5 vs 22	18.5	1.2 (5.8)	0.200
Irritability	21 vs 20	51.2	2.5 (7.2)	7 vs 9	43.8	2.1 (4.5)	13 vs 14	48.1	1.7 (5.5)	0.880

AD, Alzheimer’s disease; MCI, mild cognitive impairment; NPS, neuropsychiatric symptom. a) The Chi-square test was used to test differences in NPS prevalence among AD, non-AD and MCI groups. b) Post hoc pair-wise comparisons with Bonferroni correction of diagnostic groups: Apathy: non-AD vs MCI (p = 0.030); Night-time behaviour disturbance: AD vs non-AD (p = 0.045); Hallucinations: AD vs MCI (p = 0.067).

### 3.2 Differences in fronto-limbic system morphometry between patients with and without specific NPSs

Significant differences in morphometric measures (CT and V of cortical and subcortical ROI) between patients with and without specific NPSs are shown in **Table 3**. For each NPS, at least one anatomic region of the 24 investigated reported a CT or V decrease, except for hallucinations for which no differences were found. *Agitation* in AD patients and *aberrant motor behavior* in non-AD patients were the NPSs associated with the higher number of atrophic ROI (11 regions for the former and 12 for the latter), without a clear hemispheric predominance but with prevalent involvement of the fronto-orbital, opercular and medial temporal regions. *Delusions* showed more atrophy in the fronto-orbital regions in AD and non-AD groups, *depression* in fronto-lateral and anterior cingulate regions in non-AD group, and *apathy* in fronto-orbital regions in AD group and in fronto-insular regions in non-AD group. The MCI group had fewer NPSs associated with atrophic regions compared to the AD and non-AD groups, and these differences were not only expressed in term of atrophy; *apathy*, *night-time behavior disturbances*, *agitation* and *irritability* were indeed associated to increased CT or V in specific ROIs, such as the anterior cingulate cortex, the caudate and accumbens nuclei and the rostral middle frontal gyrus.

**Table 3:** Differences in CT of cortical ROIs and V of subcortical ROIs between patients with (total NPI sub-domain score ≥ 1) or without specific NPSs (total NPI sub-domain score = 0).

NPSs	AD (n=41)		non-AD (n=16)		MCI (n=27)	
	ROIs	*p*-value	ROIs	*p*-value	ROIs	*p*-value
Delusions	Lateral orbito-frontal - L Entorhinal - L	0.035 0.046	Lateral orbito-frontal - R Medial orbito-frontal - R Hippocampus - R	0.036 0.048 0.048	-	-
Depression	-	-	Superior frontal - L Caudal middle-frontal - L Pars opercularis - L Caudal anterior-cingulate - L Isthmus cingulate - L Caudate - R	0.031 0.002 0.012 0.012 0.023 0.023	-	-
Eating abnormalities	-	-	Para hippocampal - R	0.042*	-	-
Apathy	Superior frontal - R Pars opercularis - R Pars orbitalis - R Pars orbitalis - L Pars triangularis - R	0.026 0.024 0.003 0.023 0.028	Superior frontal - L Rostral middle-frontal - L Pars triangularis - L Medial orbito-frontal - L Insula - R Insula - L	0.038 0.027 0.041 0.013 0.038 0.038	Caudal anterior cingulate-R	0.038*
Euphoria	Rostral middle-frontal - L Lateral orbito-frontal - L Insula - L Entorhinal – R	0.037 0.004 0.041* 0.036*	Amygdala - L	0.039*	Pars orbitalis - L Para hippocampal - L	0.036 0.019
Night-time behaviour disturbances	Paracentral – R	0.047	Frontal pole - R	0.047	Caudate – L Caudal anterior cingulate-R	0.046* 0.019*
Agitation	Pars orbitalis - R Pars orbitalis - L Pars triangularis - R Insula - R Entorhinal - R Entorhinal - L Para hippocampal - L Temporal pole - R Temporal pole – L Fusiform - L Amygdala - R Caudate - R Caudate - L	0.024 0.050 0.046 0.016 0.044 0.009 0.031 0.006 0.024 0.025 0.029 0.027* 0.014*	-	-	Isthmus cingulate - R Accumbens - L	0.008 0.039*
Aberrant motor behaviour	Entorhinal - L Temporalpole - L	0.015 0.033	Superior frontal - L Rostral middle-frontal - R Rostral middle-frontal - L Pars opercularis - R Pars opercularis - L Pars triangularis - R Pars triangularis - L Lateral orbito-frontal - R Lateral orbito-frontal - L Medial orbito-frontal - R Accumbens - R Hippocampus - R	0.039 0.014 0.025 0.039 0.014 0.043 0.037 0.011 0.004 0.014 0.014 0.014	-	-
Anxiety	-	-	-	-	Rostral anterior cingulate -L Posterior cingulate - R	0.014 0.047
Hallucinations	-	-	-	-	-	-
Disinhibition	Accumbens - R	0.011*	-	-	Para hippocampal - L	0.047
Irritability	Posterior cingulate - R	0.049	-	-	Rostral middle-frontal - R	0.029*

To make the table easier to read, only the results that are statistically significant have been included. The full results are provided in **Supplementary Table 4**. AD, Alzheimer’s disease; MCI, mild cognitive impairment; NPS, neuropsychiatric symptom; R, right; L, left. All reported ROIs have lower values in CT and/or V in patients with neuropsychiatric symptoms, except those indicated by the asterisk (*).

### 3.3 Associations between fronto-limbic circuit morphometry and NPSs

**Tables 4**, **5** report significant associations between total score of each NPI subdomain and CT or V of the cortical and subcortical ROIs, after correction for covariates in multiple linear regression models. All significant associations found in demented patients were generally negative (inverse), while in the MCI group positive associations were found between *irritability* and fronto-opercular regions (corresponding to a 40-50% increase in CT for each 100% increase in NPI score) and between *delusions* and hippocampus and anterior cingulate gyrus (corresponding to a 40-60% increase). *Apathy* showed more atrophic regions in demented patients than MCI subjects, with predominant involvement of the inferior frontal regions in AD group (corresponding to a 30% decrease in CT) and of the cingulate cortex in non-AD group (about a 50-60% decrease in CT). *Anxiety* correlated in MCI patients with the cingulate gyrus and caudate, with a CT and V decrease of about 40%, while *hallucinations* were associated with left enthorinal gyrus and right amygdala and temporal pole when the analyses were also corrected for MMSE. *Agitation* showed associations in the AD group with the frontal regions (superior, middle and inferior frontal gyri) and the temporal pole, corresponding to a 30-40% decrease in CT. *Euphoria, disinhibition* and *eating abnormalities* had significant associations predominantly in the MCI group. The ROIs mainly associated with these NPSs were the entorhinal, para-hippocampal and fusiform gyri, the temporal pole and the amygdala, with *euphoria* having a 40-70% decrease in CT and V, *dishinibition* a 50% decrease and *eating abnormalities* a 40-60% decrease. Finally, *aberrant motor behavior* reported a significant association with frontal and cingulate regions with a 50% decrease in CT.

**Table 4:** Associations between NPI sub-domains scores (numerical variables) and CT of cortical ROIs and V of subcortical ROIs.

NPSs	AD (n=41)			non-AD (n=16)			MCI (n=27)		
	ROIs	*b*	*p*-value	ROIs	*B*	*p*-value	ROIs	*b*	*p*-value
Delusions	Pars orbitalis - R Entorhinal - L	-0.336 -0.356	0.034 0.037	-	-	-	-	-	-
Depression	-	-	-	-	-	-	Paracentral - L	-0.446	0.023
Eating abnormalities	Pars opercularis - R	-0.337	0.040	Insula - L	-0.571	0.032	Caudal middle frontal - R Entorhinal - R Para-hippocampal - R Temporal pole - R Temporal pole - L Fusiform - R Amygdala - R	-0.460 -0.501 -0.493 -0.680 -0.466 -0.470 -0.603	0.050 0.017 0.021 0.001 0.025 0.035 0.005
Apathy	Pars opercularis - R Pars triangularis - R Pars triangularis - L Precentral - L	-0.329 -0.386 -0.320 -0.387	0.045 0.022 0.047 0.024	Superior frontal - L Pars opercularis - R Posterior cingulate - L Isthmus cingulate – L Caudate - R	-0.591 -0.626 -0.513 -0.553 -0.581	0.020 0.014 0.044 0.037 0.029	Paracentral-L	-0.491	0.021
Euphoria	-	-	-	-	-	-	Entorhinal - R Para-hippocampal - R Caudal middle frontal - R Rostral anterior cingulate - R Temporal pole - R Temporal pole - L Fusiform - R Amygdala - R Amygdala - L	-0.530 -0.496 -0.471 -0.468 -0.709 -0.463 -0.543 -0.677 -0.452	0.012 0.022 0.047 0.023 0.01 0.028 0.015 0.001 0.040
Night-time behaviour disturbances	Precentral - L Paracentral - R	-0.329 -0.369	0.046 0.029	Frontal pole - R	-0.572	0.014	Amygdala-R	-0.485	0.029
Agitation	Temporal pole - R Superior frontal - R Caudal middle frontal - R Pars opercularis - R Pars triangularis - R	-0.395 -0.336 -0.358 -0.451 -0.457	0.023 0.047 0.028 0.004 0.006	-	-	-	-	-	-
Aberrant motor behaviour	Entorhinal-L	-0.351	0.039	Hippocampus - R	-0.514	0.050	Rostral middle frontal - R Rostral anterior cingulate – R	-0.460 -0.516	0.049 0.011
Anxiety	-	-	-	-	-	-	Rostral anterior cingulate – L	-0.409	0.033
Hallucinations	-	-	-	-	-	-	-	-	-
Disinhibition	-	-	-	-	-	-	Entorhinal - R Temporal pole - R Fusiform - R Amygdala-R	-0.482 -0.505 -0.536 -0.463	0.024 0.015 0.016 0.039
Irritability	Temporal pole - R	-0.349	0.045	Medial orbito-frontal - R	-0.608	0.035	Rostral middle frontal - R Rostral middle frontal - L Pars opercularis - R Pars triangularis - L	0.505 0.441 0.462 0.450	0.022 0.038 0.022 0.029

Coefficients and p-values of the multivariate linear regression adjusted for age and disease duration are shown.

To make the table easier to read, only the results that are statistically significant have been included. The full results are provided in **Supplementary Table 5**. AD, Alzheimer’s disease; MCI, mild cognitive impairment; NPS, neuropsychiatric symptom; R, right; L, left.

**Table 5:** Associations between NPI sub-domains scores (numerical variables) and CT of cortical ROIs and V of subcortical ROIs.

NPSs	AD (n=41)			non-AD (n=16)			MCI (n=27)		
	ROIs	*b*	*p*-value	ROIs	*b*	*p*-value	ROIs	*b*	*p*-value
Delusions	Pars orbitalis - R Entorhinal - L	-0.332 -0.380	0.039 0.029	Temporal pole - R	0.602	0.049	Pars triangularis – L Rostral anterior cingulate – L Hippocampus – R Hippocampus - L	0.462 0.514 0.624 0.482	0.036 0.033 0.012 0.041
Depression	-	-	-	-	-	-	Paracentral – L Posterior cingulate – L	-0.386 0.384	0.039 0.049
Eating abnormalities	Pars opercularis - R	-0.347	0.044	Insula - L	-0.639	0.022	Caudal middle frontal - R Entorhinal - R Para-hippocampal - R Temporal pole - R Temporal pole - L Fusiform - R Amygdala – R Amygdala - L	-0.532 -0.499 -0.508 -0.668 -0.476 -0.468 -0.626 -0.443	0.025 0.018 0.018 0.001 0.022 0.037 0.003 0.048
Apathy	Pars triangularis - R Precentral - L	-0.360 -0.356	0.037 0.044	Pars opercularis - R	-0.629	0.041	Paracentral-L Accumbens area - L	-0.451 0.448	0.034 0.037
Euphoria	-	-	-	-	-	-	Entorhinal - R Para-hippocampal - R Caudal middle frontal - R Rostral anterior cingulate - R Temporal pole – R Parahippocampal - R Temporal pole - L Fusiform - R Amygdala - R Amygdala – L Hippocampus - R	-0.525 -0.521 -0.580 -0.415 -0.673 -0.521 -0.480 -0.539 -0.714 -0.540 -0.493	0.01 0.012 0.012 0.047 0.000 0.012 0.018 0.012 0.000 0.011 0.043
Night-time behaviour disturbances	Paracentral - R	-0.363	0.039	Frontal pole – R Lateral orbito-frontal - R	-0.610 -0.629	0.011 0.029	Amygdala-R	-0.507	0.023
Agitation	Temporal pole - R Caudal middle frontal - R Pars opercularis - R Pars triangularis - R	-0.386 -0.344 -0.440 -0.439	0.029 0.04 0.008 0.01	Lateral orbito-frontal -R	-0.660	0.04	-	-	-
Aberrant motor behaviour	Entorhinal-L	-0.362	0.038	Temporal pole - R	0.479	0.039	Rostral middle frontal – R Rostral middle frontal – L Rostral anterior cingulate – R Superior frontal – R Isthmus cingulate – L Accumbens area - L	-0.533 -0.466 -0.503 -0.457 0.502 -0.436	0.026 0.045 0.017 0.044 0.029 0.048
Anxiety	-	-	-	-	-	-	Isthmus cingulate – L Caudate – R	-0.449 -0.436	0.024 0.021
Hallucinations	Entorhinal - L	-0.348	0.043	Temporal pole – R Amygdala - R	0.549 0.521	0.032 0.032	-	-	-
Disinhibition	Insula - L	0.478	0.005	-	-	-	Entorhinal - R Temporal pole - R Fusiform - R Amygdala-R	-0.481 -0.502 -0.535 -0.477	0.027 0.019 0.018 0.037
Irritability	–	–	–	Medial orbito-frontal - R	-0.634	0.025	Rostral middle frontal - R Rostral middle frontal - L Pars opercularis - R Pars triangularis - L	0.575 0.526 0.500 0.466	0.011 0.016 0.014 0.025

Coefficients and p-values of the multivariate linear regression adjusted for age, disease duration and MMSE are shown.

To make the table easier to read, only the results that are statistically significant have been included. The full results are provided in **Supplementary Table 6**. AD, Alzheimer’s disease; MCI, mild cognitive impairment; NPS, neuropsychiatric symptom; R, right; L, left.

Based on the results that emerged in **Table 3**, cortical and subcortical ROIs were grouped into two main subcircuits included within the fronto-limbic circuit: circuit 1 (frontal and cingulate regions) including Superior frontal gyrus, Middle frontal gyrus (Caudal and Rostral division), Inferior frontal gyrus (Pars opercularis, Pars triangularis, Pars orbitalis), Orbitofrontal cortex (Lateral and Medial division), Cingulate cortex (Rostral anterior division, Caudal anterior division, Isthmus division), Insula, Caudate; and circuit 2 (temporal regions) including Entorhinal cortex, Parahippocampal gyrus, Temporal pole, Fusiform gyrus, Amygdala, Hippocampus. Significant associations between total score of each NPI subdomain and V of these two circuits are reported in **Table 6**. In MCI group, *euphoria* was associated with a 50% decrease in circuit 2 on both hemispheres, while *anxiety* was associated with a 40% decrease in circuit 1 on the right hemisphere. Conversely, *irritability* was associated with a 50% increase in circuit 1 on both hemispheres. These results and their relative significance depend on the number and type of regions included by the two circuits and should therefore be considered as a supplement to the previous analyses.

**Table 6:** Associations between NPI sub-domains scores (numerical variables) and volume of brain circuits composed by selected cortical and subcortical ROIs.

NPSs	AD (n=41)			non-AD (n=16)			MCI (n=27)		
	Circuit	*b*	*p*-value	Circuit	*b*	*p*-value	Circuit	*b*	*p*-value
Euphoria	-	-	-	-	-	-	2 - R 2 - L	-0.594 -0.487	0.004 0.02
Anxiety	-	-	-	-	-	-	1 - R	-0.392	0.029
Irritability	-	-	-	-	-	-	1 - R 1 - L	0.543 0.551	0.005 0.004

Coefficients and p-values of the multivariate linear regression adjusted for age and disease duration are shown.

ROIs of Circuit 1: superior frontal gyrus, caudal middle frontal gyrus, rostral middle frontal gyrus, pars opercularis, pars triangularis, pars orbitalis, lateral orbital frontal cortex, medial orbital frontal cortex, rostral anterior division of cingulate cortex, caudal anterior division of cingulate cortex, isthmus division of cingulate cortex, insula, caudate nucleus. ROIs of Circuit 2: entorhinal cortex, parahippocampal gyrus, temporal pole, fusiform gyrus, amygdala, hippocampus. AD, Alzheimer’s disease; MCI, mild cognitive impairment; NPS, neuropsychiatric symptom; R, right; L, left.

## 4 Discussion

In this study, we investigated potential associations between NPSs, assessed with the NPI questionnaire, and cortical thickness (CT) and volume (V) of 24 cerebral regions of the fronto-limbic circuit, in a cohort of patients with MCI, AD dementia and non-AD dementia. We found that different alterations in the fronto-cingulate and temporal regions were associated with presence and severity of NPSs across the different types of neurocognitive disorders. These results highlight the key role of the fronto-limbic circuit in the pathophysiology of the most frequent neuropsychiatric manifestations in cognitive disorders.

Previous neuroimaging studies reported that apathy, depression and delusions were the most prevalent NPSs associated with brain changes in AD (8). *Apathy* has been related to alterations of the frontal-subcortical networks, with a more severe and frequent involvement of the anterior cingulate and orbitofrontal cortices, as well as the putamen and caudate nucleus (22, 23). Our results confirmed these associations, supporting the relevance of the fronto-basal regions in modulating the behavioral initiation and reward mechanisms. *Depression* is known to be linked to lesions of cortical-limbic pathways (24), mainly in the dorsolateral prefrontal, cingulate and inferior temporal cortices, hypothalamus, hippocampus and insula (3). In our study, we found similar results in non-AD patients when we compared patients with and without depression (although significance did not survive correction for multiple comparisons). Conversely, we observed a negative association with the thickness of the left paracentral lobule in MCI patients and none in the AD group. Because depression has been associated in MCI subjects with atrophy of regions commonly affected by AD (frontal, parietal and temporal) (25), it is possible that this overlap of the cortical regions involved in depression and in AD attenuates the significance when comparing depressed and non-depressed patients within the AD group. Depression and apathy seem to involve more the frontal and opercular regions and the anterior portion of the cingulate (*cingulate-opercular area*): indeed, the fronto-opercular region and insula play an important role in the emotional control of the subject. In carrying out this function, the raw and instinctual sensory and emotional afferents coming from the hippocampus-amygdala region are filtered by the frontal and insular cortices, which play a role of inhibitory barrier and of modulation (11, 26, 27).

Concerning *delusions* in AD patients, we reinforced the evidence supporting the involvement of the right frontal, especially the orbitofrontal, and temporal structures (i.e. hippocampus, entorhinal cortex, amygdala) (*fronto-temporal area*) (21). These regions are key components of the dopaminergic, mesolimbic and mesocortical pathways, known to be involved in the control of addictive compulsive and obsessive behaviors. It has also been proposed that lesions of the right frontal and prefrontal regions may induce release and hyperactivity of the corresponding preserved contralateral frontal regions, leading to generate a creative narrator from monitoring self, memory and reality, and thus to excessive and false explanations and delusions (28).

*Agitation, aberrant motor behavior, disinhibition, euphoria*, and *irritability*, symptoms included in the “hyperactivity syndrome” (29), were associated to alterations in multiple fronto-limbic regions, including ACC, OFC, inferior frontal gyrus, entorhinal cortex, hippocampus and amygdala. Other neuroimaging findings support these results (30, 31), corroborating the importance of neurodegeneration processes affecting the anterior salience network, that may reduce capacity to process and generate appropriate behavioral responses to salient stimuli (32). Our results agree with the data from the literature; in particular, agitation and aberrant motor disorder display a heterogeneous involvement of the fronto-temporal regions, while euphoria, disinhibition and eating abnormalities were associated with the entorhinal, para-hippocampal and fusiform gyri and the amygdala. This evidence supports the idea that *fronto-temporal and cingulate-opercular areas* should not be thought of as discrete and functionally independent areas, but as interconnected parts of a single circuit that operate for a proper emotional and behavioral functioning. Interestingly, in MCI subjects we found a positive association between irritability and CT of frontal and prefrontal regions; although counterintuitive, other studies obtained similar findings, indicating that in early stages mechanisms other than atrophy may be involved in the development of this symptom, such as, for instance, enhanced connectivity or increased activity (33).

In the associations between NPSs and regional correlates, we found some degree of variability among the three diagnostic groups (AD, non-AD, and MCI). In particular, few differences between patients with and without NPSs were observed in the MCI group, compared with the other two groups. This could be partly explained by both the lower levels of atrophy (which to a given extent is influenced by duration and severity of disease) and the lower frequency and severity of some NPSs in this group (see frequencies and NPI scores in **Table 2**). Conversely, the higher frequency of NPSs and higher NPI scores in the AD group may have allowed for differences in more regions, e.g., for symptoms such as agitation, night-time behavior disturbances, hallucinations and disinhibition. Similarly, the skewed distribution of the two groups (NPS+ versus NPS-) is another factor that might have sometimes reduced the number of associated cortical regions (e.g., in the case of euphoria). In addition, for some symptoms, such as depression, regional patterns of atrophy are described that partly overlap with disease-specific ones: thus, the differences between patients with and without NPS could be mitigated. Regression analyses in the three diagnostic groups (AD, non-AD, and MCI) showed that the number of regional correlates is lower than that obtained from comparisons when corrections for disease duration and MMSE score are made. This is an expected result as we know that the neurodegenerative process is dependent on time and disease process severity, of which cognitive impairment can be considered an indirect measure.

Overall, the fronto-limbic system seems to be characterized by multiple points of vulnerability, on which the neurodegenerative processes can act, partly in a pathology-dipendent and partly in a time-dipendent way. The underlying etiology of cognitive decline and the specific distribution of its neurodegenerative process clearly influence some of the significant correlations observed in this and other studies. For instance, a higher number of temporal structures were involved in AD compared to predominant involvement of the frontal lobe in the non-AD group, which includes FTD and VD. However, the wide variability of the evidence observed in morphometry studies suggests that the occurrence of NPSs may sometimes be associated with regions not directly involved by pathology but affected by functional changes in the brain (e.g., synaptic disconnection in the white matter). Moreover, beyond the different etiological composition of the study population, also intrinsic characteristics of the subjects (genetic, epigenetic, cultural and environmental) could modulate the expression of NPSs and their neuroimaging correlates.

*Limitations.* The main limitation of this study is represented by the absence of a healthy control group and the heterogeneous composition of non-AD group. The choice of a cross-sectional case-control study design in which the controls were not healthy but patients without NPSs was aimed to investigate whether the atrophy is associated with NPSs and not just related to the disease or to physiological ageing (both expected to be equally represented in both cases and controls). For the same reason, we investigated the NPSs in different etiological groups, in order to ascertain whether atrophy was associated with NPSs in a reliable manner across different etiological groups. Some types of dementias are known to be characterized by specific NPSs, and these are purposely included in the diagnostic criteria (e.g. apathy in FTD, and hallucinations in LBD). The variable contribution of these nosological entities to the non-AD group may have affected the percent presentation of NPSs and the significance of the correlations within this group. Second, the cross-sectional study design may have limited the significance of some associations, as the prevalence of neuropsychiatric disorders observed at baseline may have in fact been lower than that recorded after adequate follow-up. Prospective studies could improve the strength of evidence in this regard. Finally, the statistical analyses of this study were conducted for exploratory purposes to provide a preliminary indication of which structural features or regions of interest may be associated with NPSs in neurocognitive disorders. The results of the exploratory investigation were then reinforced by regression analysis and, taken together, may guide future investigations targeting individual components of the fronto-limbic circuit and/or individual NPSs based on more appropriate designs.

*Conclusion.* The results of this study indicate that specific NPSs are associated with the structural involvement of areas of the fronto-limbic circuit across different types of neurocognitive disorders with different severity, as even recently reported in a neuropathological study (34). Previous studies provided information limited to single NPSs or brain regions, never providing a comprehensive view of the fronto-limbic circuit. Moreover, much of the evidence in the literature comes from studies in patients with psychiatric disorders, such as borderline personality disorder, major depression, etc., and not with cognitive disorders. Our work supports the hypothesis that this circuit exerts an important role in the pathophysiology of the most frequent neuropsychiatric manifestations through a gain/loss of function, even if the contribution of other circuits cannot be excluded. Further neuroimaging studies are warranted to explain the heterogeneity of the correlations observed between NPSs and MR findings, as well as to investigate the biological factors (e.g., diet, physical education, vascular risk factors, genetics and epigenetics) that may influence the structure and function of cortical and subcortical regions and modulate strength and direction of the correlations.

## Data availability statement

The original contributions presented in the study are included in the article/**Supplementary Material**, further inquiries can be directed to the corresponding author/s.

## Ethics statement

The studies involving humans were approved by IRCCS Mondino Foundation Ethics Committee (n. 20210032261) . The studies were conducted in accordance with the local legislation and institutional requirements. The participants provided their written informed consent to participate in this study.

## Author contributions

Conceptualization: MC, GP, AC. Methodology: MC, GP, MC, LF, AP. Formal analysis and investigation: MC, GP, CI, RC, FL, LM. Writing - original draft preparation: MC, GP, CI. Writing - review and editing: MC, GP, AC. Resources: AC. Supervision: AC. All authors contributed to the article and approved the submitted version.
