# Supplementary material for: Role of fronto-limbic circuit in neuropsychiatric symptoms of dementia: clinical evidence from an exploratory study
Source: Front Psychiatry. 2024 May 10;15:1231361. doi: 10.3389/fpsyt.2024.1231361 (PMC11119745; doi:10.3389/fpsyt.2024.1231361)
Supplement: Supplementary file 1 [file DataSheet_1.docx]

**Supplementary Material**

**Table S1**.

We statistically verified that all the radiological parameters (20 cortical thickness regions for each hemisphere and 8 volumes of subcortical areas) do not significantly depend by the voxel size, through a Mann-Whitney test with Bonferroni multiple testing correction. For completeness, we attach the test results:

|  | **Row p-value** | **Adjusted p-value** |
| --- | --- | --- |
| 1. superiorfrontal..Right | 0.678 | 32.523 |
| 2. superiorfrontal.Left | 0.409 | 19.626 |
| 3. caudalmiddlefrontal.Right | 0.833 | 39.988 |
| 4. caudalmiddlefrontal.Left | 0.324 | 15.574 |
| 5. rostralmiddlefrontal.Right | 0.815 | 39.137 |
| 6. rostralmiddlefrontal.Left | 0.620 | 29.771 |
| 7. parsopercularis.Right | 0.968 | 46.473 |
| 8. parsopercularis.Left | 0.842 | 40.416 |
| 9. parsorbitalis.Right | 0.484 | 23.212 |
| 10. parsorbitalis.Left | 0.449 | 21.539 |
| 11. parstriangularis.Right | 0.491 | 23.553 |
| 12. parstriangularis.Left | 0.711 | 34.138 |
| 13. lateralorbitofrontal.Right | 0.737 | 35.368 |
| 14. lateralorbitofrontal.Left | 0.330 | 15.844 |
| 15. medialorbitofrontal.Right | 0.150 | 7.180 |
| 16. medialorbitofrontal.Left | 0.449 | 21.539 |
| 17. frontalpole.Right | 0.645 | 30.938 |
| 18. frontalpole.Left | 0.869 | 41.703 |
| 19. precentral.Right | 0.941 | 45.167 |
| 20. precentral.Left | 0.653 | 31.332 |
| 21. paracentral.Right | 0.745 | 35.781 |
| 22. paracentral.Left | 0.277 | 13.279 |
| 23. rostralanteriorcingulate.Right | 0.196 | 9.410 |
| 24. rostralanteriorcingulate.Left | 0.914 | 43.864 |
| 25. caudalanteriorcingulate.Right | 0.020 | 0.952 |
| 26. caudalanteriorcingulate.Left | 0.754 | 36.196 |
| 27. posteriorcingulate.Right | 0.628 | 30.158 |
| 28. posteriorcingulate.Left | 0.878 | 42.133 |
| 29. isthmuscingulate.Right | 0.137 | 6.582 |
| 30. isthmuscingulate.Left | 0.995 | 47.782 |
| 31. insula.Right | 0.604 | 29.004 |
| 32. insula.Left | 0.711 | 34.139 |
| 33. entorhinal.Right | 0.588 | 28.246 |
| 34. entorhinal.Left | 0.442 | 21.214 |
| 35. parahippocampal.Right | 0.415 | 19.938 |
| 36. parahippocampal.Left | 0.720 | 34.547 |
| 37. temporalpole.Right | 0.192 | 9.223 |
| 38. temporalpole.Left | 0.986 | 47.346 |
| 39. fusiform.Right | 0.661 | 31.727 |
| 40. fusiform.Left | 0.959 | 46.038 |
| 41. Right.Accumbens.area | 0.824 | 39.562 |
| 42. Left.Accumbens.area | 0.456 | 21.869 |
| 43. Right.Amygdala | 0.347 | 16.672 |
| 44. Left.Amygdala | 0.620 | 29.771 |
| 45. Right.Caudate | 0.780 | 37.449 |
| 46. Left.Caudate | 0.661 | 31.727 |
| 47. Right.Hippocampus | 0.573 | 27.497 |
| 48. Left.Hippocampus | 0.789 | 37.869 |

**Table S2.** Clinical presentation of patients classified as Dem NOS. Clinical and instrumental data did not meet the diagnostic criteria for any condition

|  | Clinical presentation | |
| --- | --- | --- |
| Dem NOS 1  Dem NOS 2  Dem NOS 3  Dem NOS 4  Dem NOS 5  Dem NOS 6 |  | Moderate dementia (multidomain), mood disorder, negative AD markers  Moderate dementia (multidomain), behavior disorder, negative AD markers, negative FDG-PET  Mild dementia (memory), negative AD markers  Moderate dementia (multidomain), negative AD markers  Mild dementia (multidomain), cerebellar signs, negative AD markers  Moderate dementia (multidomain), cerebellar signs, negative AD markers |

None of these patients met the diagnostic criteria for specific forms of dementia due to non-specificity of symptoms and lack of sufficient follow-up, or due to negativity of the available biomarkers

**Table S3**. Mean and standard deviation (SD) of cortical thickness (mm) and volume (mm^3^) of ROI in AD, non-AD and MCI patients.

| **Region of Interest** | **AD (n=41)** | | **non-AD (n=16)** | | **MCI (n=27)** | |
| --- | --- | --- | --- | --- | --- | --- |
|  | **Left** | **Right** | **Left** | **Right** | **Left** | **Right** |
| Superior frontal gyrus | 2.45 (0.19) | 2.46 (0.17) | 2.48 (0.14) | 2.47 (0.14) | 2.51 (0.15) | 2.50 (0.15) |
| Caudal middle frontal gyrus | 2.32 (0.19) | 2.29 (0.22) | 2.32 (0.16) | 2.37 (0.20) | 2.36 (0.16) | 2.37 (0.17) |
| Rostral middle frontal gyrus | 2.22 (0.19) | 2.22 (0.20) | 2.24 (0.15) | 2.19 (0.17) | 2.23 (0.13) | 2.20 (0.13) |
| Pars opercularis | 2.38 (0.18) | 2.41 (0.16) | 2.38 (0.17) | 2.41 (0.19) | 2.40 (0.14) | 2.43 (0.20) |
| Pars orbitalis | 2.59 (0.26) | 2.59 (0.26) | 2.47 (0.15) | 2.48 (0.22) | 2.60 (0.22) | 2.56 (0.19) |
| Pars triangularis | 2.32 (0.20) | 2.31 (0.17) | 2.25 (0.12) | 2.27 (0.17) | 2.26 (0.14) | 2.27 (0.15) |
| Lateral orbitofrontal cortex | 2.50 (0.16) | 2.50 (0.14) | 2.47 (0.17) | 2.50 (0.14) | 2.55 (0.13) | 2.53 (0.16) |
| Medial orbitofrontal cortex | 2.29 (0.16) | 2.36 (0.18) | 2.32 (0.20) | 2.31 (0.14) | 2.34 (0.14) | 2.39 (0.15) |
| Frontal pole | 2.65 (0.31) | 2.59 (0.27) | 2.61 (0.32) | 2.59 (0.32) | 2.63 (0.30) | 2.61 (0.34) |
| Precentral gyrus | 2.35 (0.21) | 2.31 (0.22) | 2.39 (0.20) | 2.34 (0.22) | 2.38 (0.15) | 2.36 (0.13) |
| Paracentral lobule | 2.21 (0.30) | 2.32 (0.17) | 2.30 (0.14) | 2.32 (0.15) | 2.2 (0.13) | 2.37 (0.16) |
| Cingulate cortex Rostral anterior divison | 2.70 (0.25) | 2.78 (0.29) | 2.61 (0.30) | 2.72 (0.16) | 2.72 (0.25) | 2.81 (0.24) |
| Cingulate cortex Caudal anterior divison | 2.67 (0.24) | 2.53 (0.29) | 2.50 (0.38) | 2.47 (0.31) | 2.69 (0.33) | 2.42 (0.24) |
| Cingulate cortex Posterior divison | 2.29 (0.21) | 2.35 (0.16) | 2.36 (0.15) | 2.37 (0.16) | 2.39 (0.18) | 2.34 (0.18) |
| Cingulate cortex Isthmus divison | 2.17 (0.17) | 2.22 (0.22) | 2.20 (0.15) | 2.29 (0.24) | 2.24 (0.18) | 2.21 (0.15) |
| Insula | 2.71 (0.17) | 2.68 (0.22) | 2.68 (0.23) | 2.72 (0.22) | 2.78 (0.16) | 2.80 (0.21) |
| Entorhinal cortex | 2.84 (0.44) | 2.92 (0.62) | 2.73 (0.66) | 3.03 (0.60) | 3.16 (0.44) | 3.36 (0.52) |
| Parahippocampal gyrus | 2.55 (0.30) | 2.42 (0.33) | 2.51 (0.33) | 2.65 (0.21) | 2.69 (0.43) | 2.66 (0.29) |
| Temporal pole | 3.28 (0.38) | 3.32 (0.42) | 3.13 (0.53) | 3.30 (0.52) | 3.33 (0.33) | 3.45 (0.37) |
| Fusiform gyrus | 2.51 (0.18) | 2.50 (0.29) | 2.55 (0.20) | 2.64 (0.18) | 2.60 (0.16) | 2.66 (0.16) |
| Accumbens area | 0.0003 (0.0001) | 0.0004 (0.0001) | 0.0003 (0.0001) | 0.0004 (0.0001) | 0.0004 (0.0001) | 0.0004 (0.0001) |
| Amygdala | 0.0011 (0.0002) | 0.0012 (0.0002) | 0.0011 (0.0003) | 0.0014 (0.0003) | 0.0012 (0.0003) | 0.0014 (0.0002) |
| Caudate nucleus | 0.0029 (0.0006) | 0.0031 (0.0005) | 0.0027 (0.0004) | 0.0029 (0.0004) | 0.0029 (0.0003) | 0.0031 (0.0004) |
| Hippocampus | 0.0030 (0.0001) | 0.0031 (0.0005) | 0.0030 (0.0004) | 0.0033 (0.0005) | 0.0031 (0.0004) | 0.0033 (0.0004) |

**Table S4.** Differences in CT of cortical ROIs and V of subcortical ROIs between patients with (total NPI sub-domain score ≥ 1) or without specific NPSs (total NPI sub-domain score = 0).

Delusions

| **ROIs** | **AD** | **Non AD** | **MCI** |
| --- | --- | --- | --- |
| superiorfrontal.Right superiorfrontal.Left | 0.231 0.64 | 0.136 0.295 | 0.375 0.232 |
| caudalmiddlefrontal.Right | 0.818 | 0.233 | 0.125 |
| caudalmiddlefrontal.Left | 0.301 | 0.233 | 0.448 |
| rostralmiddlefrontal.Right | 0.659 | 0.07 | 0.576 |
| rostralmiddlefrontal.Left | 0.892 | 0.233 | 0.448 |
| parsopercularis.Right | 0.121 | 0.101 | 0.207 |
| parsopercularis.Left | 0.379 | 0.136 | 0.336 |
| parsorbitalis.Right | 0.056 | 0.295 | 0.759 |
| parsorbitalis.Left | 0.512 | 0.365 | 0.232 |
| parstriangularis.Right | 0.167 | 0.071 | 0.869 |
| parstriangularis.Left | 0.904 | 0.097 | 0.973 |
| lateralorbitofrontal.Right | 0.327 | 0.036 *** | 0.322 |
| lateralorbitofrontal.Left | 0.035 *** | 0.07 | 0.448 |
| medialorbitofrontal.Right | 0.142 | 0.048 *** | 0.869 |
| medialorbitofrontal.Left | 0.64 | 0.945 | 0.183 |
| frontalpole.Right | 0.718 | 0.051 | 0.195 |
| frontalpole.Left | 0.231 | 0.734 | 0.095 |
| precentral.Right | 0.715 | 0.536 | 0.413 |
| precentral.Left | 0.947 | 0.18 | 0.357 |
| paracentral.Right | 0.507 | 0.84 | 0.707 |
| paracentral.Left | 0.561 | 0.945 | 0.539 |
| rostralanteriorcingulate.Right | 0.369 | 1 | 0.448 |
| rostralanteriorcingulate.Left | 0.738 | 0.18 | 1.000 |
| caudalanteriorcingulate.Right | 0.989 | 0.84 | 0.142 |
| caudalanteriorcingulate.Left | 0.871 | 0.84 | 0.432 |
| posteriorcingulate.Right | 0.685 | 0.248 | 0.718 |
| posteriorcingulate.Left | 0.394 | 0.734 | 0.669 |
| isthmuscingulate.Right | 0.495 | 0.536 | 0.372 |
| isthmuscingulate.Left | 0.925 | 0.84 | 0.918 |
| insula.Right | 0.776 | 0.101 | 0.818 |
| insula.Left | 0.33 | 0.295 | 0.669 |
| entorhinal.Right | 0.665 | 1 | 0.869 |
| entorhinal.Left | 0.046 *** | 0.945 | 0.869 |
| parahippocampal.Right | 0.758 | 0.633 | 0.517 |
| parahippocampal.Left | 0.925 | 0.295 | 0.372 |
| temporalpole.Right | 0.698 | 0.448 | 0.669 |
| temporalpole.Left | 0.626 | 0.734 | 0.669 |
| fusiform.Right | 0.718 | 0.101 | 0.448 |
| fusiform.Left | 0.234 | 0.295 | 0.669 |
| Right.Accumbens.area | 0.799 | 0.07 | 0.489 |
| Left.Accumbens.area | 0.478 | 0.84 | 0.336 |
| Right.Amygdala | 0.398 | 0.536 | 0.767 |
| Left.Amygdala | 0.738 | 0.84 | 0.974 |
| Right.Caudate | 0.621 | 0.84 | 0.576 |
| Left.Caudate | 0.547 | 0.448 | 0.921 |
| Right.Hippocampus | 0.445 | 0.048 *** | 0.531 |
| Left.Hippocampus | 0.301 | 0.448 | 0.576 |

Hallucinations

| **ROIs** | **AD** | **Non AD** | **MCI** |
| --- | --- | --- | --- |
| superiorfrontal.Right superiorfrontal.Left | 0.237 0.653 | 0.150 0.333 | not calculable  not calculable |
| caudalmiddlefrontal.Right | 0.977 | 0.600 | not calculable |
| caudalmiddlefrontal.Left | 0.315 | 0.267 | not calculable |
| rostralmiddlefrontal.Right | 0.717 | 0.267 | not calculable |
| rostralmiddlefrontal.Left | 0.251 | 0.333 | not calculable |
| parsopercularis.Right | 0.131 | 0.067 | not calculable |
| parsopercularis.Left | 0.596 | 0.333 | not calculable |
| parsorbitalis.Right | 0.193 | 0.817 | not calculable |
| parsorbitalis.Left | 0.783 | 0.933 | not calculable |
| parstriangularis.Right | 0.176 | 0.067 | not calculable |
| parstriangularis.Left | 0.287 | 0.382 | not calculable |
| lateralorbitofrontal.Right | 0.695 | 0.131 | not calculable |
| lateralorbitofrontal.Left | 0.183 | 0.267 | not calculable |
| medialorbitofrontal.Right | 0.301 | 0.267 | not calculable |
| medialorbitofrontal.Left | 0.988 | 0.933 | not calculable |
| frontalpole.Right | 0.495 | 0.177 | not calculable |
| frontalpole.Left | 0.965 | 0.700 | not calculable |
| precentral.Right | 0.791 | 1.000 | not calculable |
| precentral.Left | 0.695 | 0.417 | not calculable |
| paracentral.Right | 0.735 | 0.817 | not calculable |
| paracentral.Left | 0.965 | 0.417 | not calculable |
| rostralanteriorcingulate.Right | 0.896 | 0.417 | not calculable |
| rostralanteriorcingulate.Left | 0.988 | 0.417 | not calculable |
| caudalanteriorcingulate.Right | 0.566 | 0.150 | not calculable |
| caudalanteriorcingulate.Left | 0.537 | 0.200 | not calculable |
| posteriorcingulate.Right | 0.860 | 0.382 | not calculable |
| posteriorcingulate.Left | 0.702 | 0.267 | not calculable |
| isthmuscingulate.Right | 0.761 | 0.417 | not calculable |
| isthmuscingulate.Left | 0.653 | 0.417 | not calculable |
| insula.Right | 0.895 | 0.333 | not calculable |
| insula.Left | 0.586 | 0.600 | not calculable |
| entorhinal.Right | 0.627 | 0.817 | not calculable |
| entorhinal.Left | 0.329 | 0.700 | not calculable |
| parahippocampal.Right | 0.532 | 0.933 | not calculable |
| parahippocampal.Left | 0.441 | 0.817 | not calculable |
| temporalpole.Right | 0.695 | 0.417 | not calculable |
| temporalpole.Left | 0.332 | 0.417 | not calculable |
| fusiform.Right | 0.873 | 0.150 | not calculable |
| fusiform.Left | 0.941 | 0.500 | not calculable |
| Right.Accumbens.area | 0.441 | 0.267 | not calculable |
| Left.Accumbens.area | 0.942 | 0.500 | not calculable |
| Right.Amygdala | 0.329 | 0.333 | not calculable |
| Left.Amygdala | 0.632 | 0.700 | not calculable |
| Right.Caudate | 0.828 | 0.417 | not calculable |
| Left.Caudate | 0.942 | 0.817 | not calculable |
| Right.Hippocampus | 0.761 | 0.267 | not calculable |
| Left.Hippocampus | 0.193 | 1.000 | not calculable |

Agitation

| **ROIs** | **AD** | **Non AD** | **MCI** |
| --- | --- | --- | --- |
| superiorfrontal.Right superiorfrontal.Left | 0.146 0.886 | 0.878 0.878 | 0.675 0.786 |
| caudalmiddlefrontal.Right | 0.52 | 0.574 | 0.767 |
| caudalmiddlefrontal.Left | 0.458 | 0.234 | 0.865 |
| rostralmiddlefrontal.Right | 0.355 | 0.328 | 0.089 |
| rostralmiddlefrontal.Left | 0.783 | 1.000 | 0.162 |
| parsopercularis.Right | 0.291 | 0.878 | 0.748 |
| parsopercularis.Left | 0.906 | 0.505 | 0.645 |
| parsorbitalis.Right | 0.024 *** | 0.878 | 0.921 |
| parsorbitalis.Left | 0.05 | 0.645 | 0.863 |
| parstriangularis.Right | 0.046 *** | 0.247 | 0.577 |
| parstriangularis.Left | 0.154 | 0.834 | 0.622 |
| lateralorbitofrontal.Right | 0.101 | 0.636 | 0.554 |
| lateralorbitofrontal.Left | 0.474 | 0.645 | 0.904 |
| medialorbitofrontal.Right | 0.427 | 0.798 | 0.753 |
| medialorbitofrontal.Left | 0.328 | 0.328 | 0.767 |
| frontalpole.Right | 0.139 | 0.528 | 0.622 |
| frontalpole.Left | 0.107 | 0.959 | 0.904 |
| precentral.Right | 0.299 | 0.798 | 0.474 |
| precentral.Left | 0.886 | 0.959 | 0.622 |
| paracentral.Right | 0.446 | 1.000 | 0.289 |
| paracentral.Left | 0.431 | 0.574 | 0.505 |
| rostralanteriorcingulate.Right | 0.765 | 0.959 | 0.716 |
| rostralanteriorcingulate.Left | 0.119 | 0.442 | 1 |
| caudalanteriorcingulate.Right | 0.372 | 0.382 | 0.374 |
| caudalanteriorcingulate.Left | 0.958 | 1.000 | 0.183 |
| posteriorcingulate.Right | 0.563 | 0.916 | 0.099 |
| posteriorcingulate.Left | 0.948 | 1.000 | 0.178 |
| isthmuscingulate.Right | 0.245 | 0.442 | 0.008 *** |
| isthmuscingulate.Left | 0.706 | 0.105 | 0.089 |
| insula.Right | 0.026 *** | 0.959 | 0.272 |
| insula.Left | 0.062 | 0.505 | 0.753 |
| entorhinal.Right | 0.044 *** | 0.505 | 0.577 |
| entorhinal.Left | 0.009 *** | 1.000 | 0.148 |
| parahippocampal.Right | 0.205 | 0.442 | 0.474 |
| parahippocampal.Left | 0.031 *** | 0.959 | 0.08 |
| temporalpole.Right | 0.006 *** | 0.959 | 0.827 |
| temporalpole.Left | 0.024 *** | 0.328 | 0.577 |
| fusiform.Right | 0.076 | 0.574 | 0.645 |
| fusiform.Left | 0.025 *** | 0.645 | 0.577 |
| Right.Accumbens.area | 0.458 | 0.878 | 0.272 |
| Left.Accumbens.area | 0.458 | 0.798 | 0.039 *** |
| Right.Amygdala | 0.029 *** | 0.721 | 0.481 |
| Left.Amygdala | 0.139 | 1.000 | 0.544 |
| Right.Caudate | 0.027 *** | 0.130 | 0.753 |
| Left.Caudate | 0.014 *** | 0.721 | 0.753 |
| Right.Hippocampus | 0.225 | 0.721 | 0.981 |
| Left.Hippocampus | 0.886 | 0.505 | 0.318 |

Depression

| **ROIs** | **AD** | **Non AD** | **MCI** |
| --- | --- | --- | --- |
| superiorfrontal.Right superiorfrontal.Left | 0.989 0.583 | 0.071  0.031 *** | 0.150 0.222 |
| caudalmiddlefrontal.Right | 0.892 | 0.055 | 0.204 |
| caudalmiddlefrontal.Left | 0.718 | 0.002 *** | 0.399 |
| rostralmiddlefrontal.Right | 0.904 | 0.606 | 0.683 |
| rostralmiddlefrontal.Left | 0.665 | 0.142 | 0.683 |
| parsopercularis.Right | 0.547 | 0.252 | 0.449 |
| parsopercularis.Left | 0.914 | 0.012 *** | 0.126 |
| parsorbitalis.Right | 0.063 | 0.299 | 0.222 |
| parsorbitalis.Left | 0.277 | 0.071 | 0.449 |
| parstriangularis.Right | 0.432 | 0.368 | 0.486 |
| parstriangularis.Left | 0.698 | 0.112 | 0.807 |
| lateralorbitofrontal.Right | 0.799 | 0.125 | 0.479 |
| lateralorbitofrontal.Left | 0.142 | 0.351 | 0.456 |
| medialorbitofrontal.Right | 0.277 | 0.351 | 0.943 |
| medialorbitofrontal.Left | 0.211 | 0.918 | 0.625 |
| frontalpole.Right | 0.369 | 1 | 0.770 |
| frontalpole.Left | 0.678 | 0.47 | 0.456 |
| precentral.Right | 0.208 | 0.758 | 0.922 |
| precentral.Left | 0.314 | 0.408 | 0.981 |
| paracentral.Right | 0.148 | 0.47 | 0.317 |
| paracentral.Left | 0.068 | 0.21 | 0.341 |
| rostralanteriorcingulate.Right | 0.201 | 0.21 | 0.648 |
| rostralanteriorcingulate.Left | 0.758 | 0.142 | 0.905 |
| caudalanteriorcingulate.Right | 0.665 | 1 | 0.591 |
| caudalanteriorcingulate.Left | 0.561 | 0.012 *** | 0.575 |
| posteriorcingulate.Right | 0.140 | 0.634 | 0.167 |
| posteriorcingulate.Left | 0.425 | 0.055 | 0.300 |
| isthmuscingulate.Right | 0.659 | 0.681 | 0.323 |
| isthmuscingulate.Left | 0.174 | 0.023 *** | 0.479 |
| insula.Right | 0.285 | 0.47 | 0.905 |
| insula.Left | 0.228 | 0.299 | 0.548 |
| entorhinal.Right | 0.695 | 0.536 | 0.829 |
| entorhinal.Left | 0.301 | 0.606 | 0.719 |
| parahippocampal.Right | 0.277 | 0.299 | 0.826 |
| parahippocampal.Left | 0.738 | 0.408 | 0.829 |
| temporalpole.Right | 0.174 | 0.536 | 0.581 |
| temporalpole.Left | 0.343 | 0.758 | 0.943 |
| fusiform.Right | 0.904 | 0.21 | 0.347 |
| fusiform.Left | 0.120 | 0.091 | 0.256 |
| Right.Accumbens.area | 0.121 | 0.758 | 0.236 |
| Left.Accumbens.area | 0.862 | 0.918 | 0.103 |
| Right.Amygdala | 0.947 | 0.758 | 0.399 |
| Left.Amygdala | 0.192 | 1 | 0.648 |
| Right.Caudate | 0.314 | 0.023 *** | 0.683 |
| Left.Caudate | 0.461 | 0.47 | 0.516 |
| Right.Hippocampus | 0.947 | 0.681 | 0.139 |
| Left.Hippocampus | 0.862 | 0.681 | 0.792 |

Anxiety

| **ROIs** | **AD** | **Non AD** | **MCI** |
| --- | --- | --- | --- |
| superiorfrontal.Right superiorfrontal.Left | 0.799 0.602 | 0.583 0.827 | 0.213 0.354 |
| caudalmiddlefrontal.Right | 0.925 | 0.913 | 0.38 |
| caudalmiddlefrontal.Left | 0.678 | 0.827 | 0.581 |
| rostralmiddlefrontal.Right | 0.445 | 0.661 | 0.236 |
| rostralmiddlefrontal.Left | 0.440 | 0.583 | 0.373 |
| parsopercularis.Right | 0.718 | 0.913 | 0.232 |
| parsopercularis.Left | 0.850 | 1.000 | 0.347 |
| parsorbitalis.Right | 0.265 | 1.000 | 0.143 |
| parsorbitalis.Left | 0.738 | 0.583 | 0.77 |
| parstriangularis.Right | 0.379 | 0.140 | 0.126 |
| parstriangularis.Left | 0.565 | 0.692 | 0.66 |
| lateralorbitofrontal.Right | 1.000 | 0.281 | 0.51 |
| lateralorbitofrontal.Left | 1.000 | 0.583 | 0.755 |
| medialorbitofrontal.Right | 0.547 | 1.000 | 0.427 |
| medialorbitofrontal.Left | 0.698 | 0.743 | 0.678 |
| frontalpole.Right | 0.265 | 0.156 | 0.558 |
| frontalpole.Left | 0.529 | 0.583 | 0.217 |
| precentral.Right | 0.617 | 0.441 | 0.643 |
| precentral.Left | 0.758 | 0.743 | 0.788 |
| paracentral.Right | 0.507 | 1.000 | 0.626 |
| paracentral.Left | 0.070 | 1.000 | 0.393 |
| rostralanteriorcingulate.Right | 0.698 | 0.320 | 0.277 |
| rostralanteriorcingulate.Left | 0.211 | 0.743 | 0.014 *** |
| caudalanteriorcingulate.Right | 0.935 | 0.743 | 0.329 |
| caudalanteriorcingulate.Left | 0.968 | 0.180 | 0.241 |
| posteriorcingulate.Right | 0.516 | 0.821 | 0.047 *** |
| posteriorcingulate.Left | 0.797 | 0.913 | 0.2 |
| isthmuscingulate.Right | 0.072 | 0.743 | 0.905 |
| isthmuscingulate.Left | 0.157 | 1.000 | 0.118 |
| insula.Right | 0.617 | 0.827 | 0.943 |
| insula.Left | 0.473 | 0.661 | 0.867 |
| entorhinal.Right | 0.957 | 0.320 | 0.093 |
| entorhinal.Left | 0.925 | 0.441 | 0.516 |
| parahippocampal.Right | 0.414 | 0.510 | 0.884 |
| parahippocampal.Left | 0.640 | 0.377 | 1 |
| temporalpole.Right | 0.738 | 0.221 | 0.059 |
| temporalpole.Left | 0.543 | 0.913 | 0.943 |
| fusiform.Right | 0.718 | 0.827 | 0.792 |
| fusiform.Left | 0.675 | 0.583 | 0.373 |
| Right.Accumbens.area | 0.341 | 0.180 | 0.581 |
| Left.Accumbens.area | 0.758 | 0.661 | 0.581 |
| Right.Amygdala | 0.718 | 0.913 | 0.905 |
| Left.Amygdala | 0.841 | 0.320 | 0.516 |
| Right.Caudate | 0.698 | 0.827 | 0.943 |
| Left.Caudate | 0.602 | 0.583 | 0.399 |
| Right.Hippocampus | 0.659 | 0.441 | 0.581 |
| Left.Hippocampus | 0.341 | 0.913 | 0.683 |

Euphoria

| **ROIs** | **AD** | **Non AD** | **MCI** |
| --- | --- | --- | --- |
| superiorfrontal.Right superiorfrontal.Left | 0.284 0.061 | 0.521 0.8 | 0.318 0.111 |
| caudalmiddlefrontal.Right | 0.645 | 0.364 | 0.086 |
| caudalmiddlefrontal.Left | 0.268 | 0.611 | 0.377 |
| rostralmiddlefrontal.Right | 0.196 | 0.611 | 0.606 |
| rostralmiddlefrontal.Left | 0.037 *** | 0.704 | 0.113 |
| parsopercularis.Right | 0.102 | 0.9 | 0.126 |
| parsopercularis.Left | 0.074 | 0.8 | 0.606 |
| parsorbitalis.Right | 0.128 | 0.364 | 0.18 |
| parsorbitalis.Left | 0.416 | 0.9 | 0.036 *** |
| parstriangularis.Right | 0.754 | 0.459 | 0.88 |
| parstriangularis.Left | 0.552 | 0.459 | 0.473 |
| lateralorbitofrontal.Right | 0.787 | 0.893 | 0.617 |
| lateralorbitofrontal.Left | 0.004 *** | 0.439 | 0.086 |
| medialorbitofrontal.Right | 0.416 | 0.704 | 0.129 |
| medialorbitofrontal.Left | 0.138 | 0.364 | 0.057 |
| frontalpole.Right | 0.319 | 0.281 | 0.596 |
| frontalpole.Left | 0.051 | 0.9 | 0.113 |
| precentral.Right | 0.507 | 1 | 0.19 |
| precentral.Left | 0.602 | 0.9 | 0.086 |
| paracentral.Right | 0.386 | 0.189 | 0.289 |
| paracentral.Left | 0.135 | 1 | 0.105 |
| rostralanteriorcingulate.Right | 0.843 | 0.364 | 0.257 |
| rostralanteriorcingulate.Left | 0.843 | 0.611 | 0.65 |
| caudalanteriorcingulate.Right | 0.839 | 0.521 | 0.685 |
| caudalanteriorcingulate.Left | 0.927 | 0.8 | 0.975 |
| posteriorcingulate.Right | 0.897 | 0.178 | 0.786 |
| posteriorcingulate.Left | 0.941 | 0.8 | 0.411 |
| isthmuscingulate.Right | 0.268 | 0.364 | 0.832 |
| isthmuscingulate.Left | 0.416 | 0.364 | 0.925 |
| insula.Right | 0.053 | 0.8 | 0.976 |
| insula.Left | 0.041 *** | 0.521 | 0.564 |
| entorhinal.Right | 0.036 *** | 0.111 | 0.064 |
| entorhinal.Left | 0.087 | 0.111 | 0.099 |
| parahippocampal.Right | 0.138 | 0.521 | 0.111 |
| parahippocampal.Left | 0.08 | 0.9 | 0.019 *** |
| temporalpole.Right | 0.159 | 0.189 | 0.146 |
| temporalpole.Left | 0.567 | 0.296 | 0.832 |
| fusiform.Right | 0.061 | 0.439 | 0.786 |
| fusiform.Left | 0.293 | 0.611 | 0.88 |
| Right.Accumbens.area | 0.319 | 1 | 0.564 |
| Left.Accumbens.area | 0.438 | 0.057 | 0.928 |
| Right.Amygdala | 0.006 *** | 0.9 | 0.88 |
| Left.Amygdala | 0.268 | 0.039 *** | 0.564 |
| Right.Caudate | 0.733 | 0.439 | 0.928 |
| Left.Caudate | 0.268 | 0.8 | 0.485 |
| Right.Hippocampus | 0.396 | 0.704 | 0.411 |
| Left.Hippocampus | 0.733 | 0.704 | 0.099 |

Apathy

| **ROIs** | **AD** | **Non AD** | **MCI** |
| --- | --- | --- | --- |
| superiorfrontal.Right superiorfrontal.Left | 0.026 *** 0.305 | 0.32  0.038 *** | 0.193 0.193 |
| caudalmiddlefrontal.Right | 0.111 | 0.115 | 0.268 |
| caudalmiddlefrontal.Left | 0.092 | 0.18 | 0.607 |
| rostralmiddlefrontal.Right | 0.108 | 0.221 | 0.092 |
| rostralmiddlefrontal.Left | 0.272 | 0.027 *** | 0.808 |
| parsopercularis.Right | 0.024 *** | 0.069 | 0.293 |
| parsopercularis.Left | 0.333 | 0.32 | 0.533 |
| parsorbitalis.Right | 0.003 *** | 0.51 | 0.868 |
| parsorbitalis.Left | 0.023 *** | 0.51 | 0.361 |
| parstriangularis.Right | 0.028 *** | 0.533 | 0.533 |
| parstriangularis.Left | 0.092 | 0.041 *** | 1 |
| lateralorbitofrontal.Right | 0.069 | 0.141 | 0.782 |
| lateralorbitofrontal.Left | 0.103 | 0.221 | 0.145 |
| medialorbitofrontal.Right | 0.509 | 0.377 | 0.263 |
| medialorbitofrontal.Left | 0.198 | 0.013 *** | 0.293 |
| frontalpole.Right | 0.429 | 1 | 0.115 |
| frontalpole.Left | 0.344 | 1 | 0.4 |
| precentral.Right | 0.133 | 0.583 | 0.391 |
| precentral.Left | 0.578 | 0.441 | 0.268 |
| paracentral.Right | 0.367 | 0.827 | 0.407 |
| paracentral.Left | 0.505 | 0.827 | 0.158 |
| rostralanteriorcingulate.Right | 0.99 | 0.913 | 0.464 |
| rostralanteriorcingulate.Left | 0.928 | 0.221 | 0.145 |
| caudalanteriorcingulate.Right | 0.154 | 0.51 | 0.038 *** |
| caudalanteriorcingulate.Left | 0.117 | 0.441 | 0.455 |
| posteriorcingulate.Right | 0.425 | 0.91 | 0.725 |
| posteriorcingulate.Left | 0.61 | 0.221 | 0.978 |
| isthmuscingulate.Right | 0.344 | 0.51 | 0.607 |
| isthmuscingulate.Left | 0.543 | 0.441 | 0.719 |
| insula.Right | 0.488 | 0.038 *** | 0.464 |
| insula.Left | 0.214 | 0.038 *** | 0.893 |
| entorhinal.Right | 0.513 | 0.221 | 0.85 |
| entorhinal.Left | 0.4 | 0.661 | 0.37 |
| parahippocampal.Right | 0.198 | 0.441 | 0.543 |
| parahippocampal.Left | 0.061 | 0.441 | 0.85 |
| temporalpole.Right | 0.614 | 0.743 | 0.37 |
| temporalpole.Left | 0.886 | 0.661 | 0.464 |
| fusiform.Right | 0.969 | 0.51 | 0.37 |
| fusiform.Left | 0.638 | 1 | 0.57 |
| Right.Accumbens.area | 0.509 | 0.267 | 0.498 |
| Left.Accumbens.area | 0.867 | 1 | 0.935 |
| Right.Amygdala | 0.493 | 0.221 | 0.116 |
| Left.Amygdala | 0.189 | 0.377 | 0.104 |
| Right.Caudate | 0.806 | 0.441 | 0.766 |
| Left.Caudate | 0.928 | 0.913 | 0.607 |
| Right.Hippocampus | 0.907 | 0.052 | 0.072 |
| Left.Hippocampus | 0.969 | 0.377 | 0.145 |

Dishinibition

| **ROIs** | **AD** | **Non AD** | **MCI** |
| --- | --- | --- | --- |
| superiorfrontal.Right superiorfrontal.Left | 0.195 0.436 | 0.758 0.758 | 0.803 0.731 |
| caudalmiddlefrontal.Right | 0.83 | 0.681 | 0.151 |
| caudalmiddlefrontal.Left | 0.543 | 0.408 | 0.284 |
| rostralmiddlefrontal.Right | 0.81 | 0.758 | 0.832 |
| rostralmiddlefrontal.Left | 0.431 | 0.918 | 0.786 |
| parsopercularis.Right | 0.641 | 0.837 | 0.435 |
| parsopercularis.Left | 0.83 | 0.408 | 0.694 |
| parsorbitalis.Right | 0.419 | 0.470 | 0.755 |
| parsorbitalis.Left | 0.724 | 0.408 | 0.596 |
| parstriangularis.Right | 0.576 | 0.458 | 0.88 |
| parstriangularis.Left | 0.832 | 0.560 | 0.708 |
| lateralorbitofrontal.Right | 0.562 | 0.958 | 0.779 |
| lateralorbitofrontal.Left | 0.314 | 0.758 | 0.786 |
| medialorbitofrontal.Right | 0.767 | 0.681 | 0.739 |
| medialorbitofrontal.Left | 0.832 | 0.758 | 0.512 |
| frontalpole.Right | 0.506 | 0.314 | 0.212 |
| frontalpole.Left | 0.581 | 0.351 | 0.485 |
| precentral.Right | 0.538 | 0.470 | 0.399 |
| precentral.Left | 0.562 | 0.681 | 0.349 |
| paracentral.Right | 0.989 | 1.000 | 0.126 |
| paracentral.Left | 0.483 | 0.837 | 0.454 |
| rostralanteriorcingulate.Right | 0.921 | 0.408 | 1 |
| rostralanteriorcingulate.Left | 0.877 | 1.000 | 0.564 |
| caudalanteriorcingulate.Right | 0.678 | 0.606 | 0.708 |
| caudalanteriorcingulate.Left | 0.344 | 0.681 | 0.901 |
| posteriorcingulate.Right | 0.367 | 1.000 | 0.606 |
| posteriorcingulate.Left | 0.808 | 0.758 | 0.65 |
| isthmuscingulate.Right | 0.506 | 0.606 | 0.694 |
| isthmuscingulate.Left | 0.641 | 0.606 | 0.142 |
| insula.Right | 0.501 | 0.758 | 0.055 |
| insula.Left | 0.094 | 0.837 | 0.232 |
| entorhinal.Right | 0.688 | 0.536 | 0.485 |
| entorhinal.Left | 1 | 0.536 | 0.377 |
| parahippocampal.Right | 0.641 | 0.351 | 0.596 |
| parahippocampal.Left | 0.944 | 0.142 | 0.047 *** |
| temporalpole.Right | 0.899 | 0.470 | 0.344 |
| temporalpole.Left | 0.688 | 1.000 | 0.928 |
| fusiform.Right | 0.176 | 0.758 | 0.186 |
| fusiform.Left | 0.099 | 0.351 | 0.65 |
| Right.Accumbens.area | 0.011 *** | 0.408 | 0.447 |
| Left.Accumbens.area | 0.238 | 0.536 | 0.524 |
| Right.Amygdala | 0.342 | 0.918 | 0.65 |
| Left.Amygdala | 0.745 | 0.536 | 0.928 |
| Right.Caudate | 0.078 | 0.837 | 0.786 |
| Left.Caudate | 0.094 | 0.536 | 0.377 |
| Right.Hippocampus | 0.944 | 0.351 | 0.447 |
| Left.Hippocampus | 0.287 | 1.000 | 0.832 |

Irritability

| **ROIs** | **AD** | **Non AD** | **MCI** |
| --- | --- | --- | --- |
| superiorfrontal.Right superiorfrontal.Left | 0.908 0.597 | 1.000 1.000 | 0.062 0.12 |
| caudalmiddlefrontal.Right | 0.705 | 0.758 | 0.076 |
| caudalmiddlefrontal.Left | 0.238 | 0.681 | 0.325 |
| rostralmiddlefrontal.Right | 0.615 | 0.351 | 0.029 *** |
| rostralmiddlefrontal.Left | 0.62 | 0.918 | 0.085 |
| parsopercularis.Right | 0.728 | 0.681 | 0.437 |
| parsopercularis.Left | 0.896 | 0.918 | 0.259 |
| parsorbitalis.Right | 0.149 | 0.758 | 0.094 |
| parsorbitalis.Left | 0.494 | 0.918 | 0.234 |
| parstriangularis.Right | 0.896 | 0.314 | 0.488 |
| parstriangularis.Left | 0.767 | 0.958 | 0.482 |
| lateralorbitofrontal.Right | 0.306 | 1.000 | 0.452 |
| lateralorbitofrontal.Left | 0.67 | 0.681 | 1 |
| medialorbitofrontal.Right | 0.847 | 0.142 | 0.72 |
| medialorbitofrontal.Left | 0.294 | 0.606 | 0.452 |
| frontalpole.Right | 0.689 | 0.340 | 0.512 |
| frontalpole.Left | 0.461 | 0.681 | 0.72 |
| precentral.Right | 0.557 | 0.758 | 0.198 |
| precentral.Left | 0.67 | 0.837 | 0.12 |
| paracentral.Right | 0.335 | 1.000 | 0.332 |
| paracentral.Left | 0.62 | 0.681 | 0.152 |
| rostralanteriorcingulate.Right | 0.415 | 0.606 | 0.943 |
| rostralanteriorcingulate.Left | 0.597 | 0.114 | 0.756 |
| caudalanteriorcingulate.Right | 0.322 | 0.606 | 0.903 |
| caudalanteriorcingulate.Left | 0.584 | 0.758 | 0.104 |
| posteriorcingulate.Right | 0.049 *** | 0.958 | 0.685 |
| posteriorcingulate.Left | 0.279 | 0.408 | 0.905 |
| isthmuscingulate.Right | 0.051 | 1.000 | 0.094 |
| isthmuscingulate.Left | 0.128 | 0.918 | 0.528 |
| insula.Right | 1 | 0.758 | 0.83 |
| insula.Left | 0.297 | 0.918 | 0.43 |
| entorhinal.Right | 0.566 | 1.000 | 0.402 |
| entorhinal.Left | 0.19 | 1.000 | 0.756 |
| parahippocampal.Right | 0.306 | 0.408 | 0.56 |
| parahippocampal.Left | 0.527 | 0.606 | 0.943 |
| temporalpole.Right | 0.826 | 0.681 | 0.72 |
| temporalpole.Left | 0.375 | 0.408 | 0.519 |
| fusiform.Right | 0.561 | 0.837 | 0.094 |
| fusiform.Left | 0.106 | 0.681 | 0.155 |
| Right.Accumbens.area | 0.319 | 0.758 | 0.128 |
| Left.Accumbens.area | 0.969 | 0.536 | 0.155 |
| Right.Amygdala | 0.199 | 0.837 | 0.402 |
| Left.Amygdala | 0.358 | 0.758 | 0.756 |
| Right.Caudate | 0.826 | 0.055 | 0.488 |
| Left.Caudate | 0.579 | 0.606 | 0.756 |
| Right.Hippocampus | 0.806 | 0.408 | 0.905 |
| Left.Hippocampus | 0.887 | 0.210 | 0.488 |

Aberrant motor behavior

| **ROIs** | **AD** | **Non AD** | **MCI** |
| --- | --- | --- | --- |
| superiorfrontal.Right superiorfrontal.Left | 0.533 0.609 | 0.057  0.039 *** | 0.142 0.160 |
| caudalmiddlefrontal.Right | 0.944 | 0.057 | 0.070 |
| caudalmiddlefrontal.Left | 0.668 | 0.111 | 0.411 |
| rostralmiddlefrontal.Right | 0.967 | 0.014 *** | 0.113 |
| rostralmiddlefrontal.Left | 0.575 | 0.025 *** | 0.208 |
| parsopercularis.Right | 0.43 | 0.039 *** | 0.142 |
| parsopercularis.Left | 0.585 | 0.014 *** | 0.099 |
| parsorbitalis.Right | 0.298 | 0.111 | 0.119 |
| parsorbitalis.Left | 0.135 | 0.057 | 0.553 |
| parstriangularis.Right | 0.262 | 0.043 *** | 0.485 |
| parstriangularis.Left | 0.75 | 0.037 *** | 0.617 |
| lateralorbitofrontal.Right | 0.967 | 0.011 *** | 0.779 |
| lateralorbitofrontal.Left | 0.413 | 0.004 *** | 0.099 |
| medialorbitofrontal.Right | 0.75 | 0.014 *** | 0.344 |
| medialorbitofrontal.Left | 0.226 | 0.364 | 0.223 |
| frontalpole.Right | 0.195 | 0.226 | 0.275 |
| frontalpole.Left | 0.108 | 0.704 | 0.411 |
| precentral.Right | 0.695 | 0.189 | 0.318 |
| precentral.Left | 0.857 | 0.057 | 0.289 |
| paracentral.Right | 0.32 | 0.9 | 0.553 |
| paracentral.Left | 0.475 | 0.521 | 1.000 |
| rostralanteriorcingulate.Right | 0.609 | 1 | 0.377 |
| rostralanteriorcingulate.Left | 0.398 | 0.025 *** | 0.976 |
| caudalanteriorcingulate.Right | 0.624 | 0.239 | 0.435 |
| caudalanteriorcingulate.Left | 0.933 | 0.364 | 0.731 |
| posteriorcingulate.Right | 0.245 | 0.037 *** | 0.976 |
| posteriorcingulate.Left | 0.377 | 0.521 | 0.694 |
| isthmuscingulate.Right | 0.272 | 0.239 | 0.524 |
| isthmuscingulate.Left | 0.311 | 1 | 0.212 |
| insula.Right | 0.769 | 0.039 *** | 0.739 |
| insula.Left | 0.433 | 0.146 | 0.786 |
| entorhinal.Right | 0.306 | 0.239 | 0.694 |
| entorhinal.Left | 0.015 *** | 0.521 | 0.880 |
| parahippocampal.Right | 0.609 | 0.611 | 0.596 |
| parahippocampal.Left | 0.186 | 0.611 | 0.524 |
| temporalpole.Right | 0.298 | 1 | 0.447 |
| temporalpole.Left | 0.033 *** | 0.704 | 0.832 |
| fusiform.Right | 0.814 | 0.057 | 0.650 |
| fusiform.Left | 0.889 | 0.704 | 0.564 |
| Right.Accumbens.area | 0.497 | 0.014 *** | 0.786 |
| Left.Accumbens.area | 0.75 | 0.146 | 1.000 |
| Right.Amygdala | 0.57 | 0.8 | 0.739 |
| Left.Amygdala | 0.709 | 0.239 | 0.564 |
| Right.Caudate | 0.967 | 1 | 0.485 |
| Left.Caudate | 0.836 | 0.9 | 0.447 |
| Right.Hippocampus | 0.772 | 0.014 *** | 0.880 |
| Left.Hippocampus | 0.463 | 0.611 | 1.000 |

Night-time behaviour disturbances

| **ROIs** | **AD** | **Non AD** | **MCI** |
| --- | --- | --- | --- |
| superiorfrontal.Right superiorfrontal.Left | 0.104 0.055 | 0.267 0.6 | 0.857 0.979 |
| caudalmiddlefrontal.Right | 0.434 | 0.15 | 0.797 |
| caudalmiddlefrontal.Left | 0.294 | 0.333 | 0.495 |
| rostralmiddlefrontal.Right | 0.747 | 0.1 | 0.705 |
| rostralmiddlefrontal.Left | 0.285 | 0.5 | 0.348 |
| parsopercularis.Right | 0.43 | 0.333 | 0.08 |
| parsopercularis.Left | 0.334 | 0.267 | 0.781 |
| parsorbitalis.Right | 0.786 | 0.1 | 0.237 |
| parsorbitalis.Left | 0.826 | 0.417 | 0.316 |
| parstriangularis.Right | 0.602 | 0.177 | 0.375 |
| parstriangularis.Left | 0.949 | 0.095 | 1 |
| lateralorbitofrontal.Right | 0.651 | 0.095 | 0.938 |
| lateralorbitofrontal.Left | 0.708 | 0.2 | 0.433 |
| medialorbitofrontal.Right | 0.358 | 0.067 | 0.82 |
| medialorbitofrontal.Left | 0.332 | 1 | 0.129 |
| frontalpole.Right | 0.767 | 0.047 *** | 0.837 |
| frontalpole.Left | 0.415 | 0.6 | 0.275 |
| precentral.Right | 0.531 | 0.15 | 0.719 |
| precentral.Left | 0.062 | 0.15 | 0.777 |
| paracentral.Right | 0.047 *** | 0.6 | 0.643 |
| paracentral.Left | 0.328 | 0.7 | 0.471 |
| rostralanteriorcingulate.Right | 0.249 | 1 | 0.085 |
| rostralanteriorcingulate.Left | 0.19 | 0.267 | 0.631 |
| caudalanteriorcingulate.Right | 0.917 | 0.817 | 0.019 *** |
| caudalanteriorcingulate.Left | 0.865 | 0.6 | 0.368 |
| posteriorcingulate.Right | 0.845 | 0.525 | 0.076 |
| posteriorcingulate.Left | 0.215 | 0.7 | 0.145 |
| isthmuscingulate.Right | 0.887 | 0.5 | 0.495 |
| isthmuscingulate.Left | 0.728 | 0.933 | 0.898 |
| insula.Right | 0.907 | 0.067 | 0.743 |
| insula.Left | 0.411 | 0.333 | 0.403 |
| entorhinal.Right | 0.434 | 0.7 | 0.596 |
| entorhinal.Left | 0.579 | 0.7 | 0.212 |
| parahippocampal.Right | 0.615 | 0.333 | 0.537 |
| parahippocampal.Left | 0.99 | 0.1 | 0.322 |
| temporalpole.Right | 0.99 | 0.933 | 0.781 |
| temporalpole.Left | 0.531 | 0.5 | 0.98 |
| fusiform.Right | 0.969 | 0.2 | 0.668 |
| fusiform.Left | 0.335 | 0.267 | 0.94 |
| Right.Accumbens.area | 0.306 | 0.1 | 0.403 |
| Left.Accumbens.area | 0.51 | 1 | 0.86 |
| Right.Amygdala | 0.847 | 0.7 | 1 |
| Left.Amygdala | 0.401 | 0.7 | 0.743 |
| Right.Caudate | 0.728 | 0.817 | 0.433 |
| Left.Caudate | 0.633 | 0.6 | 0.046 *** |
| Right.Hippocampus | 0.579 | 0.2 | 0.668 |
| Left.Hippocampus | 0.728 | 0.15 | 0.9 |

Eating abnormalities

| **ROIs** | **AD** | **Non AD** | **MCI** |
| --- | --- | --- | --- |
| superiorfrontal.Right superiorfrontal.Left | 0.670 0.847 | 0.299 0.142 | 0.268 0.336 |
| caudalmiddlefrontal.Right | 0.814 | 1 | 0.268 |
| caudalmiddlefrontal.Left | 0.887 | 1 | 0.755 |
| rostralmiddlefrontal.Right | 0.928 | 0.071 | 0.842 |
| rostralmiddlefrontal.Left | 0.938 | 0.299 | 0.629 |
| parsopercularis.Right | 0.430 | 0.252 | 0.431 |
| parsopercularis.Left | 0.297 | 0.918 | 0.345 |
| parsorbitalis.Right | 0.083 | 1 | 0.180 |
| parsorbitalis.Left | 0.260 | 1 | 0.930 |
| parstriangularis.Right | 0.273 | 0.203 | 0.887 |
| parstriangularis.Left | 0.767 | 0.832 | 0.705 |
| lateralorbitofrontal.Right | 0.767 | 0.244 | 1.000 |
| lateralorbitofrontal.Left | 0.579 | 0.299 | 0.216 |
| medialorbitofrontal.Right | 0.332 | 1 | 0.289 |
| medialorbitofrontal.Left | 0.806 | 0.071 | 0.579 |
| frontalpole.Right | 0.651 | 0.711 | 0.366 |
| frontalpole.Left | 0.372 | 0.174 | 0.376 |
| precentral.Right | 0.845 | 0.837 | 0.683 |
| precentral.Left | 0.908 | 0.606 | 0.484 |
| paracentral.Right | 0.667 | 0.681 | 0.521 |
| paracentral.Left | 0.473 | 0.606 | 0.641 |
| rostralanteriorcingulate.Right | 0.786 | 0.055 | 0.476 |
| rostralanteriorcingulate.Left | 0.786 | 0.299 | 0.670 |
| caudalanteriorcingulate.Right | 0.481 | 0.47 | 0.861 |
| caudalanteriorcingulate.Left | 0.855 | 0.174 | 0.398 |
| posteriorcingulate.Right | 0.979 | 0.832 | 0.887 |
| posteriorcingulate.Left | 0.845 | 0.252 | 0.670 |
| isthmuscingulate.Right | 0.430 | 0.681 | 0.887 |
| isthmuscingulate.Left | 0.294 | 1 | 0.560 |
| insula.Right | 0.735 | 0.606 | 0.798 |
| insula.Left | 0.593 | 0.252 | 0.476 |
| entorhinal.Right | 0.498 | 1 | 0.075 |
| entorhinal.Left | 0.238 | 0.606 | 0.316 |
| parahippocampal.Right | 0.990 | 0.042 *** | 0.122 |
| parahippocampal.Left | 0.806 | 0.536 | 0.712 |
| temporalpole.Right | 0.949 | 0.536 | 0.075 |
| temporalpole.Left | 0.876 | 0.918 | 0.712 |
| fusiform.Right | 0.386 | 1 | 0.977 |
| fusiform.Left | 0.794 | 0.758 | 0.842 |
| Right.Accumbens.area | 0.294 | 0.758 | 0.376 |
| Left.Accumbens.area | 0.908 | 0.837 | 0.057 |
| Right.Amygdala | 0.806 | 0.758 | 0.550 |
| Left.Amygdala | 0.597 | 1 | 0.712 |
| Right.Caudate | 0.806 | 0.606 | 0.345 |
| Left.Caudate | 0.949 | 0.408 | 0.376 |
| Right.Hippocampus | 0.949 | 0.408 | 0.842 |
| Left.Hippocampus | 0.806 | 0.837 | 0.798 |

**Table S5.** Associations between NPI sub-domains scores (numerical variables) and CT of cortical ROIs and V of subcortical ROIs. Coefficients and p-values of the multivariate linear regression adjusted for age and disease duration are shown.

Delusions

|  | AD b | AD p | non.AD b | non.AD p | MCI b | MCI p |
| --- | --- | --- | --- | --- | --- | --- |
| superiorfrontal.Right superiorfrontal.Left | -0.238 -0.071 | 0.16 0.667 | -0.122 -0.234 | 0.670 0.449 | -0.033 -0.052 | 0.885 0.828 |
| caudalmiddlefrontal.Right | -0.111 | 0.504 | 0.186 | 0.555 | -0.162 | 0.511 |
| caudalmiddlefrontal.Left | -0.097 | 0.561 | -0.279 | 0.387 | -0.068 | 0.788 |
| rostralmiddlefrontal.Right | -0.128 | 0.434 | -0.254 | 0.413 | 0.150 | 0.543 |
| rostralmiddlefrontal.Left | -0.114 | 0.483 | -0.237 | 0.413 | 0.139 | 0.554 |
| parsopercularis.Right | -0.252 | 0.122 | -0.184 | 0.539 | 0.302 | 0.172 |
| parsopercularis.Left | -0.114 | 0.483 | -0.039 | 0.904 | 0.070 | 0.778 |
| parsorbitalis.Right | -0.336 | 0.034 *** | 0.075 | 0.811 | 0.191 | 0.397 |
| parsorbitalis.Left | -0.252 | 0.119 | -0.075 | 0.819 | 0.214 | 0.309 |
| parstriangularis.Right | -0.278 | 0.1 | -0.193 | 0.500 | 0.087 | 0.708 |
| parstriangularis.Left | -0.108 | 0.506 | -0.122 | 0.688 | 0.443 | 0.043 *** |
| lateralorbitofrontal.Right | -0.121 | 0.472 | -0.495 | 0.103 | 0.181 | 0.392 |
| lateralorbitofrontal.Left | -0.262 | 0.105 | -0.286 | 0.382 | 0.089 | 0.69 |
| medialorbitofrontal.Right | -0.143 | 0.378 | -0.331 | 0.294 | 0.199 | 0.343 |
| medialorbitofrontal.Left | -0.133 | 0.421 | -0.152 | 0.628 | -0.081 | 0.716 |
| frontalpole.Right | 0.018 | 0.913 | -0.477 | 0.089 | 0.019 | 0.929 |
| frontalpole.Left | 0.026 | 0.878 | -0.206 | 0.515 | 0.069 | 0.747 |
| precentral.Right | -0.074 | 0.68 | 0.199 | 0.536 | -0.172 | 0.476 |
| precentral.Left | -0.007 | 0.97 | 0.067 | 0.846 | -0.083 | 0.747 |
| paracentral.Right | -0.165 | 0.354 | -0.126 | 0.721 | -0.108 | 0.621 |
| paracentral.Left | 0.060 | 0.715 | -0.176 | 0.537 | -0.151 | 0.503 |
| rostralanteriorcingulate.Right | 0.081 | 0.62 | -0.394 | 0.202 | 0.123 | 0.573 |
| rostralanteriorcingulate.Left | -0.127 | 0.432 | -0.283 | 0.394 | 0.259 | 0.211 |
| caudalanteriorcingulate.Right | -0.032 | 0.847 | 0.225 | 0.436 | 0.295 | 0.175 |
| caudalanteriorcingulate.Left | 0.116 | 0.474 | -0.350 | 0.226 | 0.130 | 0.548 |
| posteriorcingulate.Right | -0.072 | 0.666 | -0.080 | 0.834 | 0.157 | 0.472 |
| posteriorcingulate.Left | -0.054 | 0.741 | -0.425 | 0.227 | 0.180 | 0.447 |
| isthmuscingulate.Right | -0.214 | 0.232 | -0.086 | 0.862 | 0.018 | 0.938 |
| isthmuscingulate.Left | -0.004 | 0.98 | -0.217 | 0.543 | -0.096 | 0.692 |
| insula.Right | -0.081 | 0.642 | -0.030 | 0.927 | -0.135 | 0.573 |
| insula.Left | -0.167 | 0.334 | -0.193 | 0.594 | 0.071 | 0.774 |
| entorhinal.Right | -0.159 | 0.364 | 0.250 | 0.483 | -0.096 | 0.671 |
| entorhinal.Left | -0.356 | 0.037 *** | 0.311 | 0.441 | 0.071 | 0.748 |
| parahippocampal.Right | -0.101 | 0.567 | 0.135 | 0.702 | 0.216 | 0.343 |
| parahippocampal.Left | -0.094 | 0.603 | 0.028 | 0.944 | -0.047 | 0.838 |
| temporalpole.Right | -0.231 | 0.192 | 0.512 | 0.130 | 0.072 | 0.745 |
| temporalpole.Left | -0.151 | 0.386 | 0.343 | 0.316 | 0.096 | 0.666 |
| fusiform.Right | -0.163 | 0.355 | -0.377 | 0.314 | -0.016 | 0.948 |
| fusiform.Left | -0.100 | 0.56 | 0.032 | 0.933 | 0.073 | 0.793 |
| Right.Accumbens.area | 0.052 | 0.761 | -0.085 | 0.788 | 0.167 | 0.483 |
| Left.Accumbens.area | -0.064 | 0.711 | 0.313 | 0.309 | 0.224 | 0.315 |
| Right.Amygdala | -0.082 | 0.636 | 0.339 | 0.224 | 0.105 | 0.656 |
| Left.Amygdala | -0.135 | 0.412 | 0.428 | 0.370 | 0.238 | 0.299 |
| Right.Caudate | -0.021 | 0.898 | -0.195 | 0.545 | 0.381 | 0.068 |
| Left.Caudate | -0.064 | 0.694 | -0.166 | 0.637 | 0.311 | 0.143 |
| Right.Hippocampus | -0.086 | 0.649 | -0.305 | 0.383 | 0.493 | 0.039 *** |
| Left.Hippocampus | -0.132 | 0.459 | 0.063 | 0.876 | 0.442 | 0.059 |

Hallucinations

|  | AD b | AD p | non.AD b | non.AD p | MCI b | MCI p |
| --- | --- | --- | --- | --- | --- | --- |
| superiorfrontal.Right superiorfrontal.Left | -0.182 -0.033 | 0.285 0.841 | -0.058 -0.238 | 0.835 0.399 | not calculable not calculable | not calculable not calculable |
| caudalmiddlefrontal.Right | -0.015 | 0.928 | 0.174 | 0.555 | not calculable | not calculable |
| caudalmiddlefrontal.Left | -0.011 | 0.948 | -0.158 | 0.596 | not calculable | not calculable |
| rostralmiddlefrontal.Right | -0.078 | 0.633 | -0.042 | 0.889 | not calculable | not calculable |
| rostralmiddlefrontal.Left | -0.150 | 0.354 | -0.269 | 0.339 | not calculable | not calculable |
| parsopercularis.Right | -0.171 | 0.300 | -0.183 | 0.526 | not calculable | not calculable |
| parsopercularis.Left | -0.031 | 0.848 | -0.033 | 0.915 | not calculable | not calculable |
| parsorbitalis.Right | -0.246 | 0.124 | 0.130 | 0.667 | not calculable | not calculable |
| parsorbitalis.Left | -0.158 | 0.331 | -0.128 | 0.665 | not calculable | not calculable |
| parstriangularis.Right | -0.251 | 0.138 | -0.069 | 0.808 | not calculable | not calculable |
| parstriangularis.Left | -0.192 | 0.233 | 0.009 | 0.975 | not calculable | not calculable |
| lateralorbitofrontal.Right | -0.029 | 0.864 | -0.246 | 0.417 | not calculable | not calculable |
| lateralorbitofrontal.Left | -0.187 | 0.250 | -0.225 | 0.459 | not calculable | not calculable |
| medialorbitofrontal.Right | -0.139 | 0.392 | -0.075 | 0.812 | not calculable | not calculable |
| medialorbitofrontal.Left | -0.086 | 0.604 | -0.095 | 0.755 | not calculable | not calculable |
| frontalpole.Right | -0.126 | 0.454 | -0.080 | 0.780 | not calculable | not calculable |
| frontalpole.Left | -0.094 | 0.573 | -0.055 | 0.862 | not calculable | not calculable |
| precentral.Right | 0.023 | 0.899 | 0.284 | 0.358 | not calculable | not calculable |
| precentral.Left | 0.050 | 0.775 | 0.163 | 0.610 | not calculable | not calculable |
| paracentral.Right | -0.108 | 0.546 | 0.144 | 0.677 | not calculable | not calculable |
| paracentral.Left | 0.016 | 0.921 | -0.244 | 0.376 | not calculable | not calculable |
| rostralanteriorcingulate.Right | 0.071 | 0.664 | -0.060 | 0.851 | not calculable | not calculable |
| rostralanteriorcingulate.Left | -0.049 | 0.763 | -0.079 | 0.797 | not calculable | not calculable |
| caudalanteriorcingulate.Right | -0.107 | 0.512 | 0.216 | 0.444 | not calculable | not calculable |
| caudalanteriorcingulate.Left | 0.036 | 0.824 | -0.331 | 0.229 | not calculable | not calculable |
| posteriorcingulate.Right | -0.011 | 0.948 | -0.306 | 0.317 | not calculable | not calculable |
| posteriorcingulate.Left | 0.043 | 0.795 | -0.459 | 0.079 | not calculable | not calculable |
| isthmuscingulate.Right | -0.054 | 0.764 | -0.098 | 0.751 | not calculable | not calculable |
| isthmuscingulate.Left | -0.074 | 0.654 | -0.160 | 0.582 | not calculable | not calculable |
| insula.Right | 0.057 | 0.745 | 0.106 | 0.731 | not calculable | not calculable |
| insula.Left | -0.106 | 0.542 | -0.006 | 0.985 | not calculable | not calculable |
| entorhinal.Right | -0.004 | 0.982 | 0.270 | 0.376 | not calculable | not calculable |
| entorhinal.Left | -0.323 | 0.059 | 0.277 | 0.377 | not calculable | not calculable |
| parahippocampal.Right | -0.037 | 0.832 | 0.234 | 0.479 | not calculable | not calculable |
| parahippocampal.Left | -0.290 | 0.102 | 0.634 | 0.085 | not calculable | not calculable |
| temporalpole.Right | -0.009 | 0.961 | 0.504 | 0.070 | not calculable | not calculable |
| temporalpole.Left | -0.082 | 0.640 | 0.377 | 0.183 | not calculable | not calculable |
| fusiform.Right | -0.122 | 0.491 | -0.198 | 0.550 | not calculable | not calculable |
| fusiform.Left | -0.053 | 0.757 | 0.193 | 0.541 | not calculable | not calculable |
| Right.Accumbens.area | 0.160 | 0.344 | -0.041 | 0.895 | not calculable | not calculable |
| Left.Accumbens.area | -0.007 | 0.967 | 0.168 | 0.552 | not calculable | not calculable |
| Right.Amygdala | -0.149 | 0.390 | 0.417 | 0.115 | not calculable | not calculable |
| Left.Amygdala | -0.184 | 0.262 | 0.172 | 0.597 | not calculable | not calculable |
| Right.Caudate | -0.072 | 0.658 | -0.172 | 0.557 | not calculable | not calculable |
| Left.Caudate | -0.041 | 0.799 | -0.157 | 0.614 | not calculable | not calculable |
| Right.Hippocampus | 0.006 | 0.976 | -0.357 | 0.268 | not calculable | not calculable |
| Left.Hippocampus | -0.194 | 0.274 | 0.243 | 0.450 | not calculable | not calculable |

Agitation

|  | AD b | AD p | non.AD b | non.AD p | MCI b | MCI p |
| --- | --- | --- | --- | --- | --- | --- |
| superiorfrontal.Right superiorfrontal.Left | -0.336 -0.071 | 0.047 ***  0.673 | 0.022 0.020 | 0.939 0.945 | 0.037 0.068 | 0.867 0.768 |
| caudalmiddlefrontal.Right | -0.358 | 0.028 *** | 0.115 | 0.705 | -0.223 | 0.349 |
| caudalmiddlefrontal.Left | -0.159 | 0.345 | 0.015 | 0.962 | 0.020 | 0.935 |
| rostralmiddlefrontal.Right | -0.267 | 0.101 | -0.378 | 0.209 | 0.216 | 0.361 |
| rostralmiddlefrontal.Left | -0.115 | 0.482 | -0.036 | 0.903 | 0.279 | 0.211 |
| parsopercularis.Right | -0.451 | 0.004 *** | 0.060 | 0.839 | 0.138 | 0.525 |
| parsopercularis.Left | -0.087 | 0.597 | 0.104 | 0.738 | 0.143 | 0.550 |
| parsorbitalis.Right | -0.302 | 0.06 | -0.063 | 0.839 | 0.094 | 0.667 |
| parsorbitalis.Left | -0.298 | 0.066 | -0.029 | 0.924 | 0.092 | 0.652 |
| parstriangularis.Right | -0.457 | 0.006 *** | -0.097 | 0.737 | 0.234 | 0.290 |
| parstriangularis.Left | -0.207 | 0.201 | -0.231 | 0.446 | 0.238 | 0.275 |
| lateralorbitofrontal.Right | -0.263 | 0.117 | -0.493 | 0.097 | 0.033 | 0.873 |
| lateralorbitofrontal.Left | -0.099 | 0.551 | -0.368 | 0.226 | 0.065 | 0.765 |
| medialorbitofrontal.Right | -0.109 | 0.505 | -0.382 | 0.224 | 0.131 | 0.520 |
| medialorbitofrontal.Left | -0.162 | 0.331 | -0.375 | 0.216 | -0.072 | 0.738 |
| frontalpole.Right | 0.034 | 0.842 | -0.503 | 0.065 | 0.170 | 0.401 |
| frontalpole.Left | 0.095 | 0.57 | -0.255 | 0.421 | 0.150 | 0.465 |
| precentral.Right | -0.255 | 0.155 | 0.011 | 0.974 | -0.217 | 0.350 |
| precentral.Left | -0.235 | 0.179 | -0.210 | 0.521 | -0.182 | 0.466 |
| paracentral.Right | -0.182 | 0.31 | -0.182 | 0.605 | -0.325 | 0.116 |
| paracentral.Left | -0.094 | 0.569 | 0.153 | 0.591 | -0.307 | 0.151 |
| rostralanteriorcingulate.Right | 0.153 | 0.35 | -0.120 | 0.709 | -0.103 | 0.626 |
| rostralanteriorcingulate.Left | -0.200 | 0.216 | -0.273 | 0.380 | 0.157 | 0.438 |
| caudalanteriorcingulate.Right | 0.139 | 0.402 | -0.152 | 0.599 | -0.002 | 0.993 |
| caudalanteriorcingulate.Left | -0.003 | 0.986 | 0.027 | 0.926 | -0.047 | 0.821 |
| posteriorcingulate.Right | -0.146 | 0.387 | 0.029 | 0.928 | -0.109 | 0.607 |
| posteriorcingulate.Left | -0.136 | 0.41 | -0.015 | 0.957 | -0.063 | 0.783 |
| isthmuscingulate.Right | -0.028 | 0.876 | -0.072 | 0.820 | -0.349 | 0.104 |
| isthmuscingulate.Left | 0.223 | 0.175 | 0.217 | 0.463 | -0.311 | 0.175 |
| insula.Right | -0.287 | 0.097 | -0.192 | 0.539 | -0.214 | 0.354 |
| insula.Left | -0.280 | 0.105 | -0.412 | 0.180 | -0.092 | 0.703 |
| entorhinal.Right | -0.235 | 0.181 | 0.019 | 0.951 | -0.361 | 0.089 |
| entorhinal.Left | -0.175 | 0.32 | -0.181 | 0.576 | -0.232 | 0.269 |
| parahippocampal.Right | -0.180 | 0.306 | 0.098 | 0.774 | -0.239 | 0.277 |
| parahippocampal.Left | -0.177 | 0.328 | -0.477 | 0.219 | -0.400 | 0.060 |
| temporalpole.Right | -0.395 | 0.023 *** | -0.082 | 0.787 | -0.286 | 0.174 |
| temporalpole.Left | -0.229 | 0.19 | -0.282 | 0.340 | -0.231 | 0.277 |
| fusiform.Right | -0.176 | 0.322 | -0.139 | 0.684 | -0.428 | 0.053 |
| fusiform.Left | -0.112 | 0.516 | -0.238 | 0.460 | -0.210 | 0.429 |
| Right.Accumbens.area | 0.031 | 0.858 | -0.097 | 0.761 | 0.197 | 0.389 |
| Left.Accumbens.area | -0.051 | 0.772 | 0.008 | 0.977 | 0.295 | 0.167 |
| Right.Amygdala | -0.171 | 0.327 | 0.033 | 0.908 | -0.361 | 0.104 |
| Left.Amygdala | -0.059 | 0.724 | -0.173 | 0.604 | -0.152 | 0.496 |
| Right.Caudate | 0.113 | 0.489 | 0.252 | 0.398 | 0.091 | 0.662 |
| Left.Caudate | 0.074 | 0.652 | 0.204 | 0.521 | 0.002 | 0.992 |
| Right.Hippocampus | -0.156 | 0.409 | -0.090 | 0.791 | 0.046 | 0.850 |
| Left.Hippocampus | -0.068 | 0.706 | -0.452 | 0.157 | -0.019 | 0.937 |

Depression

|  | AD b | AD p | non.AD b | non.AD p | MCI b | MCI p |
| --- | --- | --- | --- | --- | --- | --- |
| superiorfrontal.Right superiorfrontal.Left | 0.139 0.192 | 0.425 0.249 | -0.069 -0.330 | 0.805 0.237 | -0.338 -0.312 | 0.094 0.143 |
| caudalmiddlefrontal.Right | 0.066 | 0.695 | 0.126 | 0.672 | -0.448 | 0.039 *** |
| caudalmiddlefrontal.Left | 0.105 | 0.537 | -0.412 | 0.153 | -0.117 | 0.61 |
| rostralmiddlefrontal.Right | 0.063 | 0.704 | -0.045 | 0.883 | -0.155 | 0.49 |
| rostralmiddlefrontal.Left | 0.060 | 0.718 | -0.281 | 0.320 | -0.219 | 0.303 |
| parsopercularis.Right | 0.118 | 0.481 | -0.164 | 0.572 | 0.013 | 0.949 |
| parsopercularis.Left | 0.053 | 0.749 | -0.146 | 0.633 | -0.298 | 0.183 |
| parsorbitalis.Right | -0.228 | 0.163 | 0.103 | 0.736 | -0.092 | 0.658 |
| parsorbitalis.Left | -0.127 | 0.445 | -0.135 | 0.648 | 0.092 | 0.636 |
| parstriangularis.Right | -0.110 | 0.528 | -0.129 | 0.649 | 0.121 | 0.567 |
| parstriangularis.Left | 0.076 | 0.644 | -0.028 | 0.927 | 0.122 | 0.56 |
| lateralorbitofrontal.Right | -0.013 | 0.938 | -0.340 | 0.258 | 0.203 | 0.293 |
| lateralorbitofrontal.Left | 0.078 | 0.640 | -0.122 | 0.692 | -0.061 | 0.767 |
| medialorbitofrontal.Right | -0.084 | 0.612 | 0.021 | 0.948 | -0.057 | 0.77 |
| medialorbitofrontal.Left | 0.022 | 0.898 | -0.103 | 0.737 | -0.279 | 0.161 |
| frontalpole.Right | 0.219 | 0.198 | -0.107 | 0.708 | -0.078 | 0.685 |
| frontalpole.Left | 0.153 | 0.364 | -0.137 | 0.663 | -0.131 | 0.501 |
| precentral.Right | 0.223 | 0.218 | 0.251 | 0.421 | -0.264 | 0.228 |
| precentral.Left | 0.284 | 0.105 | 0.180 | 0.575 | -0.034 | 0.886 |
| paracentral.Right | 0.192 | 0.288 | -0.052 | 0.882 | -0.295 | 0.132 |
| paracentral.Left | 0.236 | 0.150 | -0.204 | 0.464 | -0.446 | 0.023 *** |
| rostralanteriorcingulate.Right | 0.235 | 0.150 | -0.264 | 0.400 | 0.211 | 0.287 |
| rostralanteriorcingulate.Left | -0.004 | 0.980 | -0.255 | 0.404 | -0.017 | 0.929 |
| caudalanteriorcingulate.Right | 0.029 | 0.864 | 0.298 | 0.287 | 0.005 | 0.98 |
| caudalanteriorcingulate.Left | 0.191 | 0.242 | -0.496 | 0.061 | 0.294 | 0.129 |
| posteriorcingulate.Right | 0.309 | 0.064 | -0.156 | 0.619 | 0.243 | 0.219 |
| posteriorcingulate.Left | 0.151 | 0.362 | -0.470 | 0.073 | 0.378 | 0.073 |
| isthmuscingulate.Right | -0.233 | 0.199 | -0.164 | 0.597 | 0.050 | 0.81 |
| isthmuscingulate.Left | -0.109 | 0.516 | -0.344 | 0.226 | 0.047 | 0.832 |
| insula.Right | -0.055 | 0.758 | 0.106 | 0.732 | -0.170 | 0.439 |
| insula.Left | -0.274 | 0.115 | -0.243 | 0.433 | -0.011 | 0.961 |
| entorhinal.Right | -0.237 | 0.180 | 0.051 | 0.871 | -0.302 | 0.136 |
| entorhinal.Left | -0.313 | 0.073 | 0.113 | 0.724 | -0.265 | 0.179 |
| parahippocampal.Right | 0.063 | 0.724 | -0.003 | 0.992 | -0.158 | 0.45 |
| parahippocampal.Left | 0.060 | 0.745 | -0.158 | 0.686 | -0.073 | 0.727 |
| temporalpole.Right | -0.069 | 0.703 | 0.181 | 0.543 | -0.156 | 0.439 |
| temporalpole.Left | 0.188 | 0.288 | 0.179 | 0.543 | -0.241 | 0.231 |
| fusiform.Right | -0.074 | 0.679 | -0.331 | 0.313 | -0.144 | 0.509 |
| fusiform.Left | -0.163 | 0.347 | -0.298 | 0.343 | -0.032 | 0.9 |
| Right.Accumbens.area | 0.163 | 0.345 | 0.075 | 0.813 | 0.174 | 0.422 |
| Left.Accumbens.area | 0.086 | 0.623 | 0.182 | 0.522 | 0.180 | 0.379 |
| Right.Amygdala | -0.020 | 0.909 | 0.366 | 0.174 | -0.070 | 0.745 |
| Left.Amygdala | -0.246 | 0.137 | 0.048 | 0.885 | 0.057 | 0.788 |
| Right.Caudate | 0.146 | 0.373 | -0.364 | 0.204 | -0.080 | 0.686 |
| Left.Caudate | 0.144 | 0.382 | -0.343 | 0.264 | -0.039 | 0.844 |
| Right.Hippocampus | -0.175 | 0.360 | -0.209 | 0.527 | 0.100 | 0.662 |
| Left.Hippocampus | -0.053 | 0.768 | 0.005 | 0.988 | 0.156 | 0.48 |

Anxiety

|  | AD b | AD p | non.AD b | non.AD p | MCI b | MCI p |
| --- | --- | --- | --- | --- | --- | --- |
| superiorfrontal.Right superiorfrontal.Left | -0.016 0.055 | 0.926 0.731 | -0.156 -0.308 | 0.593 0.295 | -0.368 -0.272 | 0.082 0.226 |
| caudalmiddlefrontal.Right | -0.073 | 0.652 | 0.135 | 0.666 | -0.448 | 0.05 |
| caudalmiddlefrontal.Left | -0.030 | 0.854 | -0.266 | 0.392 | -0.290 | 0.223 |
| rostralmiddlefrontal.Right | 0.001 | 0.997 | -0.250 | 0.428 | -0.299 | 0.199 |
| rostralmiddlefrontal.Left | -0.001 | 0.997 | -0.352 | 0.231 | -0.209 | 0.35 |
| parsopercularis.Right | -0.100 | 0.532 | -0.181 | 0.552 | -0.155 | 0.471 |
| parsopercularis.Left | -0.139 | 0.380 | -0.101 | 0.753 | -0.120 | 0.613 |
| parsorbitalis.Right | -0.177 | 0.261 | -0.005 | 0.987 | -0.151 | 0.485 |
| parsorbitalis.Left | -0.225 | 0.153 | -0.168 | 0.586 | 0.146 | 0.47 |
| parstriangularis.Right | -0.208 | 0.209 | -0.204 | 0.488 | -0.205 | 0.353 |
| parstriangularis.Left | -0.035 | 0.827 | -0.196 | 0.531 | 0.068 | 0.756 |
| lateralorbitofrontal.Right | 0.076 | 0.646 | -0.516 | 0.090 | -0.019 | 0.926 |
| lateralorbitofrontal.Left | -0.042 | 0.793 | -0.321 | 0.311 | 0.066 | 0.759 |
| medialorbitofrontal.Right | 0.037 | 0.818 | -0.328 | 0.315 | -0.058 | 0.776 |
| medialorbitofrontal.Left | -0.057 | 0.722 | -0.149 | 0.643 | -0.233 | 0.267 |
| frontalpole.Right | 0.257 | 0.112 | -0.398 | 0.169 | -0.069 | 0.735 |
| frontalpole.Left | 0.029 | 0.857 | -0.192 | 0.559 | 0.021 | 0.917 |
| precentral.Right | -0.016 | 0.925 | 0.221 | 0.500 | -0.383 | 0.09 |
| precentral.Left | -0.043 | 0.802 | 0.076 | 0.823 | -0.062 | 0.802 |
| paracentral.Right | -0.067 | 0.700 | -0.024 | 0.948 | -0.117 | 0.577 |
| paracentral.Left | 0.113 | 0.477 | -0.219 | 0.453 | -0.085 | 0.695 |
| rostralanteriorcingulate.Right | 0.087 | 0.583 | -0.371 | 0.251 | 0.069 | 0.742 |
| rostralanteriorcingulate.Left | -0.148 | 0.345 | -0.341 | 0.282 | -0.409 | 0.033 *** |
| caudalanteriorcingulate.Right | 0.188 | 0.236 | 0.175 | 0.558 | -0.078 | 0.715 |
| caudalanteriorcingulate.Left | -0.092 | 0.558 | -0.430 | 0.131 | -0.161 | 0.437 |
| posteriorcingulate.Right | 0.100 | 0.537 | -0.176 | 0.590 | -0.090 | 0.667 |
| posteriorcingulate.Left | -0.105 | 0.508 | -0.385 | 0.173 | 0.266 | 0.239 |
| isthmuscingulate.Right | -0.171 | 0.328 | -0.141 | 0.665 | -0.028 | 0.898 |
| isthmuscingulate.Left | 0.005 | 0.977 | -0.225 | 0.458 | 0.492 | 0.026 *** |
| insula.Right | -0.109 | 0.520 | -0.026 | 0.935 | -0.070 | 0.764 |
| insula.Left | -0.097 | 0.565 | -0.178 | 0.587 | 0.016 | 0.948 |
| entorhinal.Right | 0.036 | 0.834 | 0.224 | 0.489 | 0.062 | 0.775 |
| entorhinal.Left | -0.065 | 0.702 | 0.266 | 0.421 | 0.012 | 0.954 |
| parahippocampal.Right | -0.032 | 0.851 | 0.070 | 0.843 | -0.021 | 0.923 |
| parahippocampal.Left | -0.044 | 0.803 | 0.237 | 0.563 | 0.020 | 0.927 |
| temporalpole.Right | 0.064 | 0.713 | 0.431 | 0.151 | 0.108 | 0.611 |
| temporalpole.Left | 0.059 | 0.731 | 0.333 | 0.270 | -0.028 | 0.896 |
| fusiform.Right | 0.260 | 0.126 | -0.357 | 0.298 | -0.050 | 0.827 |
| fusiform.Left | 0.146 | 0.379 | 0.051 | 0.880 | 0.268 | 0.308 |
| Right.Accumbens.area | 0.195 | 0.235 | -0.195 | 0.552 | 0.057 | 0.802 |
| Left.Accumbens.area | 0.050 | 0.767 | 0.259 | 0.381 | -0.047 | 0.829 |
| Right.Amygdala | 0.239 | 0.152 | 0.336 | 0.238 | -0.274 | 0.22 |
| Left.Amygdala | 0.149 | 0.351 | 0.154 | 0.654 | -0.203 | 0.358 |
| Right.Caudate | 0.112 | 0.477 | -0.143 | 0.644 | -0.213 | 0.299 |
| Left.Caudate | 0.012 | 0.940 | -0.110 | 0.739 | -0.082 | 0.695 |
| Right.Hippocampus | -0.028 | 0.877 | -0.463 | 0.166 | -0.395 | 0.09 |
| Left.Hippocampus | 0.043 | 0.804 | 0.038 | 0.912 | -0.016 | 0.946 |

Euphoria

|  | AD b | AD p | non.AD b | non.AD p | MCI b | MCI p |
| --- | --- | --- | --- | --- | --- | --- |
| superiorfrontal.Right superiorfrontal.Left | -0.093 -0.128 | 0.596 0.444 | 0.054 0.067 | 0.848 0.816 | -0.244 -0.178 | 0.274 0.45 |
| caudalmiddlefrontal.Right | 0.079 | 0.639 | -0.002 | 0.994 | -0.471 | 0.047 *** |
| caudalmiddlefrontal.Left | -0.007 | 0.968 | -0.046 | 0.878 | -0.111 | 0.657 |
| rostralmiddlefrontal.Right | -0.063 | 0.704 | -0.039 | 0.899 | -0.318 | 0.188 |
| rostralmiddlefrontal.Left | -0.096 | 0.560 | -0.036 | 0.899 | -0.259 | 0.262 |
| parsopercularis.Right | -0.061 | 0.717 | 0.083 | 0.775 | -0.163 | 0.465 |
| parsopercularis.Left | -0.133 | 0.424 | 0.006 | 0.985 | 0.076 | 0.759 |
| parsorbitalis.Right | -0.132 | 0.424 | -0.015 | 0.960 | -0.354 | 0.107 |
| parsorbitalis.Left | 0.019 | 0.911 | 0.124 | 0.675 | -0.262 | 0.208 |
| parstriangularis.Right | 0.056 | 0.749 | 0.072 | 0.798 | -0.124 | 0.59 |
| parstriangularis.Left | -0.026 | 0.876 | -0.074 | 0.805 | -0.079 | 0.729 |
| lateralorbitofrontal.Right | 0.048 | 0.780 | 0.119 | 0.698 | -0.349 | 0.09 |
| lateralorbitofrontal.Left | -0.302 | 0.065 | 0.480 | 0.098 | -0.187 | 0.399 |
| medialorbitofrontal.Right | -0.096 | 0.561 | 0.180 | 0.569 | -0.228 | 0.275 |
| medialorbitofrontal.Left | -0.112 | 0.504 | 0.282 | 0.349 | -0.161 | 0.463 |
| frontalpole.Right | -0.165 | 0.335 | 0.018 | 0.951 | -0.035 | 0.868 |
| frontalpole.Left | -0.310 | 0.061 | -0.174 | 0.578 | -0.106 | 0.619 |
| precentral.Right | 0.239 | 0.186 | 0.089 | 0.777 | -0.269 | 0.26 |
| precentral.Left | 0.244 | 0.167 | 0.271 | 0.394 | -0.250 | 0.329 |
| paracentral.Right | 0.141 | 0.436 | -0.025 | 0.941 | -0.264 | 0.219 |
| paracentral.Left | 0.137 | 0.407 | 0.238 | 0.389 | -0.167 | 0.456 |
| rostralanteriorcingulate.Right | 0.082 | 0.620 | 0.113 | 0.721 | -0.468 | 0.023 *** |
| rostralanteriorcingulate.Left | -0.119 | 0.469 | -0.132 | 0.669 | 0.000 | 0.999 |
| caudalanteriorcingulate.Right | -0.089 | 0.595 | 0.085 | 0.767 | -0.119 | 0.59 |
| caudalanteriorcingulate.Left | 0.106 | 0.519 | 0.046 | 0.872 | -0.036 | 0.869 |
| posteriorcingulate.Right | 0.100 | 0.559 | 0.281 | 0.363 | -0.177 | 0.414 |
| posteriorcingulate.Left | 0.030 | 0.857 | 0.246 | 0.372 | -0.154 | 0.513 |
| isthmuscingulate.Right | -0.061 | 0.739 | 0.366 | 0.224 | -0.181 | 0.424 |
| isthmuscingulate.Left | 0.049 | 0.771 | 0.004 | 0.988 | 0.014 | 0.954 |
| insula.Right | 0.169 | 0.338 | 0.380 | 0.202 | -0.344 | 0.142 |
| insula.Left | 0.096 | 0.587 | 0.313 | 0.307 | -0.195 | 0.43 |
| entorhinal.Right | 0.197 | 0.267 | 0.300 | 0.323 | -0.530 | 0.012 *** |
| entorhinal.Left | 0.138 | 0.436 | 0.450 | 0.140 | -0.404 | 0.055 |
| parahippocampal.Right | 0.102 | 0.567 | -0.048 | 0.886 | -0.496 | 0.022 *** |
| parahippocampal.Left | 0.327 | 0.069 | -0.484 | 0.202 | -0.343 | 0.123 |
| temporalpole.Right | 0.228 | 0.205 | 0.360 | 0.214 | -0.709 | 0 *** |
| temporalpole.Left | -0.099 | 0.580 | 0.172 | 0.556 | -0.463 | 0.028 *** |
| fusiform.Right | 0.198 | 0.269 | 0.554 | 0.076 | -0.543 | 0.015 *** |
| fusiform.Left | -0.025 | 0.887 | 0.114 | 0.721 | -0.331 | 0.223 |
| Right.Accumbens.area | 0.039 | 0.822 | 0.178 | 0.568 | -0.332 | 0.153 |
| Left.Accumbens.area | -0.167 | 0.342 | 0.241 | 0.393 | -0.138 | 0.536 |
| Right.Amygdala | 0.285 | 0.102 | 0.231 | 0.402 | -0.677 | 0.001 *** |
| Left.Amygdala | 0.136 | 0.417 | 0.493 | 0.113 | -0.452 | 0.04 *** |
| Right.Caudate | -0.069 | 0.675 | -0.085 | 0.774 | -0.301 | 0.154 |
| Left.Caudate | -0.121 | 0.464 | -0.086 | 0.784 | -0.376 | 0.072 |
| Right.Hippocampus | 0.075 | 0.697 | -0.026 | 0.939 | -0.328 | 0.18 |
| Left.Hippocampus | -0.200 | 0.267 | 0.059 | 0.858 | -0.150 | 0.534 |

Apathy

|  | AD b | AD p | non.AD b | non.AD p | MCI b | MCI p |
| --- | --- | --- | --- | --- | --- | --- |
| superiorfrontal.Right superiorfrontal.Left | -0.313 -0.173 | 0.066 0.299 | -0.464 -0.591 | 0.074  0.02 *** | -0.203 -0.308 | 0.365 0.185 |
| caudalmiddlefrontal.Right | -0.276 | 0.097 | -0.379 | 0.185 | -0.241 | 0.323 |
| caudalmiddlefrontal.Left | -0.296 | 0.075 | -0.312 | 0.283 | -0.048 | 0.849 |
| rostralmiddlefrontal.Right | -0.119 | 0.476 | -0.431 | 0.135 | -0.117 | 0.633 |
| rostralmiddlefrontal.Left | -0.197 | 0.229 | -0.413 | 0.13 | 0.160 | 0.491 |
| parsopercularis.Right | -0.329 | 0.045 *** | -0.626 | 0.014 *** | -0.303 | 0.168 |
| parsopercularis.Left | -0.258 | 0.114 | -0.384 | 0.19 | -0.012 | 0.961 |
| parsorbitalis.Right | -0.280 | 0.084 | -0.087 | 0.773 | 0.034 | 0.88 |
| parsorbitalis.Left | -0.308 | 0.058 | -0.029 | 0.921 | -0.274 | 0.185 |
| parstriangularis.Right | -0.386 | 0.022 *** | -0.436 | 0.101 | -0.080 | 0.727 |
| parstriangularis.Left | -0.320 | 0.047 *** | -0.270 | 0.357 | 0.072 | 0.75 |
| lateralorbitofrontal.Right | -0.165 | 0.333 | -0.306 | 0.307 | 0.035 | 0.871 |
| lateralorbitofrontal.Left | -0.133 | 0.424 | -0.305 | 0.306 | -0.268 | 0.222 |
| medialorbitofrontal.Right | 0.016 | 0.925 | -0.357 | 0.244 | -0.031 | 0.883 |
| medialorbitofrontal.Left | -0.236 | 0.155 | -0.412 | 0.158 | -0.374 | 0.079 |
| frontalpole.Right | 0.136 | 0.426 | 0.148 | 0.6 | 0.022 | 0.916 |
| frontalpole.Left | 0.053 | 0.754 | -0.184 | 0.554 | -0.073 | 0.73 |
| precentral.Right | -0.281 | 0.118 | -0.330 | 0.279 | -0.396 | 0.091 |
| precentral.Left | -0.387 | 0.024 *** | -0.264 | 0.403 | -0.289 | 0.256 |
| paracentral.Right | -0.078 | 0.666 | -0.260 | 0.444 | -0.357 | 0.092 |
| paracentral.Left | -0.020 | 0.906 | -0.352 | 0.189 | -0.491 | 0.021 *** |
| rostralanteriorcingulate.Right | 0.052 | 0.755 | -0.487 | 0.101 | 0.062 | 0.774 |
| rostralanteriorcingulate.Left | -0.154 | 0.345 | -0.528 | 0.064 | 0.036 | 0.863 |
| caudalanteriorcingulate.Right | 0.248 | 0.131 | 0.265 | 0.341 | 0.312 | 0.148 |
| caudalanteriorcingulate.Left | -0.203 | 0.213 | -0.470 | 0.075 | 0.223 | 0.295 |
| posteriorcingulate.Right | 0.007 | 0.965 | -0.167 | 0.591 | 0.023 | 0.914 |
| posteriorcingulate.Left | -0.089 | 0.593 | -0.513 | 0.044 *** | -0.071 | 0.764 |
| isthmuscingulate.Right | -0.195 | 0.284 | -0.516 | 0.074 | -0.219 | 0.33 |
| isthmuscingulate.Left | -0.100 | 0.551 | -0.553 | 0.037 *** | -0.097 | 0.687 |
| insula.Right | -0.191 | 0.278 | -0.499 | 0.082 | 0.165 | 0.488 |
| insula.Left | 0.001 | 0.997 | -0.290 | 0.342 | 0.319 | 0.189 |
| entorhinal.Right | 0.001 | 0.994 | -0.400 | 0.177 | -0.145 | 0.517 |
| entorhinal.Left | -0.177 | 0.317 | -0.145 | 0.646 | -0.013 | 0.951 |
| parahippocampal.Right | -0.232 | 0.188 | 0.432 | 0.176 | 0.205 | 0.367 |
| parahippocampal.Left | -0.269 | 0.137 | 0.514 | 0.17 | 0.072 | 0.753 |
| temporalpole.Right | -0.075 | 0.677 | -0.094 | 0.752 | 0.102 | 0.645 |
| temporalpole.Left | 0.106 | 0.552 | 0.270 | 0.348 | -0.278 | 0.201 |
| fusiform.Right | 0.143 | 0.425 | -0.485 | 0.125 | -0.122 | 0.606 |
| fusiform.Left | 0.125 | 0.47 | 0.067 | 0.833 | -0.193 | 0.482 |
| Right.Accumbens.area | 0.149 | 0.386 | -0.147 | 0.636 | 0.140 | 0.554 |
| Left.Accumbens.area | -0.192 | 0.271 | -0.012 | 0.965 | 0.364 | 0.094 |
| Right.Amygdala | 0.076 | 0.668 | -0.242 | 0.374 | -0.208 | 0.372 |
| Left.Amygdala | 0.188 | 0.259 | -0.286 | 0.371 | -0.245 | 0.281 |
| Right.Caudate | -0.016 | 0.924 | -0.581 | 0.029 *** | -0.053 | 0.805 |
| Left.Caudate | -0.097 | 0.556 | -0.455 | 0.126 | -0.070 | 0.746 |
| Right.Hippocampus | -0.064 | 0.739 | -0.432 | 0.172 | -0.010 | 0.968 |
| Left.Hippocampus | -0.058 | 0.748 | 0.218 | 0.497 | 0.020 | 0.934 |

Disinhibition

|  | AD b | AD p | non.AD b | non.AD p | MCI b | MCI p |
| --- | --- | --- | --- | --- | --- | --- |
| superiorfrontal.Right superiorfrontal.Left | -0.153 -0.035 | 0.379 0.836 | 0.062 0.011 | 0.828 0.969 | -0.193 -0.139 | 0.389 0.555 |
| caudalmiddlefrontal.Right | -0.073 | 0.668 | 0.233 | 0.437 | -0.415 | 0.082 |
| caudalmiddlefrontal.Left | -0.018 | 0.917 | 0.141 | 0.644 | -0.131 | 0.6 |
| rostralmiddlefrontal.Right | -0.094 | 0.574 | -0.088 | 0.776 | -0.151 | 0.538 |
| rostralmiddlefrontal.Left | -0.120 | 0.468 | -0.089 | 0.761 | -0.022 | 0.926 |
| parsopercularis.Right | -0.170 | 0.31 | 0.111 | 0.708 | 0.084 | 0.708 |
| parsopercularis.Left | -0.169 | 0.306 | 0.213 | 0.490 | 0.094 | 0.704 |
| parsorbitalis.Right | -0.040 | 0.808 | 0.298 | 0.329 | -0.072 | 0.75 |
| parsorbitalis.Left | -0.072 | 0.666 | 0.126 | 0.676 | -0.023 | 0.914 |
| parstriangularis.Right | -0.200 | 0.249 | 0.112 | 0.698 | -0.034 | 0.882 |
| parstriangularis.Left | 0.017 | 0.918 | -0.016 | 0.959 | 0.244 | 0.279 |
| lateralorbitofrontal.Right | 0.195 | 0.253 | -0.170 | 0.586 | -0.126 | 0.552 |
| lateralorbitofrontal.Left | -0.176 | 0.289 | -0.051 | 0.872 | -0.105 | 0.637 |
| medialorbitofrontal.Right | 0.076 | 0.646 | -0.451 | 0.145 | -0.001 | 0.995 |
| medialorbitofrontal.Left | -0.059 | 0.727 | -0.229 | 0.459 | -0.178 | 0.417 |
| frontalpole.Right | 0.094 | 0.583 | -0.228 | 0.428 | -0.052 | 0.804 |
| frontalpole.Left | -0.041 | 0.81 | -0.049 | 0.878 | -0.004 | 0.985 |
| precentral.Right | -0.121 | 0.507 | 0.402 | 0.195 | -0.332 | 0.159 |
| precentral.Left | 0.012 | 0.945 | 0.311 | 0.334 | -0.231 | 0.367 |
| paracentral.Right | -0.024 | 0.896 | 0.368 | 0.285 | -0.323 | 0.129 |
| paracentral.Left | 0.104 | 0.532 | 0.083 | 0.772 | -0.268 | 0.227 |
| rostralanteriorcingulate.Right | 0.007 | 0.966 | -0.322 | 0.307 | -0.250 | 0.244 |
| rostralanteriorcingulate.Left | 0.230 | 0.157 | -0.154 | 0.624 | 0.227 | 0.272 |
| caudalanteriorcingulate.Right | 0.123 | 0.46 | 0.311 | 0.273 | 0.081 | 0.715 |
| caudalanteriorcingulate.Left | 0.171 | 0.298 | 0.024 | 0.934 | 0.052 | 0.809 |
| posteriorcingulate.Right | -0.080 | 0.637 | 0.038 | 0.905 | -0.032 | 0.882 |
| posteriorcingulate.Left | 0.144 | 0.386 | -0.048 | 0.865 | 0.014 | 0.952 |
| isthmuscingulate.Right | 0.049 | 0.79 | -0.261 | 0.402 | -0.241 | 0.283 |
| isthmuscingulate.Left | 0.218 | 0.191 | -0.264 | 0.367 | -0.160 | 0.506 |
| insula.Right | 0.269 | 0.124 | 0.041 | 0.896 | -0.421 | 0.069 |
| insula.Left | 0.495 | 0.003 *** | -0.154 | 0.629 | -0.170 | 0.489 |
| entorhinal.Right | 0.249 | 0.16 | 0.007 | 0.983 | -0.482 | 0.024 *** |
| entorhinal.Left | 0.177 | 0.318 | -0.005 | 0.989 | -0.262 | 0.225 |
| parahippocampal.Right | 0.209 | 0.24 | 0.056 | 0.869 | -0.191 | 0.4 |
| parahippocampal.Left | 0.080 | 0.665 | 0.328 | 0.405 | -0.223 | 0.323 |
| temporalpole.Right | 0.073 | 0.687 | 0.146 | 0.631 | -0.505 | 0.015 *** |
| temporalpole.Left | 0.253 | 0.151 | 0.002 | 0.996 | -0.358 | 0.097 |
| fusiform.Right | 0.330 | 0.061 | -0.190 | 0.575 | -0.536 | 0.016 *** |
| fusiform.Left | 0.282 | 0.1 | 0.269 | 0.400 | -0.302 | 0.266 |
| Right.Accumbens.area | 0.209 | 0.224 | -0.255 | 0.420 | -0.110 | 0.644 |
| Left.Accumbens.area | 0.150 | 0.391 | 0.138 | 0.636 | 0.052 | 0.817 |
| Right.Amygdala | 0.169 | 0.336 | -0.006 | 0.982 | -0.463 | 0.039 *** |
| Left.Amygdala | 0.105 | 0.532 | -0.183 | 0.581 | -0.191 | 0.405 |
| Right.Caudate | 0.281 | 0.083 | 0.003 | 0.993 | 0.010 | 0.964 |
| Left.Caudate | 0.232 | 0.157 | -0.167 | 0.600 | -0.092 | 0.672 |
| Right.Hippocampus | -0.178 | 0.351 | -0.518 | 0.105 | 0.161 | 0.516 |
| Left.Hippocampus | -0.245 | 0.171 | -0.133 | 0.688 | 0.282 | 0.237 |

Irritability

|  | AD b | AD p | non.AD b | non.AD p | MCI b | MCI p |
| --- | --- | --- | --- | --- | --- | --- |
| superiorfrontal.Right superiorfrontal.Left | -0.144 -0.002 | 0.403 0.991 | 0.021 0.081 | 0.939 0.777 | 0.281 0.275 | 0.184 0.214 |
| caudalmiddlefrontal.Right | -0.186 | 0.262 | 0.160 | 0.59 | 0.163 | 0.486 |
| caudalmiddlefrontal.Left | -0.111 | 0.506 | 0.138 | 0.643 | 0.186 | 0.432 |
| rostralmiddlefrontal.Right | -0.139 | 0.398 | -0.237 | 0.429 | 0.505 | 0.022 *** |
| rostralmiddlefrontal.Left | -0.019 | 0.908 | 0.105 | 0.713 | 0.441 | 0.038 *** |
| parsopercularis.Right | -0.268 | 0.101 | 0.180 | 0.533 | 0.462 | 0.022 *** |
| parsopercularis.Left | 0.032 | 0.847 | 0.264 | 0.379 | 0.314 | 0.175 |
| parsorbitalis.Right | -0.267 | 0.096 | 0.062 | 0.839 | 0.393 | 0.057 |
| parsorbitalis.Left | -0.187 | 0.251 | -0.015 | 0.958 | 0.228 | 0.251 |
| parstriangularis.Right | -0.240 | 0.159 | 0.078 | 0.782 | 0.384 | 0.069 |
| parstriangularis.Left | -0.007 | 0.968 | 0.134 | 0.653 | 0.450 | 0.029 *** |
| lateralorbitofrontal.Right | -0.199 | 0.236 | 0.031 | 0.921 | 0.232 | 0.243 |
| lateralorbitofrontal.Left | -0.032 | 0.844 | 0.147 | 0.629 | 0.302 | 0.146 |
| medialorbitofrontal.Right | 0.059 | 0.72 | -0.608 | 0.035 *** | 0.286 | 0.145 |
| medialorbitofrontal.Left | -0.032 | 0.847 | -0.077 | 0.801 | 0.128 | 0.539 |
| frontalpole.Right | 0.130 | 0.441 | -0.395 | 0.148 | 0.330 | 0.088 |
| frontalpole.Left | 0.256 | 0.12 | -0.238 | 0.443 | 0.313 | 0.112 |
| precentral.Right | -0.197 | 0.271 | 0.123 | 0.695 | 0.044 | 0.849 |
| precentral.Left | -0.053 | 0.763 | 0.102 | 0.75 | 0.082 | 0.738 |
| paracentral.Right | -0.137 | 0.442 | 0.065 | 0.85 | 0.049 | 0.814 |
| paracentral.Left | 0.079 | 0.631 | 0.166 | 0.549 | -0.020 | 0.926 |
| rostralanteriorcingulate.Right | 0.180 | 0.266 | -0.372 | 0.224 | 0.205 | 0.317 |
| rostralanteriorcingulate.Left | 0.033 | 0.839 | -0.255 | 0.401 | 0.174 | 0.379 |
| caudalanteriorcingulate.Right | 0.164 | 0.319 | 0.095 | 0.738 | 0.191 | 0.359 |
| caudalanteriorcingulate.Left | 0.071 | 0.665 | 0.262 | 0.346 | -0.055 | 0.79 |
| posteriorcingulate.Right | -0.035 | 0.836 | 0.087 | 0.781 | 0.083 | 0.689 |
| posteriorcingulate.Left | -0.011 | 0.945 | 0.174 | 0.529 | 0.163 | 0.468 |
| isthmuscingulate.Right | -0.152 | 0.399 | -0.062 | 0.842 | -0.142 | 0.51 |
| isthmuscingulate.Left | 0.135 | 0.415 | -0.193 | 0.504 | -0.136 | 0.551 |
| insula.Right | -0.171 | 0.327 | -0.031 | 0.919 | 0.098 | 0.668 |
| insula.Left | -0.119 | 0.493 | -0.012 | 0.968 | 0.240 | 0.304 |
| entorhinal.Right | -0.130 | 0.461 | 0.015 | 0.962 | 0.005 | 0.981 |
| entorhinal.Left | -0.169 | 0.335 | -0.128 | 0.686 | 0.107 | 0.606 |
| parahippocampal.Right | 0.050 | 0.777 | -0.040 | 0.905 | 0.087 | 0.691 |
| parahippocampal.Left | 0.032 | 0.86 | -0.240 | 0.536 | -0.173 | 0.421 |
| temporalpole.Right | -0.349 | 0.045 *** | -0.193 | 0.514 | 0.170 | 0.416 |
| temporalpole.Left | -0.129 | 0.463 | -0.079 | 0.788 | 0.114 | 0.587 |
| fusiform.Right | -0.124 | 0.485 | -0.106 | 0.751 | 0.130 | 0.563 |
| fusiform.Left | -0.024 | 0.891 | -0.051 | 0.872 | 0.129 | 0.622 |
| Right.Accumbens.area | -0.040 | 0.815 | -0.001 | 0.997 | 0.311 | 0.16 |
| Left.Accumbens.area | -0.127 | 0.464 | 0.231 | 0.412 | 0.349 | 0.092 |
| Right.Amygdala | -0.071 | 0.682 | -0.079 | 0.777 | 0.037 | 0.868 |
| Left.Amygdala | -0.038 | 0.817 | -0.151 | 0.642 | 0.117 | 0.595 |
| Right.Caudate | 0.013 | 0.936 | 0.153 | 0.603 | 0.384 | 0.051 |
| Left.Caudate | 0.011 | 0.949 | 0.043 | 0.891 | 0.330 | 0.098 |
| Right.Hippocampus | -0.032 | 0.865 | -0.203 | 0.536 | 0.128 | 0.587 |
| Left.Hippocampus | 0.042 | 0.813 | -0.395 | 0.21 | 0.070 | 0.761 |

Aberrant motor behavior

|  | AD b | AD p | non.AD b | non.AD p | MCI b | MCI p |
| --- | --- | --- | --- | --- | --- | --- |
| superiorfrontal.Right superiorfrontal.Left | -0.063 0.077 | 0.712 0.642 | -0.170 -0.287 | 0.474 0.229 | -0.360 -0.249 | 0.099 0.283 |
| caudalmiddlefrontal.Right | 0.042 | 0.8 | -0.007 | 0.979 | -0.364 | 0.129 |
| caudalmiddlefrontal.Left | 0.060 | 0.721 | -0.171 | 0.502 | -0.220 | 0.373 |
| rostralmiddlefrontal.Right | -0.016 | 0.923 | -0.258 | 0.312 | -0.460 | 0.049 *** |
| rostralmiddlefrontal.Left | -0.045 | 0.783 | -0.356 | 0.129 | -0.380 | 0.092 |
| parsopercularis.Right | 0.016 | 0.922 | -0.221 | 0.367 | -0.242 | 0.273 |
| parsopercularis.Left | -0.079 | 0.629 | -0.140 | 0.592 | -0.212 | 0.386 |
| parsorbitalis.Right | -0.191 | 0.236 | -0.084 | 0.747 | -0.287 | 0.193 |
| parsorbitalis.Left | -0.177 | 0.278 | -0.270 | 0.274 | 0.037 | 0.862 |
| parstriangularis.Right | -0.058 | 0.734 | -0.131 | 0.587 | -0.344 | 0.124 |
| parstriangularis.Left | -0.059 | 0.717 | -0.222 | 0.38 | -0.183 | 0.417 |
| lateralorbitofrontal.Right | 0.050 | 0.767 | -0.380 | 0.13 | -0.340 | 0.096 |
| lateralorbitofrontal.Left | -0.186 | 0.254 | -0.379 | 0.131 | -0.301 | 0.166 |
| medialorbitofrontal.Right | 0.009 | 0.955 | -0.130 | 0.632 | -0.236 | 0.254 |
| medialorbitofrontal.Left | 0.090 | 0.586 | -0.157 | 0.546 | -0.195 | 0.37 |
| frontalpole.Right | 0.134 | 0.426 | -0.195 | 0.421 | -0.143 | 0.49 |
| frontalpole.Left | 0.059 | 0.724 | -0.127 | 0.636 | -0.161 | 0.444 |
| precentral.Right | -0.008 | 0.965 | 0.062 | 0.819 | -0.292 | 0.216 |
| precentral.Left | 0.075 | 0.668 | -0.079 | 0.774 | -0.101 | 0.695 |
| paracentral.Right | -0.121 | 0.496 | 0.081 | 0.785 | -0.172 | 0.426 |
| paracentral.Left | 0.135 | 0.407 | -0.155 | 0.516 | 0.010 | 0.964 |
| rostralanteriorcingulate.Right | 0.088 | 0.589 | 0.031 | 0.91 | -0.516 | 0.011 *** |
| rostralanteriorcingulate.Left | -0.100 | 0.536 | -0.196 | 0.453 | 0.062 | 0.767 |
| caudalanteriorcingulate.Right | 0.048 | 0.771 | -0.055 | 0.823 | -0.055 | 0.803 |
| caudalanteriorcingulate.Left | -0.070 | 0.665 | -0.272 | 0.251 | -0.017 | 0.935 |
| posteriorcingulate.Right | -0.078 | 0.642 | -0.331 | 0.202 | 0.011 | 0.959 |
| posteriorcingulate.Left | -0.017 | 0.919 | -0.399 | 0.075 | 0.176 | 0.453 |
| isthmuscingulate.Right | -0.291 | 0.1 | -0.149 | 0.573 | 0.059 | 0.796 |
| isthmuscingulate.Left | -0.260 | 0.11 | -0.077 | 0.758 | 0.481 | 0.035 *** |
| insula.Right | -0.010 | 0.955 | -0.081 | 0.757 | 0.011 | 0.962 |
| insula.Left | -0.109 | 0.528 | -0.078 | 0.77 | -0.032 | 0.898 |
| entorhinal.Right | 0.020 | 0.908 | 0.219 | 0.403 | -0.015 | 0.948 |
| entorhinal.Left | -0.351 | 0.039 *** | 0.215 | 0.425 | -0.022 | 0.921 |
| parahippocampal.Right | -0.005 | 0.977 | 0.165 | 0.563 | -0.065 | 0.776 |
| parahippocampal.Left | -0.164 | 0.361 | 0.669 | 0.027 *** | -0.054 | 0.811 |
| temporalpole.Right | -0.037 | 0.836 | 0.450 | 0.058 | -0.298 | 0.167 |
| temporalpole.Left | -0.242 | 0.162 | 0.291 | 0.236 | 0.058 | 0.791 |
| fusiform.Right | 0.028 | 0.876 | -0.132 | 0.643 | -0.358 | 0.119 |
| fusiform.Left | 0.020 | 0.909 | 0.249 | 0.354 | -0.037 | 0.894 |
| Right.Accumbens.area | 0.102 | 0.549 | -0.235 | 0.376 | -0.213 | 0.362 |
| Left.Accumbens.area | -0.104 | 0.547 | 0.028 | 0.908 | -0.378 | 0.08 |
| Right.Amygdala | 0.104 | 0.549 | 0.225 | 0.339 | -0.233 | 0.314 |
| Left.Amygdala | 0.049 | 0.766 | 0.096 | 0.733 | -0.052 | 0.82 |
| Right.Caudate | -0.014 | 0.931 | -0.037 | 0.884 | -0.223 | 0.291 |
| Left.Caudate | 0.072 | 0.658 | 0.041 | 0.88 | -0.267 | 0.207 |
| Right.Hippocampus | 0.135 | 0.473 | -0.514 | 0.05 | 0.108 | 0.661 |
| Left.Hippocampus | 0.004 | 0.984 | 0.032 | 0.91 | 0.300 | 0.205 |

ù

Night-time behaviour disturbances

|  | AD b | AD p | non.AD b | non.AD p | MCI b | MCI p |
| --- | --- | --- | --- | --- | --- | --- |
| superiorfrontal.Right superiorfrontal.Left | -0.205 -0.206 | 0.216 0.197 | -0.091 -0.054 | 0.722 0.837 | 0.028 0.138 | 0.9 0.555 |
| caudalmiddlefrontal.Right | -0.251 | 0.113 | -0.021 | 0.94 | -0.071 | 0.773 |
| caudalmiddlefrontal.Left | -0.209 | 0.192 | -0.138 | 0.615 | 0.137 | 0.581 |
| rostralmiddlefrontal.Right | -0.098 | 0.535 | -0.425 | 0.109 | 0.041 | 0.868 |
| rostralmiddlefrontal.Left | -0.132 | 0.399 | -0.127 | 0.629 | -0.011 | 0.964 |
| parsopercularis.Right | -0.183 | 0.26 | 0.051 | 0.85 | 0.401 | 0.062 |
| parsopercularis.Left | -0.178 | 0.258 | -0.016 | 0.955 | 0.417 | 0.08 |
| parsorbitalis.Right | -0.206 | 0.184 | -0.251 | 0.363 | 0.220 | 0.323 |
| parsorbitalis.Left | -0.192 | 0.221 | -0.192 | 0.476 | 0.072 | 0.731 |
| parstriangularis.Right | -0.255 | 0.126 | -0.074 | 0.777 | 0.204 | 0.369 |
| parstriangularis.Left | -0.128 | 0.412 | -0.415 | 0.112 | 0.280 | 0.209 |
| lateralorbitofrontal.Right | -0.071 | 0.666 | -0.487 | 0.064 | -0.042 | 0.842 |
| lateralorbitofrontal.Left | -0.060 | 0.706 | -0.290 | 0.294 | 0.227 | 0.301 |
| medialorbitofrontal.Right | -0.114 | 0.469 | -0.310 | 0.277 | -0.092 | 0.66 |
| medialorbitofrontal.Left | -0.052 | 0.747 | -0.189 | 0.499 | 0.318 | 0.137 |
| frontalpole.Right | 0.018 | 0.911 | -0.572 | 0.014 *** | -0.079 | 0.706 |
| frontalpole.Left | -0.090 | 0.59 | -0.254 | 0.373 | 0.239 | 0.251 |
| precentral.Right | -0.187 | 0.28 | -0.103 | 0.721 | 0.040 | 0.868 |
| precentral.Left | -0.329 | 0.046 *** | -0.268 | 0.357 | 0.103 | 0.687 |
| paracentral.Right | -0.369 | 0.029 *** | -0.170 | 0.59 | 0.063 | 0.773 |
| paracentral.Left | -0.109 | 0.489 | 0.150 | 0.556 | 0.117 | 0.601 |
| rostralanteriorcingulate.Right | -0.020 | 0.899 | -0.100 | 0.73 | 0.063 | 0.772 |
| rostralanteriorcingulate.Left | 0.058 | 0.711 | -0.293 | 0.291 | -0.030 | 0.884 |
| caudalanteriorcingulate.Right | -0.078 | 0.637 | -0.244 | 0.343 | 0.306 | 0.155 |
| caudalanteriorcingulate.Left | 0.058 | 0.711 | 0.055 | 0.832 | -0.024 | 0.911 |
| posteriorcingulate.Right | -0.021 | 0.898 | 0.141 | 0.622 | 0.125 | 0.563 |
| posteriorcingulate.Left | -0.010 | 0.948 | 0.065 | 0.799 | 0.172 | 0.464 |
| isthmuscingulate.Right | -0.108 | 0.534 | -0.014 | 0.961 | 0.005 | 0.984 |
| isthmuscingulate.Left | -0.028 | 0.859 | 0.107 | 0.69 | 0.051 | 0.833 |
| insula.Right | -0.013 | 0.939 | -0.222 | 0.426 | -0.080 | 0.738 |
| insula.Left | -0.159 | 0.341 | -0.311 | 0.267 | -0.104 | 0.672 |
| entorhinal.Right | -0.033 | 0.849 | 0.034 | 0.906 | -0.146 | 0.513 |
| entorhinal.Left | 0.131 | 0.461 | -0.089 | 0.761 | 0.061 | 0.781 |
| parahippocampal.Right | -0.018 | 0.916 | -0.176 | 0.563 | 0.023 | 0.918 |
| parahippocampal.Left | -0.097 | 0.578 | -0.420 | 0.23 | 0.173 | 0.442 |
| temporalpole.Right | 0.014 | 0.937 | -0.044 | 0.873 | -0.277 | 0.199 |
| temporalpole.Left | -0.008 | 0.964 | -0.196 | 0.464 | -0.237 | 0.277 |
| fusiform.Right | 0.014 | 0.934 | -0.061 | 0.841 | -0.160 | 0.496 |
| fusiform.Left | 0.031 | 0.853 | -0.157 | 0.591 | 0.040 | 0.886 |
| Right.Accumbens.area | 0.272 | 0.099 | -0.224 | 0.432 | -0.311 | 0.179 |
| Left.Accumbens.area | 0.143 | 0.395 | 0.033 | 0.901 | -0.077 | 0.731 |
| Right.Amygdala | 0.211 | 0.212 | -0.095 | 0.711 | -0.485 | 0.029 *** |
| Left.Amygdala | -0.059 | 0.711 | -0.079 | 0.793 | -0.269 | 0.235 |
| Right.Caudate | 0.200 | 0.2 | 0.256 | 0.338 | 0.029 | 0.892 |
| Left.Caudate | 0.096 | 0.547 | 0.321 | 0.254 | 0.067 | 0.757 |
| Right.Hippocampus | 0.183 | 0.318 | -0.281 | 0.348 | -0.140 | 0.571 |
| Left.Hippocampus | 0.188 | 0.277 | -0.538 | 0.052 | 0.055 | 0.819 |

Eating abnormalities

|  | AD b | AD p | non.AD b | non.AD p | MCI b | MCI p |
| --- | --- | --- | --- | --- | --- | --- |
| superiorfrontal.Right superiorfrontal.Left | -0.214 -0.045 | 0.217 0.79 | -0.113 -0.255 | 0.666 0.332 | -0.259 -0.176 | 0.24 0.449 |
| caudalmiddlefrontal.Right | -0.172 | 0.309 | -0.160 | 0.566 | -0.460 | 0.05 |
| caudalmiddlefrontal.Left | -0.102 | 0.55 | -0.104 | 0.711 | -0.090 | 0.717 |
| rostralmiddlefrontal.Right | -0.124 | 0.458 | -0.166 | 0.559 | -0.309 | 0.195 |
| rostralmiddlefrontal.Left | -0.126 | 0.445 | -0.104 | 0.699 | -0.230 | 0.316 |
| parsopercularis.Right | -0.337 | 0.04 *** | -0.279 | 0.295 | -0.155 | 0.484 |
| parsopercularis.Left | -0.296 | 0.07 | -0.141 | 0.62 | -0.158 | 0.517 |
| parsorbitalis.Right | -0.213 | 0.195 | 0.107 | 0.707 | -0.311 | 0.154 |
| parsorbitalis.Left | -0.246 | 0.135 | 0.047 | 0.866 | -0.101 | 0.626 |
| parstriangularis.Right | -0.334 | 0.051 | -0.242 | 0.354 | 0.026 | 0.91 |
| parstriangularis.Left | -0.203 | 0.216 | -0.072 | 0.798 | -0.108 | 0.631 |
| lateralorbitofrontal.Right | -0.048 | 0.781 | -0.286 | 0.311 | -0.202 | 0.329 |
| lateralorbitofrontal.Left | -0.225 | 0.174 | -0.356 | 0.201 | -0.209 | 0.339 |
| medialorbitofrontal.Right | -0.162 | 0.327 | -0.018 | 0.953 | -0.211 | 0.305 |
| medialorbitofrontal.Left | -0.165 | 0.327 | -0.500 | 0.061 | -0.181 | 0.403 |
| frontalpole.Right | 0.052 | 0.764 | 0.280 | 0.285 | -0.174 | 0.4 |
| frontalpole.Left | 0.034 | 0.84 | -0.005 | 0.988 | -0.177 | 0.396 |
| precentral.Right | -0.007 | 0.972 | -0.160 | 0.585 | -0.184 | 0.437 |
| precentral.Left | -0.130 | 0.466 | -0.248 | 0.405 | -0.185 | 0.466 |
| paracentral.Right | -0.028 | 0.878 | -0.279 | 0.383 | -0.262 | 0.217 |
| paracentral.Left | 0.047 | 0.778 | -0.039 | 0.882 | -0.192 | 0.386 |
| rostralanteriorcingulate.Right | 0.017 | 0.919 | -0.171 | 0.562 | -0.298 | 0.158 |
| rostralanteriorcingulate.Left | -0.128 | 0.437 | -0.222 | 0.438 | -0.011 | 0.958 |
| caudalanteriorcingulate.Right | 0.022 | 0.897 | -0.038 | 0.886 | -0.032 | 0.885 |
| caudalanteriorcingulate.Left | 0.022 | 0.895 | -0.235 | 0.369 | 0.109 | 0.608 |
| posteriorcingulate.Right | -0.006 | 0.974 | -0.179 | 0.539 | -0.053 | 0.804 |
| posteriorcingulate.Left | -0.060 | 0.72 | -0.345 | 0.171 | -0.062 | 0.79 |
| isthmuscingulate.Right | -0.176 | 0.336 | -0.305 | 0.282 | -0.016 | 0.942 |
| isthmuscingulate.Left | -0.045 | 0.788 | 0.012 | 0.965 | 0.048 | 0.841 |
| insula.Right | 0.025 | 0.89 | -0.284 | 0.316 | -0.245 | 0.295 |
| insula.Left | 0.020 | 0.912 | -0.571 | 0.032 *** | -0.175 | 0.471 |
| entorhinal.Right | -0.111 | 0.538 | -0.392 | 0.16 | -0.501 | 0.017 *** |
| entorhinal.Left | -0.148 | 0.406 | -0.404 | 0.159 | -0.384 | 0.066 |
| parahippocampal.Right | -0.046 | 0.797 | 0.556 | 0.055 | -0.493 | 0.021 *** |
| parahippocampal.Left | 0.117 | 0.523 | -0.261 | 0.473 | -0.311 | 0.157 |
| temporalpole.Right | -0.015 | 0.936 | -0.430 | 0.104 | -0.680 | 0 *** |
| temporalpole.Left | 0.018 | 0.918 | -0.211 | 0.44 | -0.466 | 0.025 *** |
| fusiform.Right | 0.181 | 0.313 | -0.326 | 0.286 | -0.470 | 0.035 *** |
| fusiform.Left | -0.015 | 0.931 | -0.412 | 0.15 | -0.354 | 0.186 |
| Right.Accumbens.area | 0.125 | 0.469 | 0.090 | 0.759 | -0.116 | 0.62 |
| Left.Accumbens.area | -0.022 | 0.899 | -0.168 | 0.528 | 0.009 | 0.968 |
| Right.Amygdala | 0.166 | 0.347 | -0.103 | 0.694 | -0.603 | 0.005 *** |
| Left.Amygdala | 0.248 | 0.135 | -0.454 | 0.119 | -0.388 | 0.079 |
| Right.Caudate | 0.108 | 0.513 | -0.199 | 0.468 | -0.314 | 0.13 |
| Left.Caudate | 0.032 | 0.849 | -0.218 | 0.454 | -0.363 | 0.079 |
| Right.Hippocampus | 0.006 | 0.976 | 0.253 | 0.409 | -0.300 | 0.214 |
| Left.Hippocampus | 0.006 | 0.973 | 0.027 | 0.93 | -0.228 | 0.335 |

**Table S6.** Associations between NPI sub-domains scores (numerical variables) and CT of cortical ROIs and V of subcortical ROIs. Coefficients and p-values of the multivariate linear regression adjusted for age, disease duration and MMSE score are shown.

Delusions

| AD_b | AD_p | non.AD_b | non.AD_p | MCI_b | MCI_p |
| --- | --- | --- | --- | --- | --- |
| superiorfrontal.Right -0.238 | 0.18 | 0.114 | 0.713 | 0.028 | 0.906 |
| superiorfrontal.Left -0.062 | 0.723 | 0.054 | 0.882 | 0.014 | 0.956 |
| caudalmiddlefrontal.Right -0.110 | 0.524 | 0.365 | 0.227 | -0.122 | 0.635 |
| caudalmiddlefrontal.Left -0.078 | 0.68 | 0.079 | 0.85 | -0.065 | 0.798 |
| rostralmiddlefrontal.Right -0.131 | 0.436 | -0.160 | 0.596 | 0.208 | 0.412 |
| rostralmiddlefrontal.Left -0.121 | 0.475 | -0.101 | 0.73 | 0.209 | 0.392 |
| parsopercularis.Right -0.259 | 0.129 | 0.099 | 0.772 | 0.340 | 0.129 |
| parsopercularis.Left -0.131 | 0.458 | 0.233 | 0.493 | 0.109 | 0.668 |
| parsorbitalis.Right -0.332 | 0.039 *** | 0.192 | 0.523 | 0.183 | 0.419 |
| parsorbitalis.Left -0.272 | 0.1 | 0.119 | 0.719 | 0.233 | 0.271 |
| parstriangularis.Right -0.274  parstriangularis.Left -0.120  lateralorbitofrontal.Right -0.126 | 0.118  0.479  0.477 | -0.039  0.011  -0.359 | 0.894  0.971  0.27 | 0.086  0.462  0.158 | 0.712  0.036 *** 0.464 |
| lateralorbitofrontal.Left -0.269 | 0.108 | -0.050 | 0.89 | 0.106 | 0.639 |
| medialorbitofrontal.Right -0.137 | 0.408 | -0.303 | 0.307 | 0.217 | 0.306 |
| medialorbitofrontal.Left -0.119 | 0.485 | -0.102 | 0.733 | -0.026 | 0.91 |
| frontalpole.Right 0.035 | 0.839 | -0.413 | 0.124 | 0.014 | 0.949 |
| frontalpole.Left 0.011 | 0.948 | -0.221 | 0.457 | 0.102 | 0.639 |
| precentral.Right -0.058 | 0.753 | 0.396 | 0.199 | -0.135 | 0.589 |
| precentral.Left 0.013 | 0.941 | 0.336 | 0.334 | -0.060 | 0.819 |
| paracentral.Right -0.148 | 0.422 | 0.036 | 0.918 | -0.085 | 0.704 |
| paracentral.Left 0.081 | 0.632 | 0.118 | 0.724 | -0.120 | 0.603 |
| rostralanteriorcingulate.Right 0.078 | 0.646 | -0.247 | 0.446 | 0.083 | 0.716 |
| rostralanteriorcingulate.Left -0.150 | 0.366 | -0.216 | 0.495 | 0.514 | 0.033 *** |
| caudalanteriorcingulate.Right -0.049 | 0.77 | 0.174 | 0.525 | 0.275 | 0.214 |
| caudalanteriorcingulate.Left 0.136 | 0.415 | -0.165 | 0.616 | 0.144 | 0.508 |
| posteriorcingulate.Right -0.037 | 0.834 | 0.010 | 0.977 | 0.165 | 0.451 |
| posteriorcingulate.Left -0.043 | 0.8 | -0.107 | 0.821 | 0.183 | 0.443 |
| isthmuscingulate.Right -0.216 | 0.236 | 0.529 | 0.348 | -0.005 | 0.982 |
| isthmuscingulate.Left -0.015 | 0.931 | 0.166 | 0.697 | -0.115 | 0.637 |
| insula.Right -0.069 | 0.7 | 0.321 | 0.365 | -0.128 | 0.597 |
| insula.Left -0.178 | 0.318 | -0.023 | 0.948 | 0.019 | 0.941 |
| entorhinal.Right -0.168 | 0.347 | 0.564 | 0.105 | -0.099 | 0.664 |
| entorhinal.Left -0.380  parahippocampal.Right -0.111  parahippocampal.Left -0.112  temporalpole.Right -0.222 | 0.029 *** 0.538 0.548 0.219 | 0.545  0.223  -0.179  0.602 | 0.155  0.503  0.65  0.049 *** | 0.061  0.230  -0.053  0.041 | 0.785  0.316  0.817  0.858 |
| temporalpole.Left -0.151 | 0.396 | 0.252 | 0.445 | 0.106 | 0.638 |
| fusiform.Right -0.147 | 0.418 | 0.073 | 0.89 | -0.018 | 0.941 |
| fusiform.Left -0.089 | 0.612 | 0.126 | 0.73 | 0.086 | 0.758 |
| Right.Accumbens.area 0.054 | 0.768 | 0.013 | 0.967 | 0.188 | 0.432 |
| Left.Accumbens.area -0.062 | 0.733 | 0.353 | 0.216 | 0.278 | 0.224 |
| Right.Amygdala -0.108 | 0.55 | 0.456 | 0.075 | 0.124 | 0.602 |
| Left.Amygdala -0.154 | 0.364 | 0.679 | 0.13 | 0.288 | 0.219 |
| Right.Caudate 0.024 | 0.897 | 0.091 | 0.802 | 0.362 | 0.11 |
| Left.Caudate -0.035 | 0.848 | -0.048 | 0.888 | 0.285 | 0.234 |
| Right.Hippocampus -0.115 | 0.555 | -0.170 | 0.623 | 0.624 | 0.012 *** |
| Left.Hippocampus -0.140 | 0.44 | 0.028 | 0.941 | 0.482 | 0.041 *** |

Hallucinations

| AD_b | AD_p | non.AD_b | non.AD_p | MCI_b | MCI_p |
| --- | --- | --- | --- | --- | --- |
| iorfrontal.Right -0.142 0.418 0.169 0.572 non calcolabile non calcolabile | | | | | |

super

superiorfrontal.Left 0.021 0.901 -0.035 0.914 non calcolabile non calcolabile

caudalmiddlefrontal.Right 0.014 0.933 0.315 0.27 non calcolabile non calcolabile

caudalmiddlefrontal.Left 0.104 0.573 0.152 0.668 non calcolabile non calcolabile

rostralmiddlefrontal.Right -0.067 0.686 0.048 0.872 non calcolabile non calcolabile

rostralmiddlefrontal.Left -0.126 0.448 -0.150 0.602 non calcolabile non calcolabile

parsopercularis.Right -0.138 0.416 0.061 0.853 non calcolabile non calcolabile

parsopercularis.Left 0.016 0.925 0.188 0.555 non calcolabile non calcolabile

parsorbitalis.Right -0.242 0.131 0.235 0.42 non calcolabile non calcolabile

parsorbitalis.Left -0.168 0.308 0.005 0.987 non calcolabile non calcolabile

parstriangularis.Right -0.218 0.206 0.092 0.749 non calcolabile non calcolabile

parstriangularis.Left -0.178 0.282 0.146 0.621 non calcolabile non calcolabile

lateralorbitofrontal.Right 0.010 0.953 -0.086 0.788 non calcolabile non calcolabile

lateralorbitofrontal.Left -0.167 0.313 -0.032 0.923 non calcolabile non calcolabile

medialorbitofrontal.Right -0.128 0.429 -0.044 0.883 non calcolabile non calcolabile

medialorbitofrontal.Left -0.054 0.749 -0.052 0.859 non calcolabile non calcolabile

frontalpole.Right -0.105 0.536 -0.006 0.983 non calcolabile non calcolabile

frontalpole.Left -0.130 0.437 -0.066 0.825 non calcolabile non calcolabile

precentral.Right 0.057 0.754 0.469 0.114 non calcolabile non calcolabile

precentral.Left 0.099 0.58 0.391 0.219 non calcolabile non calcolabile

paracentral.Right -0.098 0.589 0.316 0.348 non calcolabile non calcolabile

paracentral.Left 0.034 0.838 -0.017 0.959 non calcolabile non calcolabile

rostralanteriorcingulate.Right 0.040 0.809 0.142 0.663 non calcolabile non calcolabile

rostralanteriorcingulate.Left -0.066 0.689 -0.030 0.918 non calcolabile non calcolabile

caudalanteriorcingulate.Right -0.139 0.399 0.165 0.543 non calcolabile non calcolabile

caudalanteriorcingulate.Left 0.076 0.645 -0.175 0.569 non calcolabile non calcolabile

posteriorcingulate.Right 0.031 0.86 -0.263 0.37 non calcolabile non calcolabile

posteriorcingulate.Left 0.070 0.672 -0.347 0.243 non calcolabile non calcolabile

isthmuscingulate.Right -0.063 0.728 0.054 0.861 non calcolabile non calcolabile

isthmuscingulate.Left -0.086 0.603 0.044 0.889 non calcolabile non calcolabile

insula.Right 0.081 0.644 0.439 0.176 non calcolabile non calcolabile

insula.Left -0.095 0.588 0.113 0.711 non calcolabile non calcolabile

entorhinal.Right -0.015 0.934 0.465 0.113 non calcolabile non calcolabile

entorhinal.Left -0.348 0.043 *** 0.386 0.194 non calcolabile non calcolabile

parahippocampal.Right -0.030 0.866 0.304 0.332 non calcolabile non calcolabile

parahippocampal.Left -0.294 0.103 0.506 0.18 non calcolabile non calcolabile

temporalpole.Right 0.006 0.975 0.549 0.032 *** non calcolabile non calcolabile

temporalpole.Left -0.088 0.616 0.306 0.269 non calcolabile non calcolabile

fusiform.Right -0.085 0.633 0.156 0.698 non calcolabile non calcolabile

fusiform.Left -0.033 0.847 0.242 0.419 non calcolabile non calcolabile

Right.Accumbens.area 0.216 0.22 0.053 0.863 non calcolabile non calcolabile

Left.Accumbens.area 0.029 0.868 0.190 0.479 non calcolabile non calcolabile

Right.Amygdala -0.164 0.353 0.521 0.032 *** non calcolabile non calcolabile

Left.Amygdala -0.191 0.249 0.245 0.428 non calcolabile non calcolabile

Right.Caudate 0.034 0.853 0.040 0.901 non calcolabile non calcolabile

Left.Caudate 0.060 0.737 -0.078 0.798 non calcolabile non calcolabile

Right.Hippocampus -0.019 0.923 -0.256 0.424 non calcolabile non calcolabile

Left.Hippocampus -0.201 0.255 0.206 0.502 non calcolabile non calcolabile

Table 2: Allucinazioni

Agitation

| AD_b | AD_p | non.AD_b | non.AD_p | MCI_b | MCI_p |
| --- | --- | --- | --- | --- | --- |
| superiorfrontal.Right -0.313 | 0.075 | -0.060 | 0.858 | 0.045 | 0.850 |
| superiorfrontal.Left -0.030 | 0.865 | -0.078 | 0.825 | 0.080 | 0.746 |
| caudalmiddlefrontal.Right -0.344 | 0.04 *** | 0.079 | 0.81 | -0.231 | 0.354 |
| caudalmiddlefrontal.Left -0.101 | 0.591 | -0.121 | 0.759 | 0.020 | 0.936 |
| rostralmiddlefrontal.Right -0.259 | 0.118 | -0.427 | 0.175 | 0.231 | 0.351 |
| rostralmiddlefrontal.Left -0.094 | 0.582 | -0.096 | 0.767 | 0.306 | 0.197 |
| parsopercularis.Right -0.440 | 0.008 *** | -0.030 | 0.934 | 0.143 | 0.523 |
| parsopercularis.Left -0.054 | 0.761 | 0.045 | 0.899 | 0.150 | 0.547 |
| parsorbitalis.Right -0.299 | 0.066 | -0.102 | 0.757 | 0.094 | 0.675 |
| parsorbitalis.Left -0.306 | 0.064 | -0.085 | 0.796 | 0.094 | 0.654 |
| parstriangularis.Right -0.439 | 0.01 *** | -0.174 | 0.586 | 0.234 | 0.301 |
| parstriangularis.Left -0.197 | 0.244 | -0.302 | 0.353 | 0.241 | 0.282 |
| lateralorbitofrontal.Right -0.245  lateralorbitofrontal.Left -0.080 | 0.163  0.64 | -0.660  -0.557 | 0.04 *** 0.105 | 0.032  0.066 | 0.881  0.766 |
| medialorbitofrontal.Right -0.102 | 0.541 | -0.396 | 0.223 | 0.133 | 0.524 |
| medialorbitofrontal.Left -0.139 | 0.414 | -0.395 | 0.209 | -0.075 | 0.745 |
| frontalpole.Right 0.051 | 0.77 | -0.548 | 0.052 | 0.170 | 0.412 |
| frontalpole.Left 0.071 | 0.681 | -0.251 | 0.444 | 0.157 | 0.462 |
| precentral.Right -0.234 | 0.202 | -0.044 | 0.899 | -0.224 | 0.356 |
| precentral.Left -0.207 | 0.251 | -0.317 | 0.38 | -0.182 | 0.477 |
| paracentral.Right -0.180 | 0.327 | -0.257 | 0.498 | -0.329 | 0.123 |
| paracentral.Left -0.085 | 0.614 | 0.104 | 0.775 | -0.315 | 0.157 |
| rostralanteriorcingulate.Right 0.131 | 0.438 | -0.220 | 0.543 | -0.112 | 0.613 |
| rostralanteriorcingulate.Left -0.215 | 0.194 | -0.294 | 0.361 | 0.235 | 0.342 |
| caudalanteriorcingulate.Right 0.118 | 0.483 | -0.135 | 0.655 | -0.004 | 0.987 |
| caudalanteriorcingulate.Left 0.027 | 0.873 | -0.059 | 0.864 | -0.047 | 0.828 |
| posteriorcingulate.Right -0.129 | 0.468 | 0.012 | 0.97 | -0.108 | 0.616 |
| posteriorcingulate.Left -0.117 | 0.486 | -0.118 | 0.729 | -0.063 | 0.789 |
| isthmuscingulate.Right -0.035 | 0.849 | -0.141 | 0.685 | -0.356 | 0.108 |
| isthmuscingulate.Left 0.217 | 0.195 | 0.187 | 0.589 | -0.315 | 0.181 |
| insula.Right -0.272 | 0.123 | -0.365 | 0.322 | -0.213 | 0.366 |
| insula.Left -0.274 | 0.121 | -0.483 | 0.136 | -0.102 | 0.688 |
| entorhinal.Right -0.244 | 0.171 | -0.037 | 0.914 | -0.361 | 0.096 |
| entorhinal.Left -0.191  parahippocampal.Right -0.175 | 0.286  0.33 | -0.224  0.076 | 0.511  0.831 | -0.233  -0.239 | 0.278  0.288 |
| parahippocampal.Left -0.175 | 0.348 | -0.460 | 0.281 | -0.401 | 0.065 |
| temporalpole.Right -0.386  temporalpole.Left -0.235 | 0.029 *** 0.184 | -0.098  -0.262 | 0.757  0.402 | -0.297  -0.231 | 0.173  0.288 |
| fusiform.Right -0.150 | 0.41 | -0.398 | 0.368 | -0.428 | 0.059 |
| fusiform.Left -0.098 | 0.578 | -0.258 | 0.441 | -0.210 | 0.441 |
| Right.Accumbens.area 0.069 | 0.702 | -0.136 | 0.687 | 0.200 | 0.394 |
| Left.Accumbens.area -0.022 | 0.903 | 0.001 | 0.998 | 0.312 | 0.162 |
| Right.Amygdala -0.181 | 0.313 | 0.003 | 0.991 | -0.363 | 0.112 |
| Left.Amygdala -0.060 | 0.727 | -0.202 | 0.561 | -0.155 | 0.504 |
| Right.Caudate 0.235 | 0.196 | 0.232 | 0.511 | 0.100 | 0.663 |
| Left.Caudate 0.175 | 0.335 | 0.181 | 0.591 | -0.004 | 0.986 |
| Right.Hippocampus -0.177 | 0.362 | -0.141 | 0.696 | 0.056 | 0.830 |
| Left.Hippocampus -0.073 | 0.69 | -0.441 | 0.184 | -0.017 | 0.944 |

Depression

| superiorfrontal.Right | AD_b  0.142 | AD_p no  0.440 | on.AD_b non.AD_p  0.172 0.562 | | MCI_b  -0.236 | MCI_p  0.239 |
| --- | --- | --- | --- | --- | --- | --- |
| superiorfrontal.Left | 0.202 | 0.257 | -0.141 | 0.654 | -0.196 | 0.355 |
| caudalmiddlefrontal.Right | 0.065 | 0.711 | 0.272 | 0.340 | -0.367 | 0.08 |
| caudalmiddlefrontal.Left | 0.120 | 0.534 | -0.216 | 0.535 | -0.111 | 0.605 |
| rostralmiddlefrontal.Right | 0.062 | 0.718 | 0.051 | 0.860 | -0.058 | 0.789 |
| rostralmiddlefrontal.Left | 0.059 | 0.736 | -0.152 | 0.593 | -0.113 | 0.588 |
| parsopercularis.Right | 0.121 | 0.497 | 0.110 | 0.736 | 0.079 | 0.687 |
| parsopercularis.Left | 0.053 | 0.769 | 0.065 | 0.837 | -0.229 | 0.281 |
| parsorbitalis.Right | -0.228 | 0.175 | 0.213 | 0.462 | -0.108 | 0.577 |
| parsorbitalis.Left | -0.131 | 0.447 | 0.007 | 0.982 | 0.129 | 0.475 |
| parstriangularis.Right | -0.117 | 0.521 | 0.035 | 0.904 | 0.119 | 0.546 |
| parstriangularis.Left | 0.076 | 0.662 | 0.116 | 0.694 | 0.156 | 0.422 |
| lateralorbitofrontal.Right | -0.019 | 0.917 | -0.183 | 0.560 | 0.150 | 0.411 |
| lateralorbitofrontal.Left | 0.077 | 0.657 | 0.116 | 0.720 | -0.028 | 0.885 |
| medialorbitofrontal.Right | -0.084 | 0.620 | 0.055 | 0.855 | -0.023 | 0.898 |
| medialorbitofrontal.Left | 0.020 | 0.907 | -0.057 | 0.844 | -0.175 | 0.374 |
| frontalpole.Right | 0.222 | 0.208 | -0.029 | 0.916 | -0.089 | 0.62 |
| frontalpole.Left | 0.158 | 0.366 | -0.149 | 0.613 | -0.069 | 0.709 |
| precentral.Right | 0.225 | 0.230 | 0.444 | 0.133 | -0.180 | 0.392 |
| precentral.Left | 0.291 | 0.113 | 0.425 | 0.174 | 0.017 | 0.941 |
| paracentral.Right | 0.199 | 0.290 | 0.113 | 0.739 | -0.247 | 0.184 |
| paracentral.Left | 0.242 | 0.155 | 0.069 | 0.832 | -0.386 | 0.039 *** |
| rostralanteriorcingulate.Right | 0.246 | 0.149 | -0.085 | 0.792 | 0.123 | 0.522 |
| rostralanteriorcingulate.Left | -0.005 | 0.975 | -0.205 | 0.478 | 0.274 | 0.195 |
| caudalanteriorcingulate.Right | 0.030 | 0.862 | 0.244 | 0.357 | -0.048 | 0.801 |
| caudalanteriorcingulate.Left | 0.194 | 0.254 | -0.374 | 0.205 | 0.324 | 0.07 |
| posteriorcingulate.Right | 0.335 | 0.059 | -0.107 | 0.716 | 0.261 | 0.154 |
| posteriorcingulate.Left | 0.151 | 0.377 | -0.342 | 0.245 | 0.384 | 0.049 *** |
| isthmuscingulate.Right | -0.233 | 0.212 | -0.008 | 0.980 | 0.002 | 0.991 |
| isthmuscingulate.Left | -0.110 | 0.525 | -0.175 | 0.571 | 0.008 | 0.968 |
| insula.Right | -0.056 | 0.759 | 0.460 | 0.151 | -0.154 | 0.45 |
| insula.Left | -0.283 | 0.117 | -0.131 | 0.664 | -0.132 | 0.545 |
| entorhinal.Right | -0.238 | 0.192 | 0.231 | 0.448 | -0.308 | 0.1 |
| entorhinal.Left | -0.318 | 0.078 | 0.222 | 0.464 | -0.288 | 0.115 |
| parahippocampal.Right | 0.062 | 0.739 | 0.067 | 0.834 | -0.131 | 0.503 |
| parahippocampal.Left | 0.059 | 0.760 | -0.396 | 0.298 | -0.087 | 0.654 |
| temporalpole.Right | -0.070 | 0.709 | 0.226 | 0.416 | -0.233 | 0.219 |
| temporalpole.Left | 0.189 | 0.299 | 0.094 | 0.739 | -0.222 | 0.238 |
| fusiform.Right | -0.078 | 0.677 | -0.026 | 0.947 | -0.148 | 0.465 |
| fusiform.Left | -0.165 | 0.357 | -0.251 | 0.398 | -0.004 | 0.985 |
| Right.Accumbens.area | 0.171 | 0.354 | 0.181 | 0.547 | 0.218 | 0.28 |
| Left.Accumbens.area | 0.086 | 0.640 | 0.205 | 0.440 | 0.279 | 0.147 |
| Right.Amygdala | -0.023 | 0.900 | 0.474 | 0.054 | -0.032 | 0.874 |
| Left.Amygdala | -0.255 | 0.139 | 0.123 | 0.691 | 0.144 | 0.474 |
| Right.Caudate | 0.173 | 0.356 | -0.192 | 0.542 | -0.250 | 0.198 |
| Left.Caudate | 0.166 | 0.372 | -0.264 | 0.371 | -0.257 | 0.204 |
| Right.Hippocampus | -0.181 | 0.363 | -0.089 | 0.783 | 0.269 | 0.224 |
| Left.Hippocampus | -0.054 | 0.773 | -0.036 | 0.907 | 0.225 | 0.277 |

Anxiety

| superiorfrontal.Right | AD_b  -0.062 | AD_p no  0.718 | on.AD_b non.AD_p  0.060 0.850 | | MCI_b  -0.241 | MCI_p  0.236 |
| --- | --- | --- | --- | --- | --- | --- |
| superiorfrontal.Left | 0.005 | 0.976 | -0.111 | 0.740 | -0.121 | 0.577 |
| caudalmiddlefrontal.Right | -0.109 | 0.508 | 0.282 | 0.352 | -0.346 | 0.106 |
| caudalmiddlefrontal.Left | -0.127 | 0.481 | 0.011 | 0.976 | -0.283 | 0.186 |
| rostralmiddlefrontal.Right | -0.020 | 0.902 | -0.163 | 0.596 | -0.188 | 0.388 |
| rostralmiddlefrontal.Left | -0.049 | 0.763 | -0.232 | 0.439 | -0.076 | 0.72 |
| parsopercularis.Right | -0.154 | 0.351 | 0.086 | 0.803 | -0.081 | 0.682 |
| parsopercularis.Left | -0.236 | 0.160 | 0.121 | 0.720 | -0.031 | 0.888 |
| parsorbitalis.Right | -0.177 | 0.263 | 0.100 | 0.748 | -0.171 | 0.38 |
| parsorbitalis.Left | -0.250 | 0.116 | -0.030 | 0.923 | 0.192 | 0.292 |
| parstriangularis.Right | -0.249 | 0.139 | -0.053 | 0.862 | -0.207 | 0.296 |
| parstriangularis.Left | -0.084 | 0.607 | -0.069 | 0.826 | 0.110 | 0.58 |
| lateralorbitofrontal.Right | 0.027 | 0.876 | -0.392 | 0.225 | -0.089 | 0.634 |
| lateralorbitofrontal.Left | -0.079 | 0.630 | -0.135 | 0.694 | 0.107 | 0.583 |
| medialorbitofrontal.Right | 0.034 | 0.833 | -0.296 | 0.340 | -0.017 | 0.925 |
| medialorbitofrontal.Left | -0.075 | 0.646 | -0.103 | 0.737 | -0.098 | 0.627 |
| frontalpole.Right | 0.259 | 0.113 | -0.330 | 0.240 | -0.082 | 0.654 |
| frontalpole.Left | 0.051 | 0.757 | -0.204 | 0.511 | 0.101 | 0.588 |
| precentral.Right | -0.032 | 0.858 | 0.411 | 0.195 | -0.285 | 0.176 |
| precentral.Left | -0.073 | 0.677 | 0.303 | 0.372 | -0.001 | 0.995 |
| paracentral.Right | -0.045 | 0.800 | 0.144 | 0.689 | -0.054 | 0.778 |
| paracentral.Left | 0.129 | 0.423 | 0.047 | 0.892 | 0.003 | 0.988 |
| rostralanteriorcingulate.Right | 0.126 | 0.434 | -0.214 | 0.530 | -0.049 | 0.802 |
| rostralanteriorcingulate.Left | -0.169 | 0.289 | -0.292 | 0.335 | -0.228 | 0.291 |
| caudalanteriorcingulate.Right | 0.201 | 0.210 | 0.119 | 0.676 | -0.144 | 0.456 |
| caudalanteriorcingulate.Left | -0.118 | 0.462 | -0.287 | 0.368 | -0.127 | 0.497 |
| posteriorcingulate.Right | 0.127 | 0.456 | -0.128 | 0.681 | -0.069 | 0.715 |
| posteriorcingulate.Left | -0.121 | 0.451 | -0.228 | 0.473 | 0.272 | 0.177 |
| isthmuscingulate.Right | -0.162 | 0.355 | 0.018 | 0.956 | -0.088 | 0.659 |
| isthmuscingulate.Left | 0.001 | 0.998 | -0.023 | 0.945 | 0.449 | 0.024 *** |
| insula.Right | -0.119 | 0.486 | 0.282 | 0.420 | -0.051 | 0.807 |
| insula.Left | -0.131 | 0.443 | -0.062 | 0.848 | -0.129 | 0.562 |
| entorhinal.Right | 0.036 | 0.836 | 0.425 | 0.175 | 0.055 | 0.779 |
| entorhinal.Left | -0.074 | 0.668 | 0.382 | 0.223 | -0.014 | 0.941 |
| parahippocampal.Right | -0.061 | 0.724 | 0.141 | 0.674 | 0.012 | 0.952 |
| parahippocampal.Left | -0.085 | 0.637 | 0.047 | 0.909 | 0.003 | 0.987 |
| temporalpole.Right | 0.062 | 0.722 | 0.479 | 0.086 | 0.025 | 0.896 |
| temporalpole.Left | 0.068 | 0.690 | 0.255 | 0.386 | -0.004 | 0.983 |
| fusiform.Right | 0.248 | 0.152 | -0.069 | 0.871 | -0.055 | 0.788 |
| fusiform.Left | 0.140 | 0.402 | 0.101 | 0.750 | 0.302 | 0.2 |
| Right.Accumbens.area | 0.146 | 0.397 | -0.101 | 0.751 | 0.109 | 0.597 |
| Left.Accumbens.area | 0.009 | 0.960 | 0.282 | 0.310 | 0.060 | 0.764 |
| Right.Amygdala | 0.218 | 0.203 | 0.443 | 0.096 | -0.229 | 0.257 |
| Left.Amygdala | 0.127 | 0.437 | 0.232 | 0.477 | -0.109 | 0.594 |
| Right.Caudate | 0.057 | 0.745 | 0.096 | 0.775 | -0.436 | 0.021 *** |
| Left.Caudate | -0.063 | 0.719 | -0.022 | 0.944 | -0.353 | 0.08 |
| Right.Hippocampus | -0.045 | 0.808 | -0.360 | 0.278 | -0.249 | 0.269 |
| Left.Hippocampus | 0.038 | 0.828 | -0.003 | 0.992 | 0.063 | 0.767 |

Euphoria

| superiorfrontal.Right | AD_b  -0.116 | AD_p no  0.525 | on.AD_b non.AD_p  -0.156 0.609 | | MCI_b  -0.380 | MCI_p  0.09 |
| --- | --- | --- | --- | --- | --- | --- |
| superiorfrontal.Left | -0.161 | 0.367 | -0.178 | 0.581 | -0.321 | 0.179 |
| caudalmiddlefrontal.Right | 0.067 | 0.702 | -0.119 | 0.690 | -0.580 | 0.012 *** |
| caudalmiddlefrontal.Left | -0.045 | 0.814 | -0.439 | 0.208 | -0.117 | 0.633 |
| rostralmiddlefrontal.Right | -0.073 | 0.672 | -0.125 | 0.676 | -0.421 | 0.076 |
| rostralmiddlefrontal.Left | -0.122 | 0.482 | -0.182 | 0.533 | -0.381 | 0.097 |
| parsopercularis.Right | -0.085 | 0.632 | -0.177 | 0.595 | -0.220 | 0.315 |
| parsopercularis.Left | -0.183 | 0.309 | -0.203 | 0.531 | 0.015 | 0.95 |
| parsorbitalis.Right | -0.132 | 0.436 | -0.107 | 0.723 | -0.342 | 0.11 |
| parsorbitalis.Left | 0.010 | 0.954 | 0.002 | 0.994 | -0.294 | 0.145 |
| parstriangularis.Right | 0.044 | 0.811 | -0.075 | 0.800 | -0.122 | 0.585 |
| parstriangularis.Left | -0.047 | 0.785 | -0.207 | 0.490 | -0.107 | 0.63 |
| lateralorbitofrontal.Right | 0.028 | 0.876 | -0.049 | 0.880 | -0.310 | 0.128 |
| lateralorbitofrontal.Left | -0.326 | 0.055 | 0.375 | 0.247 | -0.215 | 0.318 |
| medialorbitofrontal.Right | -0.098 | 0.566 | 0.152 | 0.620 | -0.257 | 0.205 |
| medialorbitofrontal.Left | -0.121 | 0.486 | 0.244 | 0.404 | -0.284 | 0.199 |
| frontalpole.Right | -0.167 | 0.344 | -0.053 | 0.852 | -0.026 | 0.898 |
| frontalpole.Left | -0.308 | 0.073 | -0.164 | 0.588 | -0.162 | 0.437 |
| precentral.Right | 0.237 | 0.205 | -0.044 | 0.891 | -0.359 | 0.125 |
| precentral.Left | 0.241 | 0.191 | 0.130 | 0.698 | -0.294 | 0.238 |
| paracentral.Right | 0.156 | 0.407 | -0.174 | 0.617 | -0.313 | 0.135 |
| paracentral.Left | 0.147 | 0.392 | 0.035 | 0.916 | -0.234 | 0.288 |
| rostralanteriorcingulate.Right | 0.100 | 0.562 | -0.062 | 0.852 | -0.415 | 0.047 *** |
| rostralanteriorcingulate.Left | -0.130 | 0.446 | -0.179 | 0.547 | -0.243 | 0.316 |
| caudalanteriorcingulate.Right | -0.086 | 0.617 | 0.137 | 0.622 | -0.077 | 0.721 |
| caudalanteriorcingulate.Left | 0.100 | 0.560 | -0.180 | 0.564 | -0.059 | 0.778 |
| posteriorcingulate.Right | 0.116 | 0.520 | 0.241 | 0.422 | -0.192 | 0.363 |
| posteriorcingulate.Left | 0.025 | 0.886 | 0.082 | 0.794 | -0.159 | 0.489 |
| isthmuscingulate.Right | -0.058 | 0.759 | 0.260 | 0.406 | -0.144 | 0.519 |
| isthmuscingulate.Left | 0.047 | 0.785 | -0.223 | 0.479 | 0.046 | 0.846 |
| insula.Right | 0.167 | 0.358 | 0.236 | 0.489 | -0.357 | 0.116 |
| insula.Left | 0.084 | 0.645 | 0.224 | 0.468 | -0.110 | 0.659 |
| entorhinal.Right | 0.198 | 0.279 | 0.187 | 0.553 | -0.525 | 0.01 *** |
| entorhinal.Left | 0.137 | 0.457 | 0.380 | 0.211 | -0.388 | 0.059 |
| parahippocampal.Right | 0.092 | 0.619 | -0.109 | 0.738 | -0.521 | 0.012 *** |
| parahippocampal.Left | 0.322 | 0.087 | -0.353 | 0.369 | -0.332 | 0.125 |
| temporalpole.Right | 0.228 | 0.216 | 0.326 | 0.247 | -0.673 | 0 *** |
| temporalpole.Left | -0.095 | 0.604 | 0.259 | 0.364 | -0.480 | 0.018 *** |
| fusiform.Right | 0.195 | 0.293 | 0.459 | 0.249 | -0.539 | 0.012 *** |
| fusiform.Left | -0.027 | 0.878 | 0.072 | 0.817 | -0.355 | 0.179 |
| Right.Accumbens.area | 0.015 | 0.938 | 0.098 | 0.752 | -0.370 | 0.1 |
| Left.Accumbens.area | -0.191 | 0.295 | 0.222 | 0.415 | -0.219 | 0.324 |
| Right.Amygdala | 0.283 | 0.120 | 0.162 | 0.553 | -0.714 | 0 *** |
| Left.Amygdala | 0.128 | 0.462 | 0.439 | 0.150 | -0.540 | 0.011 *** |
| Right.Caudate | -0.118 | 0.528 | -0.354 | 0.264 | -0.216 | 0.331 |
| Left.Caudate | -0.177 | 0.340 | -0.168 | 0.585 | -0.294 | 0.201 |
| Right.Hippocampus | 0.069 | 0.728 | -0.143 | 0.665 | -0.493 | 0.043 *** |
| Left.Hippocampus | -0.203 | 0.272 | 0.094 | 0.765 | -0.207 | 0.382 |

Apathy

| AD_b | AD_p | non.AD_b | non.AD_p | MCI_b | MCI_p |
| --- | --- | --- | --- | --- | --- |
| superiorfrontal.Right -0.283 | 0.108 | -0.400 | 0.179 | -0.124 | 0.595 |
| superiorfrontal.Left -0.133 | 0.445 | -0.573 | 0.057 | -0.231 | 0.34 |
| caudalmiddlefrontal.Right -0.256 | 0.131 | -0.306 | 0.3 | -0.177 | 0.474 |
| caudalmiddlefrontal.Left -0.250 | 0.179 | -0.161 | 0.658 | -0.043 | 0.861 |
| rostralmiddlefrontal.Right -0.109 | 0.517 | -0.375 | 0.199 | -0.045 | 0.855 |
| rostralmiddlefrontal.Left -0.179 | 0.288 | -0.339 | 0.24 | 0.265 | 0.261 |
| parsopercularis.Right -0.308 | 0.069 | -0.629 | 0.041 *** | -0.263 | 0.231 |
| parsopercularis.Left -0.246 | 0.159 | -0.284 | 0.382 | 0.044 | 0.858 |
| parsorbitalis.Right -0.275 | 0.09 | -0.011 | 0.97 | 0.022 | 0.92 |
| parsorbitalis.Left -0.321 | 0.05 | 0.087 | 0.774 | -0.250 | 0.223 |
| parstriangularis.Right -0.360 | 0.037 *** | -0.364 | 0.204 | -0.082 | 0.717 |
| parstriangularis.Left -0.316  lateralorbitofrontal.Right -0.138 | 0.056  0.434 | -0.183  -0.194 | 0.546  0.55 | 0.098  -0.007 | 0.662  0.974 |
| lateralorbitofrontal.Left -0.113  medialorbitofrontal.Right 0.026 | 0.503  0.874 | -0.180  -0.333 | 0.592  0.27 | -0.245  -0.006 | 0.258  0.976 |
| medialorbitofrontal.Left -0.208 | 0.218 | -0.380 | 0.188 | -0.314 | 0.157 |
| frontalpole.Right 0.161 | 0.35 | 0.216 | 0.441 | 0.014 | 0.945 |
| frontalpole.Left 0.021 | 0.904 | -0.193 | 0.527 | -0.027 | 0.899 |
| precentral.Right -0.253 | 0.165 | -0.239 | 0.454 | -0.344 | 0.147 |
| precentral.Left -0.356 | 0.044 *** | -0.143 | 0.673 | -0.255 | 0.312 |
| paracentral.Right -0.066 | 0.722 | -0.155 | 0.66 | -0.324 | 0.124 |
| paracentral.Left -0.002 | 0.992 | -0.246 | 0.46 | -0.451 | 0.034 *** |
| rostralanteriorcingulate.Right 0.023 | 0.893 | -0.405 | 0.213 | -0.008 | 0.97 |
| rostralanteriorcingulate.Left -0.175 | 0.289 | -0.494 | 0.081 | 0.272 | 0.263 |
| caudalanteriorcingulate.Right 0.223 | 0.181 | 0.225 | 0.416 | 0.278 | 0.195 |
| caudalanteriorcingulate.Left -0.171 | 0.303 | -0.406 | 0.184 | 0.245 | 0.24 |
| posteriorcingulate.Right 0.053 | 0.767 | -0.130 | 0.671 | 0.036 | 0.865 |
| posteriorcingulate.Left -0.064 | 0.702 | -0.468 | 0.117 | -0.067 | 0.773 |
| isthmuscingulate.Right -0.203 | 0.265 | -0.445 | 0.146 | -0.259 | 0.243 |
| isthmuscingulate.Left -0.113 | 0.504 | -0.504 | 0.096 | -0.127 | 0.593 |
| insula.Right -0.169 | 0.339 | -0.427 | 0.203 | 0.177 | 0.45 |
| insula.Left 0.012 | 0.949 | -0.211 | 0.498 | 0.252 | 0.31 |
| entorhinal.Right -0.010 | 0.958 | -0.316 | 0.314 | -0.150 | 0.496 |
| entorhinal.Left -0.200  parahippocampal.Right -0.230 | 0.261  0.197 | -0.072  0.494 | 0.821  0.112 | -0.029  0.226 | 0.891  0.311 |
| parahippocampal.Left -0.275 | 0.136 | 0.409 | 0.301 | 0.061 | 0.784 |
| temporalpole.Right -0.061  temporalpole.Left 0.101 | 0.735  0.572 | -0.061  0.211 | 0.834  0.468 | 0.052  -0.264 | 0.813  0.217 |
| fusiform.Right 0.185 | 0.308 | -0.397 | 0.329 | -0.125 | 0.59 |
| fusiform.Left 0.146 | 0.401 | 0.106 | 0.734 | -0.173 | 0.522 |
| Right.Accumbens.area 0.199 | 0.266 | -0.076 | 0.809 | 0.173 | 0.458 |
| Left.Accumbens.area -0.166 | 0.353 | 0.005 | 0.986 | 0.448 | 0.037 *** |
| Right.Amygdala 0.067 | 0.71 | -0.183 | 0.506 | -0.181 | 0.431 |
| Left.Amygdala 0.188 | 0.265 | -0.236 | 0.461 | -0.193 | 0.4 |
| Right.Caudate 0.098 | 0.591 | -0.541 | 0.078 | -0.177 | 0.43 |
| Left.Caudate -0.012 | 0.949 | -0.401 | 0.18 | -0.240 | 0.303 |
| Right.Hippocampus -0.091 | 0.638 | -0.358 | 0.27 | 0.105 | 0.683 |
| Left.Hippocampus -0.066 | 0.717 | 0.188 | 0.553 | 0.069 | 0.774 |

Disinhibition

| AD_b | AD_p | non.AD_b | non.AD_p | MCI_b | MCI_p |
| --- | --- | --- | --- | --- | --- |
| superiorfrontal.Right -0.199 | 0.27 | 0.116 | 0.732 | -0.248 | 0.3 |
| superiorfrontal.Left -0.082 | 0.642 | 0.058 | 0.871 | -0.193 | 0.444 |
| caudalmiddlefrontal.Right -0.103 | 0.553 | 0.271 | 0.405 | -0.464 | 0.061 |
| caudalmiddlefrontal.Left -0.094 | 0.62 | 0.278 | 0.481 | -0.133 | 0.602 |
| rostralmiddlefrontal.Right -0.112 | 0.509 | -0.079 | 0.812 | -0.186 | 0.466 |
| rostralmiddlefrontal.Left -0.168 | 0.327 | -0.078 | 0.811 | -0.055 | 0.824 |
| parsopercularis.Right -0.220 | 0.205 | 0.205 | 0.577 | 0.070 | 0.764 |
| parsopercularis.Left -0.259 | 0.143 | 0.294 | 0.407 | 0.077 | 0.764 |
| parsorbitalis.Right -0.040 | 0.813 | 0.327 | 0.315 | -0.068 | 0.768 |
| parsorbitalis.Left -0.093 | 0.587 | 0.161 | 0.627 | -0.032 | 0.88 |
| parstriangularis.Right -0.235  parstriangularis.Left -0.023  lateralorbitofrontal.Right 0.162 | 0.187  0.894  0.366 | 0.155  0.003  -0.172 | 0.632  0.994  0.629 | -0.034  0.237  -0.114 | 0.886  0.303  0.601 |
| lateralorbitofrontal.Left -0.211  medialorbitofrontal.Right 0.074 | 0.215  0.658 | -0.027  -0.449 | 0.941  0.166 | -0.114  -0.010 | 0.615  0.963 |
| medialorbitofrontal.Left -0.072 | 0.675 | -0.225 | 0.489 | -0.229 | 0.326 |
| frontalpole.Right 0.097 | 0.581 | -0.225 | 0.463 | -0.049 | 0.818 |
| frontalpole.Left -0.025 | 0.885 | -0.051 | 0.880 | -0.021 | 0.924 |
| precentral.Right -0.134 | 0.472 | 0.467 | 0.166 | -0.373 | 0.128 |
| precentral.Left -0.009 | 0.959 | 0.392 | 0.277 | -0.247 | 0.346 |
| paracentral.Right -0.002 | 0.992 | 0.427 | 0.255 | -0.343 | 0.116 |
| paracentral.Left 0.120 | 0.482 | 0.177 | 0.628 | -0.296 | 0.195 |
| rostralanteriorcingulate.Right 0.038 | 0.825 | -0.352 | 0.327 | -0.240 | 0.288 |
| rostralanteriorcingulate.Left 0.220 | 0.188 | -0.149 | 0.652 | 0.251 | 0.322 |
| caudalanteriorcingulate.Right 0.133 | 0.437 | 0.309 | 0.302 | 0.096 | 0.673 |
| caudalanteriorcingulate.Left 0.158 | 0.351 | 0.070 | 0.841 | 0.045 | 0.838 |
| posteriorcingulate.Right -0.067 | 0.707 | 0.045 | 0.892 | -0.037 | 0.868 |
| posteriorcingulate.Left 0.134 | 0.427 | -0.026 | 0.941 | 0.013 | 0.958 |
| isthmuscingulate.Right 0.056 | 0.764 | -0.270 | 0.435 | -0.232 | 0.314 |
| isthmuscingulate.Left 0.215 | 0.205 | -0.302 | 0.382 | -0.151 | 0.539 |
| insula.Right 0.266 | 0.136 | 0.099 | 0.795 | -0.425 | 0.071 |
| insula.Left 0.478 | 0.005 *** | -0.146 | 0.669 | -0.151 | 0.56 |
| entorhinal.Right 0.249 | 0.166 | 0.031 | 0.929 | -0.481 | 0.027 *** |
| entorhinal.Left 0.171  parahippocampal.Right 0.188 | 0.344  0.302 | 0.009  0.067 | 0.979  0.852 | -0.257  -0.199 | 0.243  0.39 |
| parahippocampal.Left 0.048 | 0.802 | 0.337 | 0.439 | -0.219 | 0.339 |
| temporalpole.Right 0.073 | 0.693 | 0.153 | 0.631 | -0.502 | 0.019 *** |
| temporalpole.Left 0.261 | 0.143 | -0.011 | 0.973 | -0.364 | 0.097 |
| fusiform.Right 0.324 | 0.073 | -0.239 | 0.596 | -0.535 | 0.018 *** |
| fusiform.Left 0.280 | 0.109 | 0.279 | 0.406 | -0.310 | 0.263 |
| Right.Accumbens.area 0.171 | 0.347 | -0.252 | 0.455 | -0.121 | 0.616 |
| Left.Accumbens.area 0.120 | 0.508 | 0.141 | 0.642 | 0.031 | 0.893 |
| Right.Amygdala 0.148 | 0.417 | 0.006 | 0.985 | -0.477 | 0.037 *** |
| Left.Amygdala 0.084 | 0.627 | -0.177 | 0.613 | -0.220 | 0.354 |
| Right.Caudate 0.284 | 0.121 | 0.042 | 0.907 | 0.051 | 0.827 |
| Left.Caudate 0.217 | 0.235 | -0.161 | 0.635 | -0.060 | 0.807 |
| Right.Hippocampus -0.200 | 0.308 | -0.534 | 0.122 | 0.139 | 0.602 |
| Left.Hippocampus -0.252 | 0.166 | -0.140 | 0.687 | 0.271 | 0.27 |

Irritability

| superiorfrontal.Right | AD_b  -0.123 | AD_p no  0.492 | on.AD_b  -0.149 | non.AD_p  0.637 | MCI_b  0.364 | MCI_p  0.1 |
| --- | --- | --- | --- | --- | --- | --- |
| superiorfrontal.Left | 0.030 | 0.863 | -0.100 | 0.764 | 0.367 | 0.116 |
| caudalmiddlefrontal.Right | -0.176 | 0.301 | 0.081 | 0.792 | 0.213 | 0.377 |
| caudalmiddlefrontal.Left | -0.063 | 0.737 | -0.072 | 0.847 | 0.189 | 0.431 |
| rostralmiddlefrontal.Right | -0.136 | 0.416 | -0.314 | 0.298 | 0.575 | 0.011 *** |
| rostralmiddlefrontal.Left | -0.008 | 0.962 | 0.008 | 0.979 | 0.526 | 0.016 *** |
| parsopercularis.Right | -0.261 | 0.125 | 0.024 | 0.945 | 0.500 | 0.014 *** |
| parsopercularis.Left | 0.055 | 0.754 | 0.159 | 0.636 | 0.354 | 0.133 |
| parsorbitalis.Right | -0.263 | 0.106 | -0.006 | 0.984 | 0.387 | 0.064 |
| parsorbitalis.Left | -0.203 | 0.224 | -0.123 | 0.691 | 0.244 | 0.223 |
| parstriangularis.Right | -0.223 | 0.204 | -0.034 | 0.912 | 0.383 | 0.073 |
| parstriangularis.Left | -0.001 | 0.994 | 0.048 | 0.877 | 0.466 | 0.025 *** |
| lateralorbitofrontal.Right | -0.193 | 0.271 | -0.113 | 0.734 | 0.214 | 0.292 |
| lateralorbitofrontal.Left | -0.022 | 0.895 | 0.004 | 0.991 | 0.317 | 0.13 |
| medialorbitofrontal.Right | 0.067 | 0.685 | -0.634 | 0.025 *** | 0.302 | 0.128 |
| medialorbitofrontal.Left | -0.008 | 0.961 | -0.110 | 0.72 | 0.194 | 0.379 |
| frontalpole.Right | 0.151 | 0.379 | -0.464 | 0.086 | 0.326 | 0.096 |
| frontalpole.Left | 0.238 | 0.159 | -0.230 | 0.457 | 0.348 | 0.082 |
| precentral.Right | -0.176 | 0.337 | 0.025 | 0.941 | 0.084 | 0.723 |
| precentral.Left | -0.023 | 0.897 | -0.026 | 0.94 | 0.104 | 0.678 |
| paracentral.Right | -0.122 | 0.506 | -0.043 | 0.905 | 0.071 | 0.736 |
| paracentral.Left | 0.100 | 0.551 | -0.003 | 0.993 | 0.010 | 0.965 |
| rostralanteriorcingulate.Right | 0.170 | 0.311 | -0.596 | 0.06 | 0.178 | 0.407 |
| rostralanteriorcingulate.Left | 0.015 | 0.929 | -0.294 | 0.331 | 0.370 | 0.115 |
| caudalanteriorcingulate.Right | 0.143 | 0.392 | 0.136 | 0.632 | 0.173 | 0.416 |
| caudalanteriorcingulate.Left | 0.099 | 0.554 | 0.157 | 0.627 | -0.043 | 0.835 |
| posteriorcingulate.Right | 0.005 | 0.976 | 0.054 | 0.863 | 0.090 | 0.666 |
| posteriorcingulate.Left | 0.007 | 0.967 | 0.040 | 0.902 | 0.165 | 0.466 |
| isthmuscingulate.Right | -0.157 | 0.391 | -0.189 | 0.562 | -0.164 | 0.455 |
| isthmuscingulate.Left | 0.124 | 0.460 | -0.426 | 0.176 | -0.153 | 0.509 |
| insula.Right | -0.155 | 0.383 | -0.256 | 0.465 | 0.104 | 0.651 |
| insula.Left | -0.121 | 0.497 | -0.100 | 0.756 | 0.208 | 0.394 |
| entorhinal.Right | -0.140 | 0.434 | -0.101 | 0.757 | 0.003 | 0.99 |
| entorhinal.Left | -0.191 | 0.284 | -0.204 | 0.526 | 0.099 | 0.639 |
| parahippocampal.Right | 0.049 | 0.787 | -0.088 | 0.793 | 0.098 | 0.657 |
| parahippocampal.Left | 0.026 | 0.888 | -0.122 | 0.768 | -0.179 | 0.41 |
| temporalpole.Right | -0.339 | 0.056 | -0.224 | 0.447 | 0.147 | 0.494 |
| temporalpole.Left | -0.131 | 0.462 | -0.020 | 0.947 | 0.123 | 0.565 |
| fusiform.Right | -0.099 | 0.588 | -0.516 | 0.205 | 0.129 | 0.572 |
| fusiform.Left | -0.008 | 0.963 | -0.086 | 0.788 | 0.141 | 0.595 |
| Right.Accumbens.area | -0.029 | 0.873 | -0.071 | 0.825 | 0.331 | 0.139 |
| Left.Accumbens.area | -0.114 | 0.525 | 0.216 | 0.442 | 0.402 | 0.058 |
| Right.Amygdala | -0.092 | 0.609 | -0.144 | 0.61 | 0.053 | 0.815 |
| Left.Amygdala | -0.050 | 0.769 | -0.205 | 0.53 | 0.155 | 0.491 |
| Right.Caudate | 0.097 | 0.598 | 0.008 | 0.98 | 0.377 | 0.078 |
| Left.Caudate | 0.083 | 0.647 | -0.017 | 0.957 | 0.324 | 0.151 |
| Right.Hippocampus | -0.060 | 0.759 | -0.307 | 0.359 | 0.206 | 0.411 |
| Left.Hippocampus | 0.035 | 0.849 | -0.370 | 0.241 | 0.098 | 0.677 |

Aberrant motor behavior

| AD_b | AD_p | non.AD_b | non.AD_p | MCI_b | MCI_p |
| --- | --- | --- | --- | --- | --- |
| superiorfrontal.Right -0.098 | 0.584 | -0.067 | 0.805 | -0.457 | 0.044 *** |
| superiorfrontal.Left 0.045 | 0.795 | -0.212 | 0.455 | -0.345 | 0.159 |
| caudalmiddlefrontal.Right 0.020 | 0.907 | 0.072 | 0.786 | -0.429 | 0.08 |
| caudalmiddlefrontal.Left 0.011 | 0.951 | -0.018 | 0.954 | -0.223 | 0.37 |
| rostralmiddlefrontal.Right -0.030 | 0.859 | -0.212 | 0.417 | -0.533 | 0.026 *** |
| rostralmiddlefrontal.Left -0.081 | 0.635 | -0.305 | 0.226 | -0.466 | 0.045 *** |
| parsopercularis.Right -0.016 | 0.927 | -0.114 | 0.699 | -0.277 | 0.217 |
| parsopercularis.Left -0.143 | 0.418 | -0.029 | 0.921 | -0.252 | 0.311 |
| parsorbitalis.Right -0.191 | 0.245 | -0.027 | 0.919 | -0.280 | 0.207 |
| parsorbitalis.Left -0.194 | 0.245 | -0.209 | 0.426 | 0.021 | 0.923 |
| parstriangularis.Right -0.084 | 0.637 | -0.045 | 0.863 | -0.343 | 0.128 |
| parstriangularis.Left -0.094  lateralorbitofrontal.Right 0.017  lateralorbitofrontal.Left -0.215  medialorbitofrontal.Right 0.007 | 0.578  0.925  0.2  0.965 | -0.158  -0.323  -0.321  -0.111 | 0.552  0.245  0.263  0.683 | -0.199  -0.322  -0.317  -0.253 | 0.381  0.124  0.147  0.225 |
| medialorbitofrontal.Left 0.081 | 0.636 | -0.131 | 0.616 | -0.273 | 0.232 |
| frontalpole.Right 0.135 | 0.433 | -0.154 | 0.533 | -0.139 | 0.508 |
| frontalpole.Left 0.074 | 0.664 | -0.134 | 0.616 | -0.195 | 0.363 |
| precentral.Right -0.018 | 0.921 | 0.165 | 0.558 | -0.349 | 0.149 |
| precentral.Left 0.058 | 0.748 | 0.033 | 0.911 | -0.125 | 0.632 |
| paracentral.Right -0.109 | 0.556 | 0.188 | 0.541 | -0.199 | 0.364 |
| paracentral.Left 0.147 | 0.38 | -0.014 | 0.962 | -0.023 | 0.92 |
| rostralanteriorcingulate.Right 0.116 | 0.492 | 0.169 | 0.563 | -0.503 | 0.017 *** |
| rostralanteriorcingulate.Left -0.114 | 0.494 | -0.168 | 0.524 | -0.044 | 0.861 |
| caudalanteriorcingulate.Right 0.056 | 0.742 | -0.090 | 0.714 | -0.032 | 0.886 |
| caudalanteriorcingulate.Left -0.088 | 0.599 | -0.195 | 0.479 | -0.030 | 0.889 |
| posteriorcingulate.Right -0.072 | 0.686 | -0.306 | 0.241 | 0.003 | 0.988 |
| posteriorcingulate.Left -0.027 | 0.873 | -0.367 | 0.165 | 0.173 | 0.464 |
| isthmuscingulate.Right -0.286 | 0.114 | -0.065 | 0.818 | 0.081 | 0.723 |
| isthmuscingulate.Left -0.264 | 0.111 | 0.056 | 0.842 | 0.502 | 0.029 *** |
| insula.Right -0.016 | 0.928 | 0.076 | 0.803 | 0.004 | 0.985 |
| insula.Left -0.134 | 0.454 | -0.009 | 0.974 | 0.020 | 0.938 |
| entorhinal.Right 0.020 | 0.91 | 0.347 | 0.198 | -0.012 | 0.958 |
| entorhinal.Left -0.362  parahippocampal.Right -0.024 | 0.038 *** 0.893 | 0.285  0.208 | 0.293  0.464 | -0.012  -0.077 | 0.956  0.737 |
| parahippocampal.Left -0.197 | 0.289 | 0.622 | 0.056 | -0.048 | 0.834 |
| temporalpole.Right -0.038 | 0.833 | 0.479 | 0.039 *** | -0.275 | 0.213 |
| temporalpole.Left -0.236 | 0.181 | 0.249 | 0.322 | 0.050 | 0.824 |
| fusiform.Right 0.016 | 0.931 | 0.084 | 0.818 | -0.356 | 0.124 |
| fusiform.Left 0.015 | 0.931 | 0.281 | 0.293 | -0.049 | 0.86 |
| Right.Accumbens.area 0.066 | 0.715 | -0.185 | 0.495 | -0.234 | 0.322 |
| Left.Accumbens.area -0.138 | 0.442 | 0.042 | 0.865 | -0.436 | 0.048 *** |
| Right.Amygdala 0.087 | 0.628 | 0.288 | 0.222 | -0.252 | 0.281 |
| Left.Amygdala 0.032 | 0.849 | 0.141 | 0.616 | -0.092 | 0.696 |
| Right.Caudate -0.073 | 0.692 | 0.117 | 0.685 | -0.186 | 0.419 |
| Left.Caudate 0.034 | 0.852 | 0.095 | 0.726 | -0.236 | 0.321 |
| Right.Hippocampus 0.128 | 0.511 | -0.470 | 0.085 | 0.050 | 0.849 |
| Left.Hippocampus 0.000 | 1 | 0.008 | 0.978 | 0.277 | 0.251 |

Night-time behavior disturbances

| AD_b | AD_p | non.AD_b | non.AD_p | MCI_b | MCI_p |
| --- | --- | --- | --- | --- | --- |
| superiorfrontal.Right -0.209 | 0.226 | -0.177 | 0.556 | -0.033 | 0.891 |
| superiorfrontal.Left -0.221 | 0.198 | -0.146 | 0.649 | 0.083 | 0.739 |
| caudalmiddlefrontal.Right -0.257 | 0.116 | -0.053 | 0.857 | -0.124 | 0.625 |
| caudalmiddlefrontal.Left -0.241 | 0.192 | -0.312 | 0.375 | 0.134 | 0.591 |
| rostralmiddlefrontal.Right -0.103 | 0.527 | -0.466 | 0.094 | -0.007 | 0.979 |
| rostralmiddlefrontal.Left -0.146 | 0.374 | -0.180 | 0.533 | -0.070 | 0.774 |
| parsopercularis.Right -0.192 | 0.256 | -0.006 | 0.985 | 0.381 | 0.083 |
| parsopercularis.Left -0.216 | 0.212 | -0.075 | 0.817 | 0.393 | 0.108 |
| parsorbitalis.Right -0.204 | 0.2 | -0.287 | 0.326 | 0.228 | 0.309 |
| parsorbitalis.Left -0.210 | 0.193 | -0.248 | 0.397 | 0.055 | 0.794 |
| parstriangularis.Right -0.254 | 0.138 | -0.127 | 0.661 | 0.205 | 0.37 |
| parstriangularis.Left -0.145 | 0.375 | -0.488 | 0.081 | 0.266 | 0.238 |
| lateralorbitofrontal.Right -0.080  lateralorbitofrontal.Left -0.064 | 0.645  0.699 | -0.629  -0.428 | 0.029 *** 0.176 | -0.016  0.213 | 0.941  0.337 |
| medialorbitofrontal.Right -0.110 | 0.495 | -0.319 | 0.282 | -0.109 | 0.607 |
| medialorbitofrontal.Left -0.040 | 0.808 | -0.201 | 0.49 | 0.288 | 0.205 |
| frontalpole.Right 0.033 | 0.846 | -0.610 | 0.011 *** | -0.074 | 0.725 |
| frontalpole.Left -0.098 | 0.567 | -0.251 | 0.396 | 0.215 | 0.314 |
| precentral.Right -0.178 | 0.317 | -0.152 | 0.629 | -0.003 | 0.989 |
| precentral.Left -0.328 | 0.056 | -0.361 | 0.263 | 0.080 | 0.757 |
| paracentral.Right -0.363 | 0.039 *** | -0.225 | 0.511 | 0.038 | 0.862 |
| paracentral.Left -0.094 | 0.565 | 0.142 | 0.665 | 0.085 | 0.709 |
| rostralanteriorcingulate.Right -0.020 | 0.903 | -0.171 | 0.602 | 0.114 | 0.61 |
| rostralanteriorcingulate.Left 0.040 | 0.804 | -0.309 | 0.286 | -0.184 | 0.461 |
| caudalanteriorcingulate.Right -0.089 | 0.6 | -0.235 | 0.385 | 0.336 | 0.121 |
| caudalanteriorcingulate.Left 0.070 | 0.666 | 0.009 | 0.976 | -0.037 | 0.862 |
| posteriorcingulate.Right 0.013 | 0.941 | 0.131 | 0.661 | 0.117 | 0.592 |
| posteriorcingulate.Left -0.001 | 0.997 | 0.022 | 0.943 | 0.169 | 0.474 |
| isthmuscingulate.Right -0.110 | 0.536 | -0.056 | 0.859 | 0.028 | 0.904 |
| isthmuscingulate.Left -0.037 | 0.822 | 0.076 | 0.809 | 0.069 | 0.775 |
| insula.Right -0.004 | 0.981 | -0.371 | 0.263 | -0.087 | 0.717 |
| insula.Left -0.172 | 0.318 | -0.361 | 0.226 | -0.055 | 0.831 |
| entorhinal.Right -0.044 | 0.807 | -0.002 | 0.996 | -0.143 | 0.524 |
| entorhinal.Left 0.114 | 0.54 | -0.117 | 0.708 | 0.071 | 0.746 |
| parahippocampal.Right -0.028 | 0.873 | -0.196 | 0.541 | 0.011 | 0.964 |
| parahippocampal.Left -0.117  temporalpole.Right 0.019 | 0.518  0.913 | -0.421  -0.054 | 0.276  0.851 | 0.180  -0.252 | 0.427  0.254 |
| temporalpole.Left -0.007 | 0.966 | -0.183 | 0.52 | -0.247 | 0.26 |
| fusiform.Right 0.027 | 0.881 | -0.216 | 0.593 | -0.158 | 0.505 |
| fusiform.Left 0.040 | 0.813 | -0.170 | 0.577 | 0.027 | 0.923 |
| Right.Accumbens.area 0.278 | 0.11 | -0.257 | 0.394 | -0.334 | 0.152 |
| Left.Accumbens.area 0.145 | 0.407 | 0.028 | 0.92 | -0.123 | 0.592 |
| Right.Amygdala 0.195 | 0.268 | -0.120 | 0.659 | -0.507 | 0.023 *** |
| Left.Amygdala -0.077 | 0.643 | -0.098 | 0.757 | -0.319 | 0.166 |
| Right.Caudate 0.300 | 0.099 | 0.269 | 0.398 | 0.108 | 0.64 |
| Left.Caudate 0.152 | 0.405 | 0.311 | 0.297 | 0.180 | 0.451 |
| Right.Hippocampus 0.164 | 0.391 | -0.330 | 0.302 | -0.229 | 0.378 |
| Left.Hippocampus 0.180 | 0.309 | -0.532 | 0.066 | 0.026 | 0.917 |

Eating abnormalities

| AD_b | AD_p | non.AD_b | non.AD_p | MCI_b | MCI_p |
| --- | --- | --- | --- | --- | --- |
| superiorfrontal.Right -0.210 | 0.247 | -0.206 | 0.502 | -0.349 | 0.129 |
| superiorfrontal.Left -0.032 | 0.859 | -0.423 | 0.178 | -0.267 | 0.276 |
| caudalmiddlefrontal.Right -0.170 | 0.328 | -0.204 | 0.496 | -0.532 | 0.025 *** |
| caudalmiddlefrontal.Left -0.081 | 0.675 | -0.260 | 0.471 | -0.093 | 0.71 |
| rostralmiddlefrontal.Right -0.125 | 0.463 | -0.195 | 0.515 | -0.375 | 0.125 |
| rostralmiddlefrontal.Left -0.133 | 0.443 | -0.155 | 0.601 | -0.306 | 0.197 |
| parsopercularis.Right -0.347 | 0.044 *** | -0.476 | 0.139 | -0.189 | 0.4 |
| parsopercularis.Left -0.334 | 0.058 | -0.229 | 0.482 | -0.199 | 0.424 |
| parsorbitalis.Right -0.210 | 0.211 | 0.088 | 0.771 | -0.304 | 0.166 |
| parsorbitalis.Left -0.265 | 0.117 | 0.016 | 0.958 | -0.119 | 0.57 |
| parstriangularis.Right -0.331 | 0.062 | -0.322 | 0.265 | 0.027 | 0.908 |
| parstriangularis.Left -0.218 | 0.203 | -0.112 | 0.713 | -0.124 | 0.583 |
| lateralorbitofrontal.Right -0.046 | 0.801 | -0.391 | 0.215 | -0.180 | 0.394 |
| lateralorbitofrontal.Left -0.229 | 0.181 | -0.512 | 0.106 | -0.226 | 0.305 |
| medialorbitofrontal.Right -0.156 | 0.355 | -0.025 | 0.935 | -0.229 | 0.271 |
| medialorbitofrontal.Left -0.151 | 0.384 | -0.516 | 0.063 | -0.260 | 0.251 |
| frontalpole.Right 0.069 | 0.697 | 0.271 | 0.33 | -0.169 | 0.417 |
| frontalpole.Left 0.020 | 0.911 | -0.002 | 0.995 | -0.213 | 0.316 |
| precentral.Right 0.011 | 0.953 | -0.216 | 0.5 | -0.238 | 0.328 |
| precentral.Left -0.114 | 0.539 | -0.339 | 0.306 | -0.211 | 0.41 |
| paracentral.Right -0.008 | 0.966 | -0.345 | 0.318 | -0.293 | 0.173 |
| paracentral.Left 0.067 | 0.697 | -0.146 | 0.663 | -0.233 | 0.3 |
| rostralanteriorcingulate.Right 0.010 | 0.953 | -0.255 | 0.442 | -0.269 | 0.221 |
| rostralanteriorcingulate.Left -0.150 | 0.375 | -0.237 | 0.428 | -0.156 | 0.532 |
| caudalanteriorcingulate.Right 0.005 | 0.978 | -0.026 | 0.928 | -0.007 | 0.974 |
| caudalanteriorcingulate.Left 0.040 | 0.815 | -0.374 | 0.223 | 0.096 | 0.654 |
| posteriorcingulate.Right 0.033 | 0.853 | -0.192 | 0.527 | -0.062 | 0.776 |
| posteriorcingulate.Left -0.048 | 0.779 | -0.528 | 0.071 | -0.065 | 0.783 |
| isthmuscingulate.Right -0.179 | 0.338 | -0.384 | 0.215 | 0.006 | 0.978 |
| isthmuscingulate.Left -0.056 | 0.745 | -0.046 | 0.887 | 0.067 | 0.782 |
| insula.Right 0.038 | 0.836 | -0.454 | 0.173 | -0.252 | 0.284 |
| insula.Left 0.015 | 0.936 | -0.639 | 0.022 *** | -0.131 | 0.606 |
| entorhinal.Right -0.119 | 0.516 | -0.480 | 0.112 | -0.499 | 0.018 *** |
| entorhinal.Left -0.168  parahippocampal.Right -0.054  parahippocampal.Left 0.109 | 0.357  0.769  0.569 | -0.445  0.550  -0.242 | 0.141  0.072  0.546 | -0.375  -0.508  -0.305 | 0.075  0.018 *** 0.169 |
| temporalpole.Right -0.005 | 0.979 | -0.444 | 0.107 | -0.668 | 0.001 *** |
| temporalpole.Left 0.019 | 0.918 | -0.198 | 0.494 | -0.476 | 0.022 *** |
| fusiform.Right 0.206 | 0.266 | -0.647 | 0.097 | -0.468 | 0.037 *** |
| fusiform.Left -0.004 | 0.984 | -0.428 | 0.151 | -0.368 | 0.172 |
| Right.Accumbens.area 0.136 | 0.459 | 0.071 | 0.822 | -0.137 | 0.562 |
| Left.Accumbens.area -0.016 | 0.929 | -0.173 | 0.531 | -0.033 | 0.885 |
| Right.Amygdala 0.152 | 0.408 | -0.128 | 0.643 | -0.626 | 0.003 *** |
| Left.Amygdala 0.241 | 0.16 | -0.481 | 0.114 | -0.443 | 0.048 *** |
| Right.Caudate 0.185 | 0.319 | -0.324 | 0.313 | -0.286 | 0.203 |
| Left.Caudate 0.082 | 0.657 | -0.247 | 0.422 | -0.350 | 0.133 |
| Right.Hippocampus -0.019 | 0.923 | 0.239 | 0.469 | -0.408 | 0.107 |
| Left.Hippocampus -0.002 | 0.994 | 0.037 | 0.909 | -0.264 | 0.271 |
